# Structure-guided discovery of novel dUTPase inhibitors with anti-*Nocardia* activity by computational design

**DOI:** 10.1080/14756366.2024.2411573

**Published:** 2024-10-10

**Authors:** Zhi-Zheng Wang, Jun Weng, Jing Qi, Xin-Xin Fu, Ban-Bin Xing, Yang Hu, Chun-Hsiang Huang, Chin-Yu Chen, Zigong Wei

**Affiliations:** a School of Life Sciences, State Key Laboratory of Biocatalysis and Enzyme Engineering, National & Local Joint Engineering Research Center of High-throughput Drug Screening Technology, Hubei University, Wuhan, PR China; b College of Life Science and Technology, Key Laboratory of Molecular Biophysics of Ministry of Education, National Engineering Research Center for Nanomedicine, Huazhong University of Science and Technology, Wuhan, PR China; c Protein Diffraction Group, Experimental Facility Division, National Synchrotron Radiation Research Center, Hsinchu, Taiwan; d Hubei Jiangxia Laboratory, Wuhan, PR China

**Keywords:** Computer-aided drug design, antibiotics, *Nocardia*, dUTPase

## Abstract

The zoonosis caused by *Nocardia* is increasing seriously. But commonly used antibiotic drugs often lead to resistance. *N. seriolae* dUTPase (*Ns*dUTPase) plays a key role in the proliferation of *Nocardia*, and was regarded as a potent drug target. However, there was little report about the *Ns*dUTPase inhibitors. In this study, we discovered a series of novel *Ns*dUTPase inhibitors to fight against *Nocardia*. The first crystal structure of *Ns*dUTPase was released, and a structure-based computational design was performed. Compounds **4b** and **12b** exhibited promising activities towards *Ns*dUTPase (IC_50_ = 0.99 μM and 0.7 μM). In addition, they showed satisfied anti-*Nocardia* activity (MIC value ranges from 0.5 to 2 mg/L) and low cytotoxicity, which were better than approved drugs oxytetracycline and florfenicol. Molecular modelling study indicated that hydrophobic interaction might be the main contribution for ligand binding. Our results suggested that *Ns*dUTPase inhibitors might be a useful way to repress *Nocardia*.

## GRAPHICAL ABSTRACT

**Figure:**
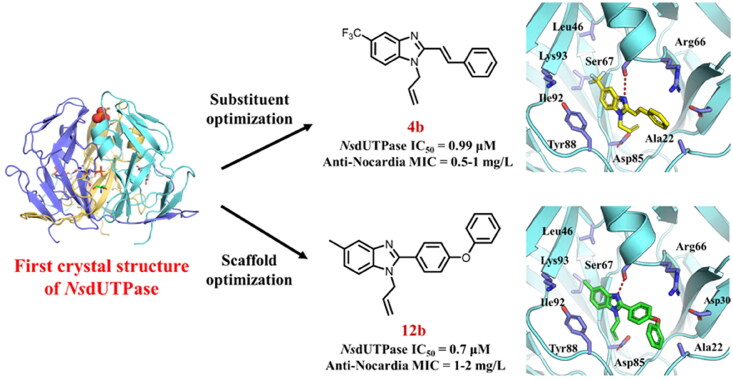

## Introduction

*Nocardia*-induced zoonosis put serious threat to the human health and aquaculture industry. The genus *Nocardia* is a group of Gram-positive branched beaded and long filamentous bacteria, which belongs to the class Actinobacteria, family Nocardiaceae^1^. *Nocardia* is well known as the cause of nocardiosis, an infectious disease that occurs in both humans and various animals^2^. Up to now, more than 50 *Nocardia* species have been reported to cause various human diseases^3^^,^^4^. Some *Nocardia* species remain formidable pathogens to the patients receiving immunosuppressive therapies for solid organ or haematopoietic stem cell transplants, haematologic and solid tissue cancers, as well as autoinflammatory conditions^5^^,^^6^. Moreover, some *Nocardia* species are known to cause nocardiosis in mammal animals^7^^,^^8^, as well as marine animals such as oysters and fish^9^^,^^10^. Among them, *N. seriolae* was identified as the main cause of fish nocardiosis^11–14^, which lead to severe economic losses in the aquaculture industry^15^^,^^16^. Based on this, drug discovery towards *Nocardia* has become an urgent need for human society.

Antibiotic drugs are the primary ways to fight bacteria, which has profoundly improved the human life. But the abuse of antibiotic drugs caused serious resistance, and the World Health Organization (WHO) has recognised this phenomenon as a major global health threat. For *Nocardia*, it showed resistance to a series of antibiotic drugs^2^. Although various types of antibiotic drugs were employed to fight against nocardiosis of human and economic species, the practice showed limited efficacy^2^. Meanwhile, applying antibiotic drugs evolves serious problems such as environmental pollution, bacterial resistance, and drug residues^17^. Therefore, developing more efficient drugs might be a promising way to combat *Nocardia*.

Deoxyuridine 5′-triphosphate nucleotidohydrolase (dUTPase) is an enzyme encoded in nearly all prokaryotes, eukaryotes, and a majority of viruses^18^. The enzyme catalyses the hydrolysis of dUTP to dUMP, eventually controlling the cellular dUTP:dTTP concentration to the level that could prevent incorporation of dUTP into DNA, which would overwhelm the DNA repair system, leading to multiple DNA strand breaks and eventually, cell death^18^. Due to the important role of dUTPase for cell viability in all organisms studied to date, including bacteria and virus, it is therefore likely that dUTPase represents a novel target that yet remains to be explored in fighting against pathogens including microbes and virus^19^^,^^20^. Recently, study also showed that dUTPase was essential in *Mycobacterium smegmatis* and could be used as a potential target for antitubercular drugs^21^. However, there are limited reports about the development of *N. seriolae* dUTPase (*Ns*dUTPase) inhibitors, which might limit the drug discovery against *Nocardia*.

In this study, we reveal that *Ns*dUTPase inhibitors could act as anti-*Nocardia* drug candidates. We expressed and purified *Ns*dUTPase in *E. coli*, and resolved its first crystal structure with a resolution of 2.12 Å. Based on this, structural-based computational design was performed, by adding hydrophobic substituents; a more potent compound **4b** was identified with the IC_50_ value of 0.99 μM. In addition, to further improve the activity, a series of new scaffold was used to optimise compound **4b** via computational fragment growing, and compound **12b** was finally obtained with the IC_50_ value of 0.70 μM. More importantly, compounds **4b** and **12b** exhibited satisfied anti-*Nocardia* activity *in vitro* (MIC values range from 0.5 to 2 mg/mL), which were better than approved antibiotic oxytetracycline and florfenicol, and they showed low cytotoxicity to human cells. Molecular modelling study indicated that hydrophobic interaction was the main contribution for the binding of inhibitors, and Arg66 and Ser67 might be the key residues. The computational approaches obviously speed up the discovery of potent inhibitors, and could well explain the activity differences of obtained compounds. Our results suggested that compounds **4b** and **12b** might be a promising lead compound to fight against *Nocardia*.

## Results and discussion

### Crystal structure characterisation of NsdUTPase

**Figure 1.**
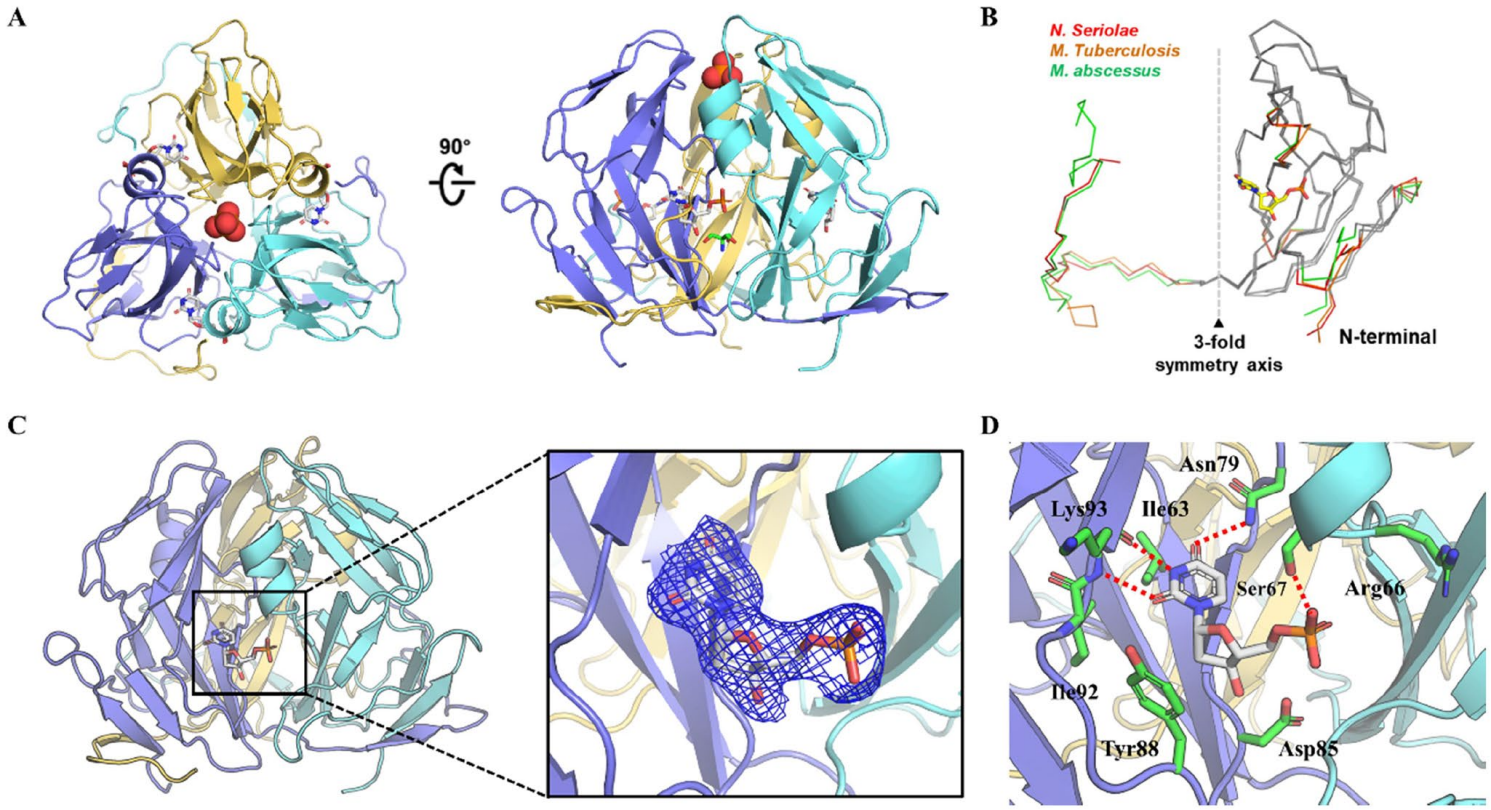
Crystal structure of *Ns*dUTPase. (A) Crystal structure of *Ns*dUTPase in front view and top view (PDB code 8HXM). (B) The alignment of *Ns*dUTPase, *Mt*dUTPase, and *Ma*dUTPase. (C) The active site of *Ns*dUTPase. The substrate dUMP was located in the cleft of two monomers. (D) The binding mode between dUMP and *Ns*dUTPase. The hydrogen bond is shown in red dotted lines.

Structure-based drug design has been widely used in the drug discovery and to achieve huge success. To discover potent *Ns*dUTPase inhibitor, we tried to obtain the crystal structure of *Ns*dUTPase. *Ns*dUTPase was expressed in *E. coli* as a recombinant protein with a C-terminal His-tag. Purified *Ns*dUTPase was crystallised in 25% (w/v) polyethylene glycol-3350 and 0.1 M Tris–HCl pH 8.5. Finally, crystal structure of *Ns*dUTPase was determined at a resolution of 2.12 Å (Table 1, PDB code 8XHM). Resolved *Ns*dUTPase crystal structure showed a classical trimer assembling, and each dUTPase monomer is an asymmetry unit folding into a jelly-roll β-barrel structure, composed of a total of 11 β-strands grouped into four antiparallel β-sheets in addition to a single short α-helix between the fourth and fifth β-sheets (Figure 1(A)). The structure alignment showed that although *Ns*dUTPase exhibited similar folding pattern with dUTPases from *M. tuberculosis* (*Mt*dUTPase) and *M. abscessus* (*Ma*dUTPase), there were still some differences between their conformations (Figure 1(B)). And drugs targeting *Mt*dUTPase might not be able to inhibit the *Ns*dUTPase. The experiment-determined crystal structure might be a powerful way to support the drug discovery targeting *Ns*dUTPase.

**Table 1.** Summary of crystallographic data collection and refinement statistics.

*Ns*dUTPase (PDB ID: 8XHM)	
*Data collection statistics* ^a^	
Space group	P 41 3 2
Cell dimensions (Å, °)	*a* = *b* = *c* = 120.81, *α* = *β* = *γ* = 90
Wavelength (Å)	0.976
Resolution range (Å)	28.47–2.12 (2.196–2.12)
Total reflections	894 780 (88 206)
Unique reflections	17 649 (1723)
Multiplicity	50.7 (51.2)
Completeness (%)	99.80 (98.80)
Mean *I*/*σ*(*I*)	36.67 (3.35)
Wilson *B*-factor (Å^2^)	52.7
*R*-merge (%)	7.5 (114)
*R*_meas_ (%)	7.6 (115)
CC_1/2_	100 (93.6)
*Refinement statistics*	
Reflections used in refinement	17 647 (1722)
Reflections used for *R*_free_	882 (86)
*R*_cryst_ (%)	18.5 (23.7)
*R*_free_ (%)	20.6 (25.3)
RMSD bond length (Å)	0.007
RMSD angle (°)	1.09
Number of non-hydrogen atoms	1127
Macromolecules	1027
Ligands	55
Solvent	45
Average *B*-factor (Å^2^)	57.5
*Ramachandran plot* (%)	
Most favoured	98.55
Allowed	1.45

^a^ Statistics for the highest resolution shell are shown in parentheses.

### Spatial organisation of the dUMP-binding pocket in NsdUTPase

To discover potent *Ns*dUTPase inhibitors, the active site of *Ns*dUTPase was further analysed. The active site was the substrate binding pocket, and there was no other binding pocket found in *Ns*dUTPase identified by fpocket. Trimeric *Ns*dUTPase contained three identical ligand binding sites located at clefts between adjacent monomers (Figure 1(C)). In other words, *Ns*dUTPase could bind with three substrates. For each single pocket, it is composed of five conserved sequence motifs. And dUMP bound in the cleft of two monomers interacts with two monomers (Figure 1(C)). The active site of *Ns*dUTPase was located in the surface, and consists of a series of hydrophobic interactions, which might be a promising binding pocket for ligand binding. In addition, there were some polar residues contained in the pocket, which could be used for improving the activity of designed compounds.

Then, we verified the detailed interactions between dUMP and *Ns*dUTPase (Figure 1(D)), the uracil group of dUMP formed hydrogen bonds with the main chain of Ile92 and Lys93 as well as the side chain of Asn79. The phosphate group of dUMP formed hydrogen bond with the side chain of Ser67. In addition, dUMP showed hydrophobic interactions with a series of surrounding residues, including Ile63, Ile92, Lys93, and Tyr88. Based on these interactions, we could find that the active site of *Ns*dUTPase was a hydrophobic pocket and also contained some polar residues. Therefore, it could be a promising binding pocket for the drug discovery.

### Identification of benzoimidazole as the scaffold of NsdUTPase inhibitors

**Figure 2.**
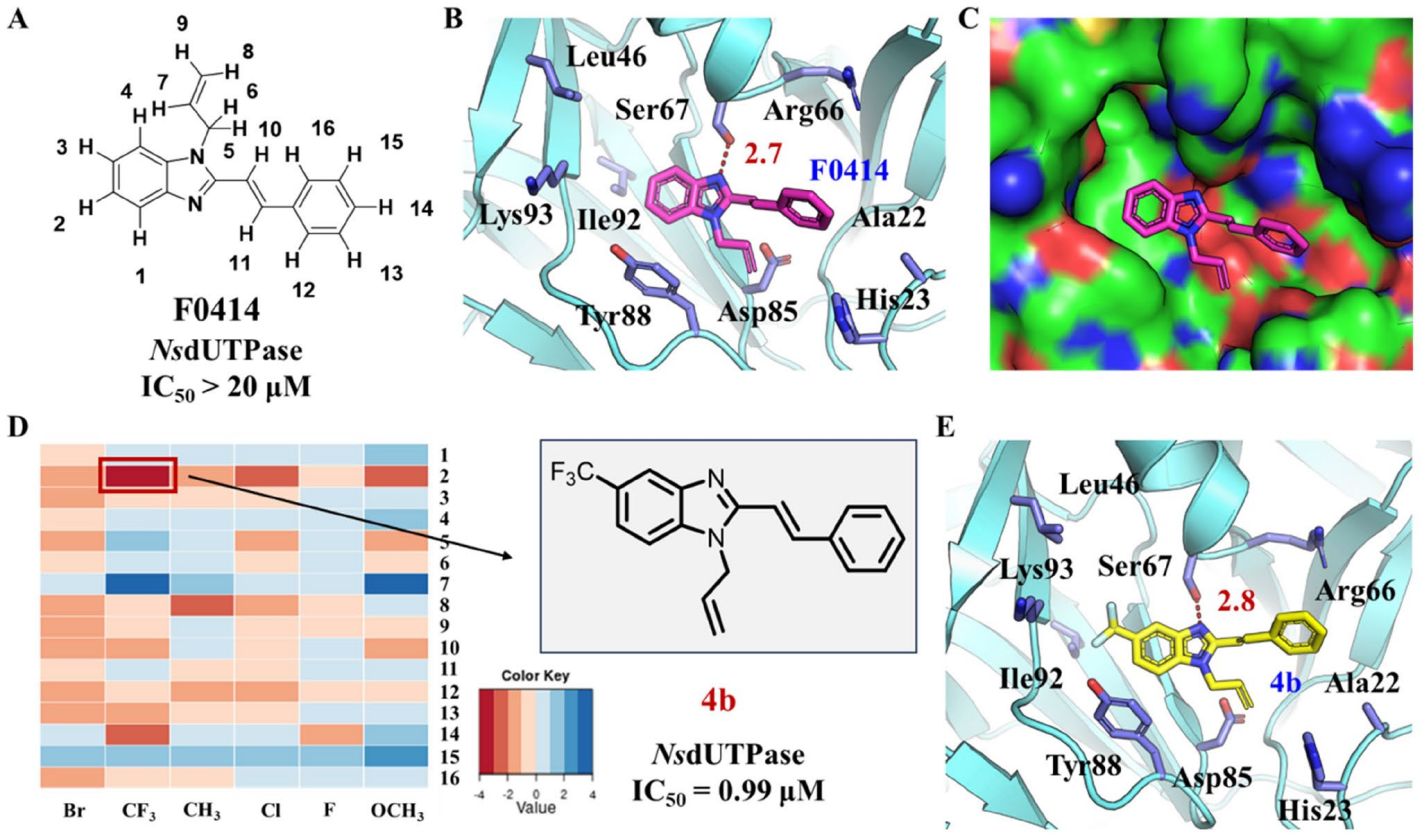
Substituent optimisation of compound **F0414**. (A) The structure of **F0414** and hydrogen number. It exhibited limited activity to *Ns*dUTPase. (B) The predicted binding mode between *Ns*dUTPase and **F0414**. (C) The hydrophobic surface of *Ns*dUTPase in complex with compound **F0414**. (D) The most potent compound **4b** showed the lowest binding free energy shift in substituent optimisation. (E) The predicted binding mode between *Ns*dUTPase and compound **4b**.

To verify the potential of *Ns*dUTPase as a target for anti-*Nocardia* drugs, a structure modification was performed to discover highly efficient *Ns*dUTPase inhibitors. The previously reported dUTPase inhibitors were usually uracil derivatives, which showed high cost and complicated synthesis steps. In 2021, Zhang *et al.* discovered that compound **F0414** could act as an antitubercular agent by targeting *Mt*dUTPase (Figure 2(A))^20^. It showed a promising inhibitory activity and low cost; therefore, it might be regarded as a promising starting structure for *Ns*dUTPase. But the *in vitro* experiment results showed that **F0414** did not exhibit sufficient inhibitory activity towards *Ns*dUTPase with an IC_50_ value higher than 20 μM (Table 2). Based on this situation, a structural modification was performed based on **F0414** to improve the activity towards *Ns*dUTPase.

**Table 2.** The structure and activity of compound **F0414** and **4a–4i** against *Ns*dUTPase.

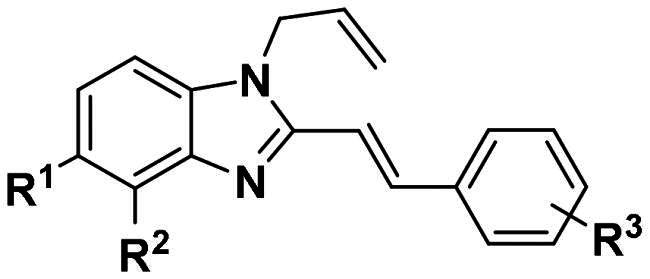				
	R^1^	R^2^	R^3^	IC_50_ (μM)
**4a**	H	H	*o*-OCH_3_	2.65
**4b**	–CF_3_	H	H	0.99
**4c**	–OCH_3_	H	H	3.78
**4d**	H	–CH_3_	H	>20
**4e**	–CN	H	H	>20
**4f**	–COOCH_3_	H	H	18.34
**4g**	H	H	*p*-N(CH3)_2_	2.98
**4h**	–COPh	H	H	9.62
**4i**	–COPh	H	*o*-OCH_3_	7.54
**F0414**	H	H	H	>20

In order to provide a useful guidance for structural optimisation, the binding mode between **F0414** and *Ns*dUTPase was predicted using molecular docking first. The result showed that it formed a hydrogen bond with the side chain of Ser67 with a distance of 2.8 Å (Figure 2(B)). In addition, its benzoimidazole group had hydrophobic interaction with surrounding residues, such as Leu46, Tye88, Ile92, and Lys93. While the styrene group was located in a hydrophobic subpocket consisted of Ala22, His23, Arg66, and Asp85. The binding mode between *Ns*dUTPase and **F0414** indicated that the hydrophobic interaction might play an important role to the binding. But we could find that there still were some spaces for ligands optimisation to enhance the binding with protein (Figure 2(C)). Therefore, adding hydrophobic substituents to enhance the interaction with *Ns*dUTPase might be a promising way to improve bioactivity.

**Scheme 1.**
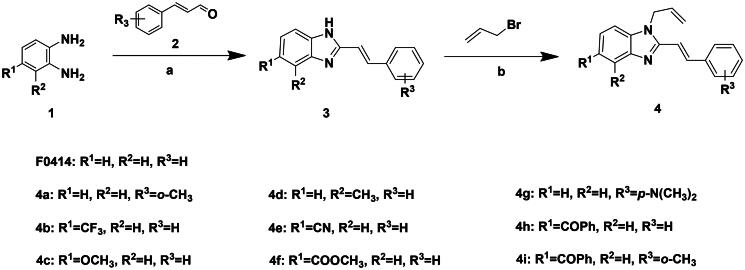
Reagents and conditions: (a) Na_2_S_2_O_5_, DMF, **12h**, 150 °C; (b) toluene, potassium tert-butoxide, **12h**, 140 °C.

Based on the analysis above, we tried to added a series of different hydrophobic substituents to the possible replacement site of **F0414**. These substituents contained –CH_3_, –CF_3_, –OCH_3_, –F, –Cl, and –Br^22^. The calculation results indicated that adding these substituents in some site might increase the binding affinity (Figure 2(D), Table S1). And the binding mode indicated that the most potent compound **4b** formed stronger hydrophobic interactions with surrounding residues, like Tye88, Ile92, and Lys93 (Figure 2(E)). Finally, a series of compounds were synthesised, and the general synthetic route of target compounds is depicted in Scheme 1. The *o*-phenylenediamine or derivatives **1** were reacted with cinnamaldehyde or derivatives **2** with sodium metabisulfite as catalyst to obtain compound **3**. The compound **3** reacted with 3-bromoprop-1-ene to obtain the corresponding product compound **F0414** and **4a–4i**.

The *in vitro* enzyme inhibitory activity of synthesised compounds was evaluated. It was satisfied to see that compounds with hydrophobic substituents showed an obvious activity improvement compared with **F0414**, while compounds with polar substituents still exhibited limited inhibitory activity to *Ns*dUTPase (Table 2). Among these compounds, compound **4b** exhibited the best bioactivity with the IC_50_ value of 0.99 μM. In addition, compounds **4a**, **4g**, and **4c** with hydrophobic substituents showed improved activity against *Ns*dUTPase, which indicated that our predicted binding mode was reliable. But substituents in some improper site also led to the decrease of bioactivity. For example, compounds **4d** and **4e** exhibited IC_50_ values of higher than 20 μM. The bioassay results indicated that our computational design was reliable, and compound **4b** might act as a lead compound.

### Structure-guided optimisation to discover more potent scaffold

**Figure 3.**
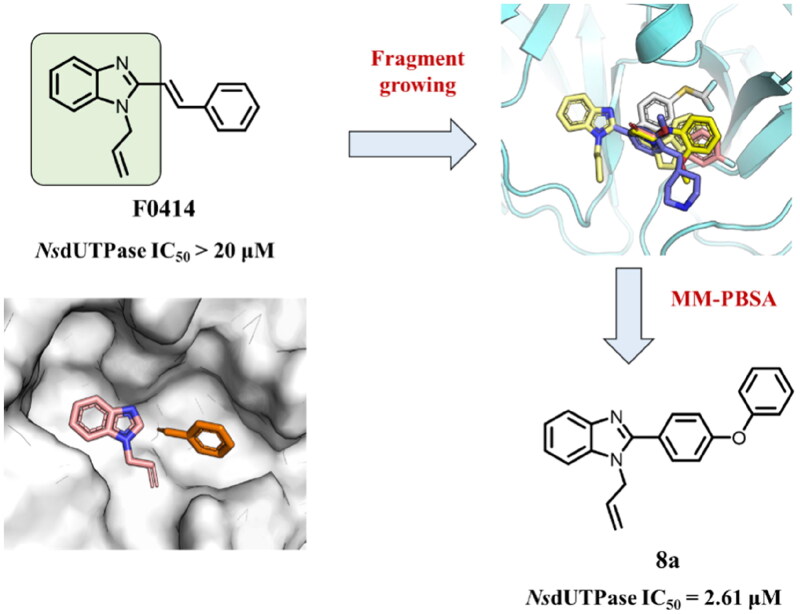
The computational discovery of more potent *Ns*dUTPase inhibitor scaffold via fragment growing. The ACFIS 2.0 web server was employed.

To find more potent scaffold and improve the hydrophobic interaction between inhibitors and *Ns*dUTPase, a scaffold optimisation was performed by using ACFIS 2.0 web server (Figure 3)^23^. The binding mode analysis indicated that benzoimidazole group formed a hydrogen bond and hydrophobic interaction with surrounding residues; therefore, it was regarded as the core fragment for *Ns*dUTPase activity and maintained for fragment growing. Then, a series of fragments from FDA-approved drugs were linked into core fragment to generate new compounds, and their binding affinity with *Ns*dUTPase was predicted using MM-PBSA method^24^.

**Scheme 2.**
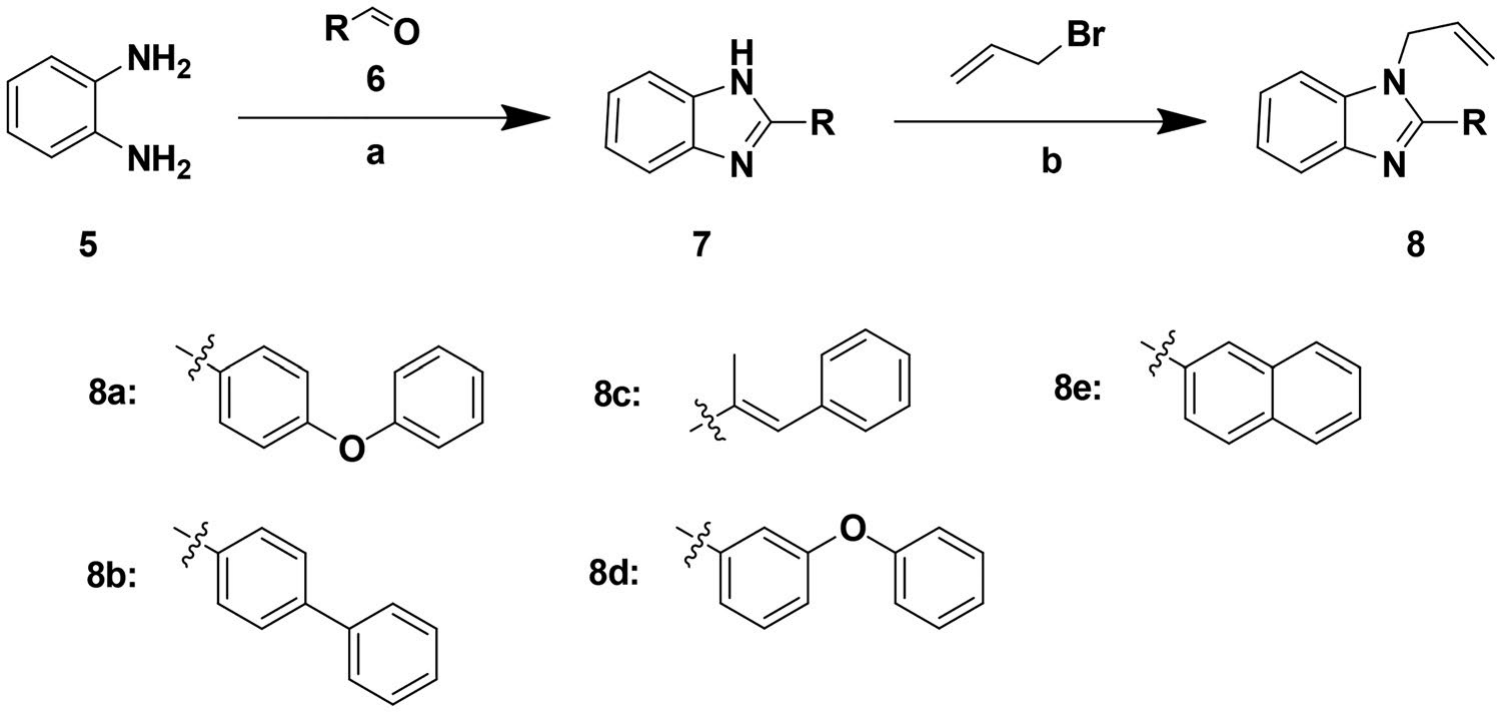
Reagents and conditions: (a) Na_2_S_2_O_5_, DMF, **12h**, 150 °C; (b) toluene, *t*-BuOK, **12h**, 140 °C.

The top-five fragments with the lowest binding free energy are shown in Table 3. It was noticed that compound with diphenyl ether group showed a lowest binding free energy. Besides, other compounds with hydrophobic groups also exhibited high binding affinity. Therefore, these compounds were synthesised to explore their bioactivity to *Ns*dUTPase (Scheme 2). They were synthesised according to the following steps. The *o*-phenylenediamine **5** was reacted with cinnamaldehyde or derivatives **6** with sodium metabisulfite as catalyst to obtain compound **7**. Compound **7** reacted with 3-bromoprop-1-ene to obtain the corresponding product compounds **8a–8e**.

**Table 3.** The structures, binding free energy (kcal/mol) and activity of **8a–8e** towards *Ns*dUTPase.

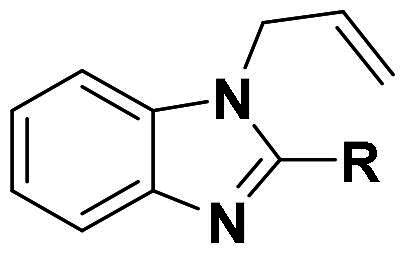							
	R	Δ*E*_vdw_	Δ*E_ele_*	Δ*E*_PB_	Δ*E*_SA_	Δ*G*	IC_50_ (μM)
**8a**	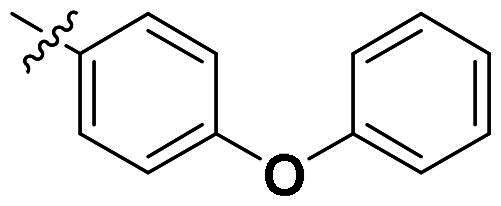	−23.68	−15.21	27.31	−6.85	−18.37	2.61
**8b**	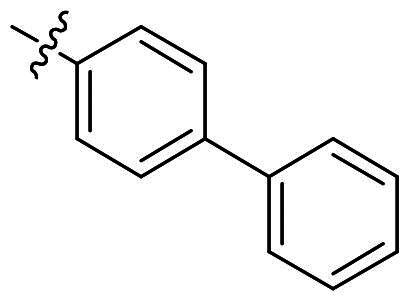	−19.50	−18.11	29.05	−6.94	−15.51	>20
**8c**	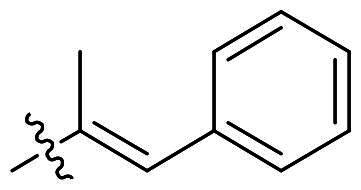	−20.73	−12.47	25.18	−5.50	−13.52	>20
**8d**	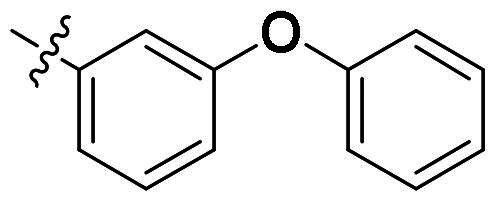	−18.63	−11.52	24.43	−5.47	−11.19	15.68
**8e**	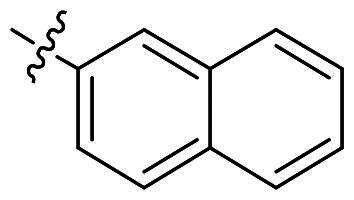	−17.84	−10.80	23.23	−5.60	−11.01	10.51

The bioactivity of compounds **8a–8e** was further evaluated. The results showed that compound **8a** with diphenyl ether group showed a promising inhibitory activity (IC_50_ = 2.61 μM). This result indicated that our predicted results were suitable for the prediction of dUTPase inhibitory activity, and the diphenyl ether group was also a commonly used structure for drugs and pesticides. The diphenyl ether group might form stronger hydrophobic interaction with surrounding residues compared with **F0414**, which might be the reason for its better activity. Also, compounds **8b**, **8d**, and **8e** also showed better activity than that of **F0414**, which suggest the hydrophobic interaction was important to the bioactivity. But from the result, it could be noticed that the shape of fragments was important to the activity, and improper shape would cause steric clash and reduce the activity. For example, modifying the linking position of diphenyl ether group would lead to the activity loss. In addition, the biphenyl group might form steric clash with the binding pocket of dUTPase and even cause activity loss. The fragment growing result indicated that diphenyl ether group might be a more potent substructure for dUTPase inhibitory activity, and the substituents would be performed based on this structure.

### Substituent optimisation of compound 8a

**Scheme 3.**
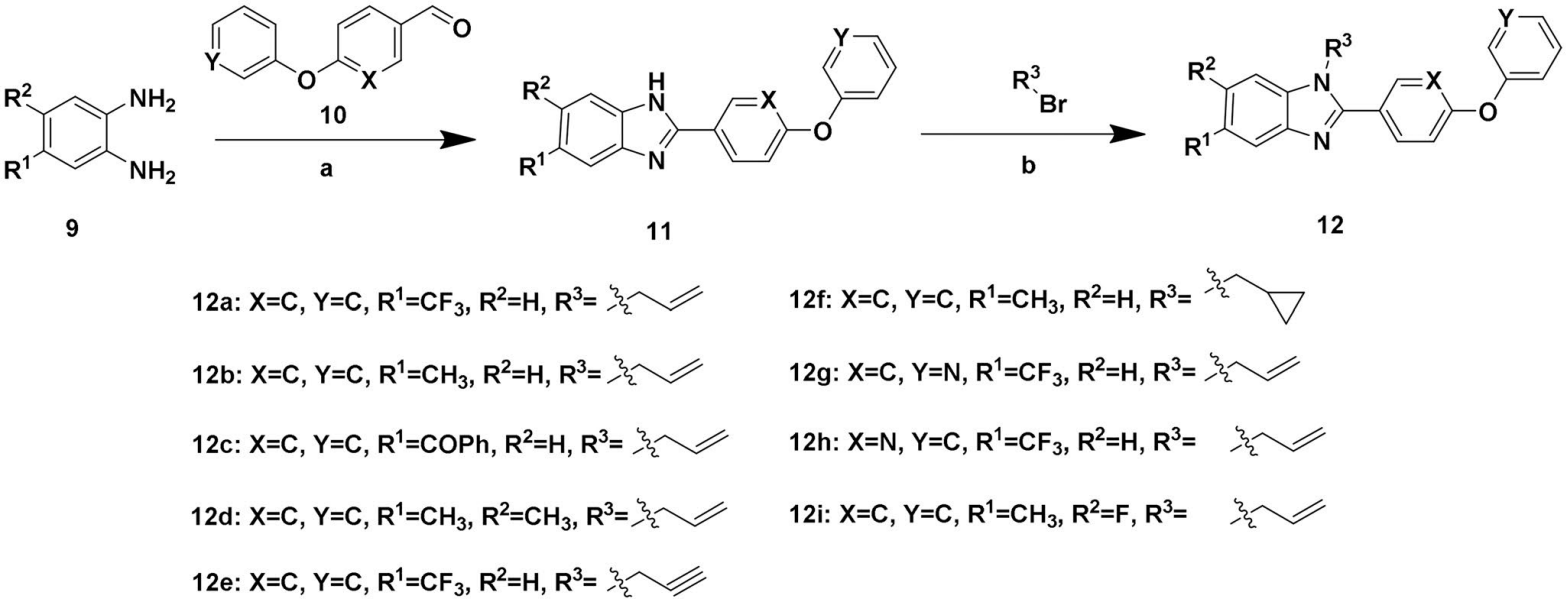
Reagents and conditions: (a) Na_2_S_2_O_5_, DMF, **12h**, 150 °C; (b) toluene, *t*-BuOK, **12h**, 140 °C.

To further improve the bioactivity of compound **8a**, a series of modification was performed. The hydrophobic substituents were added and some modifications were performed on the compound **8a**. Based on this, a series of derivatives were synthesised (Scheme 3). The synthesis was performed according to the following steps. The *o*-phenylenediamine and derivatives **9** were reacted with 4-phenoxybenzaldehyde or derivatives **10** with sodium metabisulfite as catalyst to obtain compound **11**. Compound **11** reacted with brominated alkyl group to obtain the corresponding product compounds **12a–12i**.

The *in vitro* enzyme activity assay results showed that replacing styrene group with more hydrophobic group can improve the activity. The most potent compound **12b** exhibited an IC_50_ value of 0.7 μM, which was much better than that of **F0414**. But adding hydrophobic groups may not always lead to an activity improvement. For example, the bulky group in R^1^ leads to an activity decrease and compound **12c** exhibited an IC_50_ value of 15.35 μM. In addition, different substituents were explored in R^3^ group, and propargyl (**12e**) and cyclopropane (**12f**) groups showed worse activity than that of allyl group. In addition, the nitrogen atom reduced the electron density of diphenyl ether, which might reduce the cation–π interaction between ligands and protein therefore decreases the activity (compound **12g** and **12h**). The promising activity of synthesised compounds made us hope to explore its anti-*Nocardia* activity (Table 4).

**Table 4.** The activity of **12a–12i** (kcal/mol) against *Ns*dUTPase.

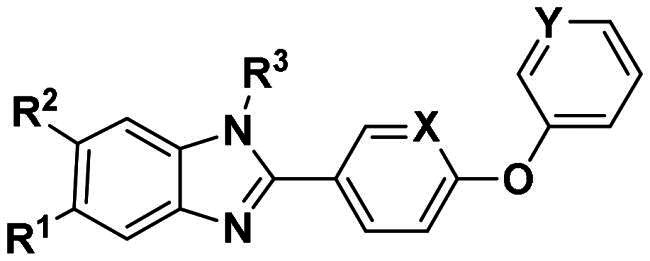						
	R^1^	R^2^	R^3^	X	Y	IC_50_ (μM)
**12a**	–CF_3_	H	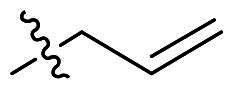	C	C	8.36
**12b**	–CH_3_	H	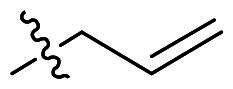	C	C	0.7
**12c**	–COPh	H	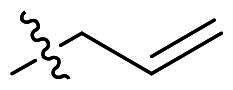	C	C	15.35
**12d**	–CH_3_	–CH_3_	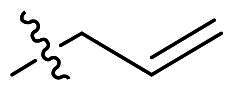	C	C	3.71
**12e**	–CF_3_	H	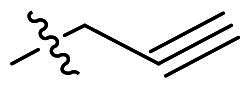	C	C	14.77
**12f**	–CH_3_	H	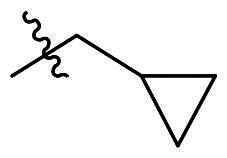	C	C	>20
**12g**	–CF_3_	H	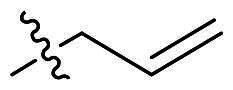	C	N	>20
**12h**	–CF_3_	H	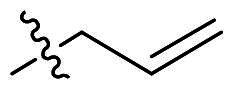	N	C	>20
**12i**	–CH_3_	F	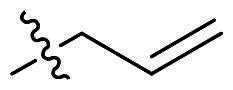	C	C	7.84

**Figure 4.**
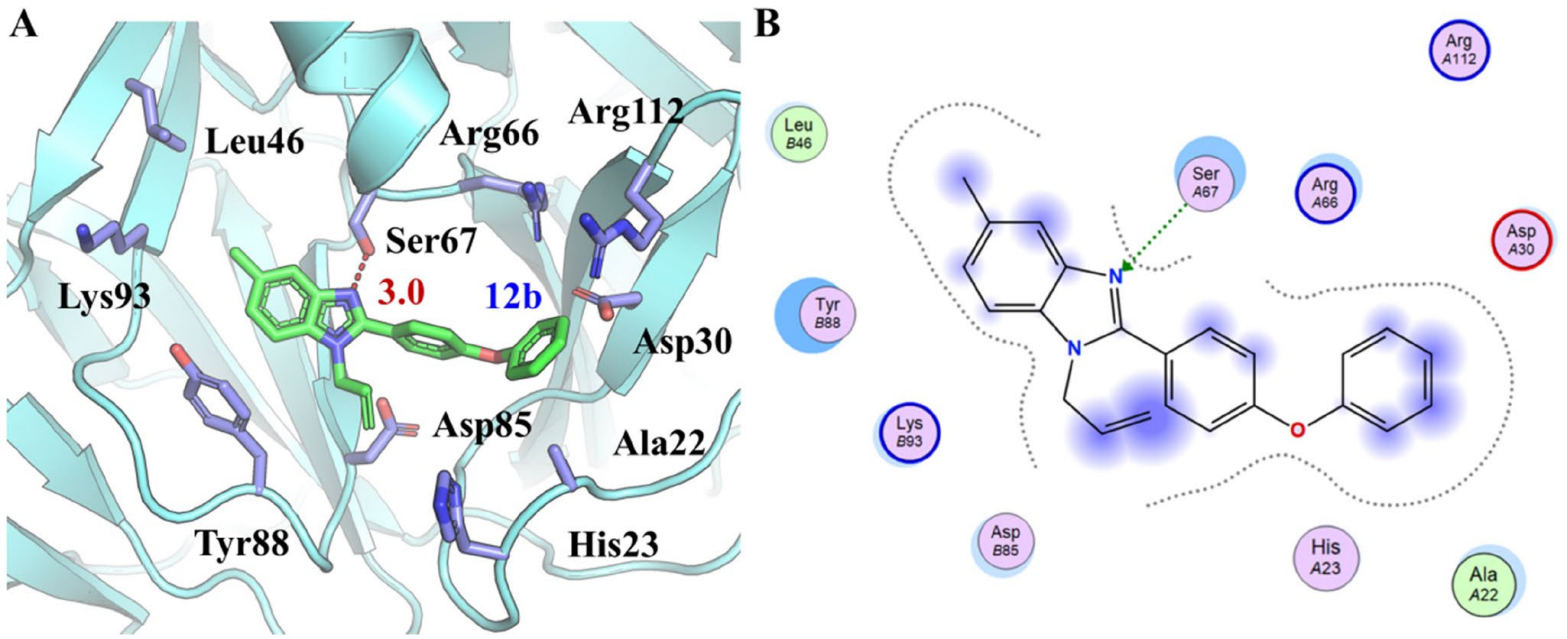
The predicted binding mode between **12b** and *Ns*dUTPase using molecular docking. (A) The cartoon and stick model of protein–ligand interactions. The hydrogen bonds are shown in red dotted lines. (B) The 2-D protein–ligand interaction.

The binding mode between **12b** and *Ns*dUTPase was predicted using molecular docking and the SAR analysis was therefore performed (Figure 4). It was noticed that it formed a hydrogen bond with the side chain of Ser67. In addition, the benzoimidazole group was located in a hydrophobic subpocket consisting of Leu46, Lys93, Tyr88, and Asp85. The extra –CH_3_ or –CF_3_ might increase activity could prove this binding mode compared with compound **8a**. But too much substituent might cause the steric clash and lead to an activity decrease such as compound **12c**. Besides, its diphenyl ether group formed hydrophobic interaction with Arg66, Arg112, Asp30, Ala22, and His23. But replacing carbon atom with nitrogen atom in the improper position of diphenyl ether group like compounds **12g** and **12h**, it might form electrostatic clashes with Asp85 or Asp30 to reduce activity. For the allyl group, it showed hydrophobic interaction with Tyr88, His23, and Asp85. But for compounds **12e** and **12f**, they were replaced by less flexible or bulkier substituents, which might lead to a steric clash with surrounding residues, such as His23 and lead to the activity decrease. The hydrogen bonds and strong hydrophobic interaction between **12b** and *Ns*dUTPase might be the key reason for the promising activity of compound **12b**.

### Anti-*Nocardia* activity of synthesised compounds

For the essential role of *Ns*dUTPase in *Nocardia* strains, compounds that inhibit dUTPase activity might prevent the growth of *N. seriolae* and *N. salmonicida*. Therefore, the anti-*Nocardia* activity of compound **F0414** and synthesised compounds was determined by microplate alamar blue assay (MABA) method (Table 5). It was noticed that compounds **4b** and **12b**, which exhibited the best *Ns*dUTPase inhibitory activity, showed the most promising anti-*Nocardia* activity. These results indicated that the *Ns*dUTPase might be a potent target to anti-*Nocardia*.

**Table 5.** Anti-*Nocardia* activity of synthesised compounds.

	MIC (mg/L)	
	*N. seriolae*	*N. salmonicida*
**F0414**	8	2
**4a**	4	2
**4b**	1	0.5
**4c**	>50	16
**4d**	16	16
**4e**	>50	16
**4f**	>50	32
**4g**	>50	16
**4h**	8	8
**4i**	8	4
**8a**	4	4
**8b**	4	4
**8c**	8	4
**8d**	>50	32
**8e**	4	4
**12a**	>50	16
**12b**	2	1
**12c**	16	8
**12d**	8	4
**12e**	32	16
**12f**	16	8
**12g**	>50	32
**12h**	>50	32
**12i**	4	4
Oxytetracycline	1	4
Florfenicol	4	16
Sulfadimethoxine	>50	12.5
Sulfamerazin	>50	>50

For *N. seriolae*, the MIC values of **4b** and **12b** were 1 and 2 mg/L, respectively, which are much lower than that of **F0414**. In addition, the approved drug sulfadimethoxine and sulfamerazin showed serious resistance to *N. seriolae* (MIC > 50 mg/L). Although oxytetracycline (MIC = 1 mg/L) and florfenicol (MIC = 4 mg/L) showed inhibitory activity to *N. seriolae*, the activity of compound **4b** was better or equal to them. For *N. salmonicida*, two compounds also showed lower MIC values (MIC = 0.5 and 1 mg/L) than **F0414**. More importantly, all the four approved drugs showed higher MIC values (ranged from 4 to more than 50 mg/L) than that of compound **4b** and **12b**. All the results indicated that two compounds possessed a potent anti-*Nocardia* activity than approved drugs, both for *N. seriolae* and *N. salmonicida*, which indicated that they have the potent to become new class of anti-*Nocardia* candidate.

### Cytotoxicity of compounds 4b and 12b

**Figure 5.**
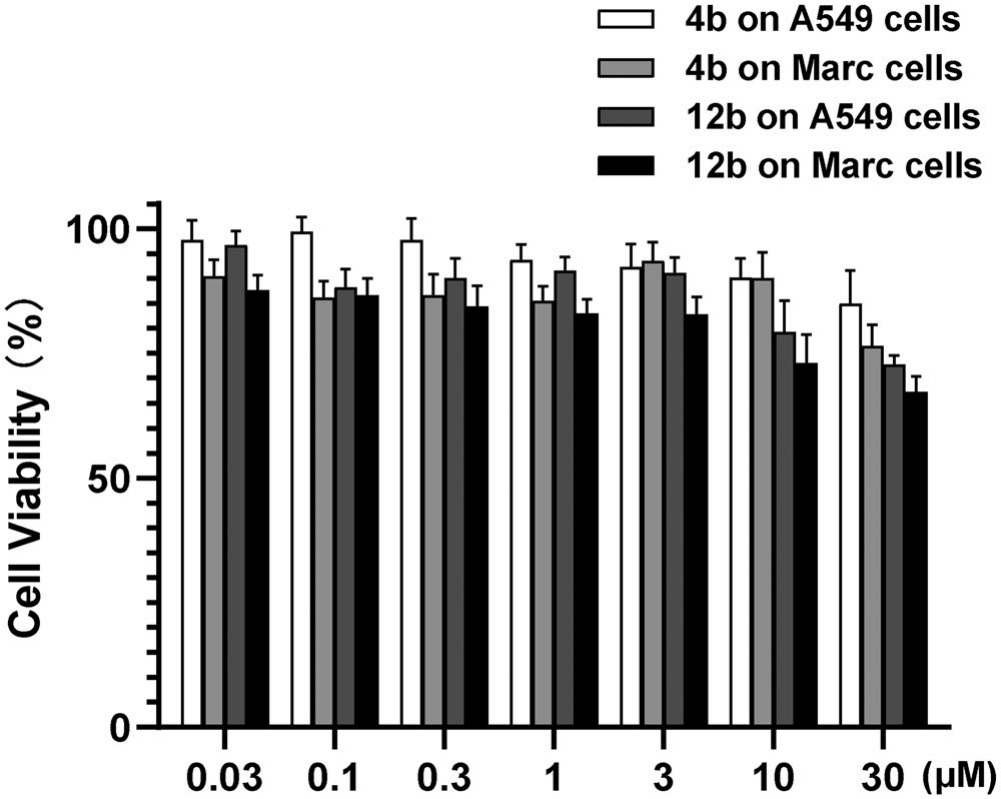
Cytotoxicity of **4b** and **12b** measured by CCK-8 assay. A549 and Mark cells were treated with **4b** and **12b** respectively from 0.03 to 30 µM.

The cytotoxicity of **4b** and **12b** was performed on A549 and Marc cells. The cell viability values were higher than 70% when the concentration of **4b** and **12b** reached higher than 30 µM (about 10.0 mg/L), which were significantly higher than the MICs of its anti-*Nocardia* activity (Figure 5). The results suggested a comparable selectivity of **4b** and **12b** to pathogens and hosts, which showed lower MIC values to *N. seriolae* and *N. salmonicida* as well as lower cytotoxicity to mammal cells.

### Molecular mechanism of compounds 4b and 12b binding with NsdUTPase

**Figure 6.**
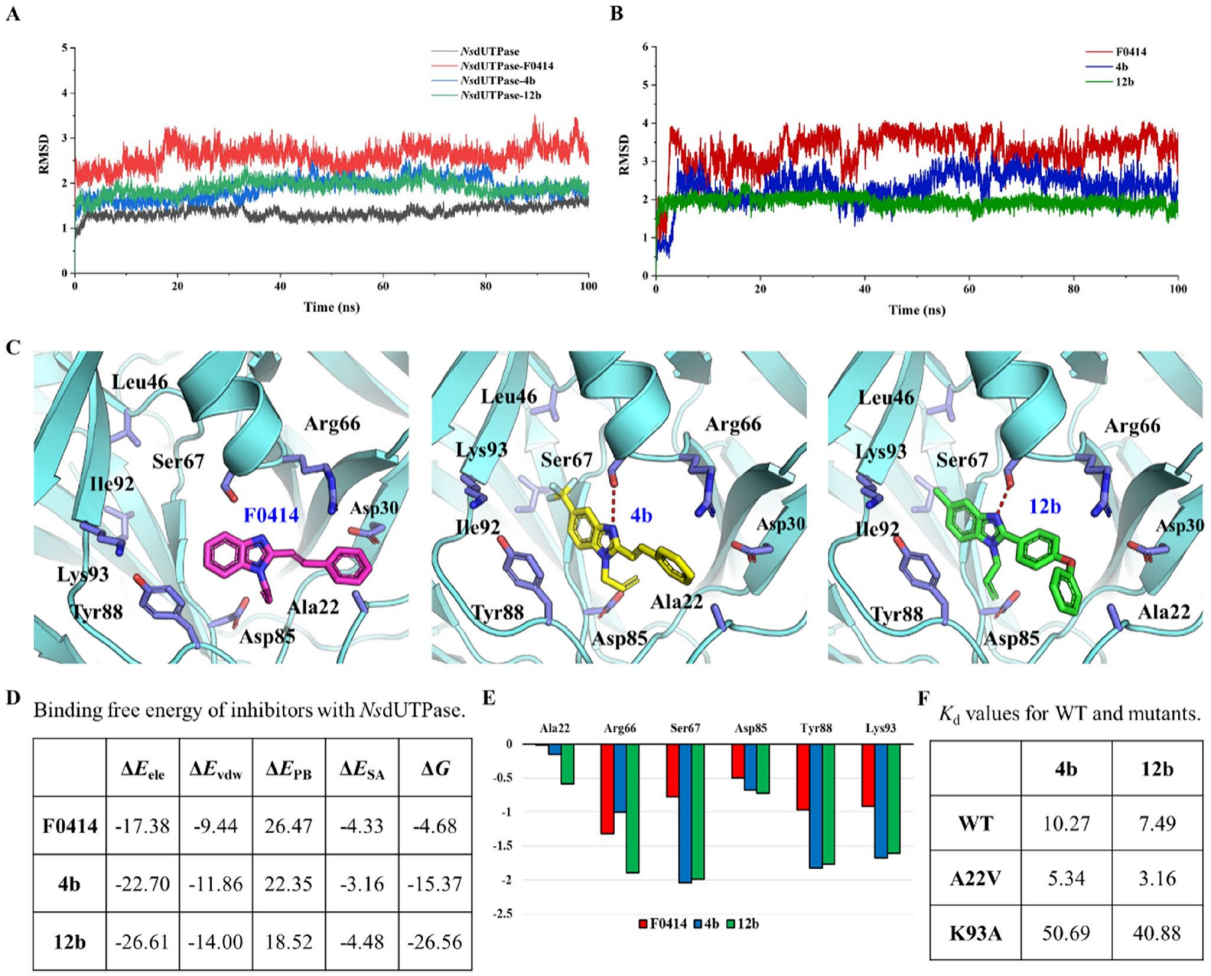
Molecular mechanism of compound **4b** and **12b**. (A) The RMSD value of different protein–ligand complex during MD simulations. (B) The RMSD value of different ligands during MD simulations. (C) The binding mode between protein and ligands after MD simulations. The hydrogen bonds are shown in red dotted lines. (D) The binding free energy between protein and ligands (kcal/mol). (E) The binding free energy decomposition results (kcal/mol). (F) The *K*_d_ values of compound **4b** and **12b** to WT and mutants of *Ns*dUTPase.

To explain the potent molecular mechanism that compound **4b** and **12b** exhibited promising activity against *Ns*dUTPase, the molecular dynamics (MD) simulations and binding free energy calculation were performed. It was noticed that compound **12b** showed the lowest RMSD values at around 1.8 Å among three complexes, which indicated that it was bind most tightly to *Ns*dUTPase during MD simulations (Figure 6(A)). And the RMSD value was lower than 2 Å, which suggested that our predicted binding mode was reliable. But the RMSD value of ligand was exhibited some fluctuation, especially for compound **F0414** (Figure 6(B)). The stable protein–ligand complex indicated that our predicted binding modes were reliable, but the reasons for their different bioactivity might need to be further analysed.

The binding modes of studied compounds with *Ns*dUTPase were compared to explain the reason that they exhibited different binding affinity. It was noticed that compounds **4b** and **12b** shared a similar binding mode (Figure 6(C)). They formed conserved hydrogen bonds with the side chain of Ser67. And the main differences for binding affinity are the hydrophobic interactions. Compared with compound **F0414**, compound **4b** contained a –CF_3_ group, which enhanced the hydrophobic interaction with surrounding residues compared with compound **F0414**. Moreover, for compound **12b**, the styrene group was replaced by the diphenyl ether group, which obviously strengthen the binding free energy and might be reason for its better bioactivity. But for compound **F0414**, the weak interaction with protein made it unstable in the binding pocket, and the hydrogen bond might be destroyed, which might explain its decreased activity.

The binding free energy between ligands and protein was calculated using MM-PBSA approach. We could find that compound **12b** exhibited the lowest binding free energy (−26.56 kcal/mol) compared with **F0414** (−4.68 kcal/mol) and **4b** (−15.37 kcal/mol), which coincides with our enzyme activity assay (Figure 6(D)). In addition, the electrostatic and van der Waals energy were −26.61 kcal/mol and −14.00 kcal/mol, respectively, which indicated that the hydrophobic interaction might be the main contribution to the binding. To verify the key interaction between compound **12b** and *Ns*dUTPase, the binding free energy was decomposed into each residue. It was noticed that Arg66, Ser67, Tyr88, and Lys93 exhibited the largest contribution to the binding of compound **12b** (Figure 6(E)). While for compound **4b** and **F0414**, these residues exhibited higher binding free energy contribution. In addition, the mutants of A22V and K93A coincided with our predicted binding modes. The A22V would increase the hydrophobic interaction of **4b** and **12b**, which lead to lower *K*_d_ values. While K93A would decrease the binding of **4b** and **12b**, which lead to higher *K*_d_ values. The binding mode analysis combined with binding free energy calculation suggested that hydrophobic interaction was the main contribution to the activity of compound **16**, and Arg66 and Ser67 were the key residues.

## Conclusions

*Nocardia* is an important pathogenic bacterium to human, animal husbandry, and aquaculture. The efficient drug to combat *Nocardia* is a main goal for scientists. In this study, we revealed that *Ns*dUTPase inhibitors could act as promising inhibitor to repress *Nocardia*. The first crystal structure of *Ns*dUTPase was released with a resolution of 2.12 Å. It was noticed that *Ns*dUTPase shared a similar conformation with *Mt*dUTPase. But the differences of key residues in the active site cased a *Mt*dUTPase inhibitor **F0414** showed limited activity to *Ns*dUTPase. Based on this, fragment growing and substituent optimisation were performed. The most potent compound **4b** and **12b** showed IC_50_ values of 0.99 μM and 0.70 μM, respectively, which exhibited more than 20-fold activity improvement. More importantly, compound **4b** and **12b** showed satisfied anti-*Nocardia* activity *in vitro*, which was even better than approved antibiotic oxytetracycline and florfenicol. And they showed low cytotoxicity to human A549 and Marc cells. Molecular modelling study indicated that hydrophobic interaction was the main contribution for the binding of inhibitors, and Arg66 and Ser67 were the key residues. Our results suggested that compounds **4b** and **12b** could be regarded as promising anti-*Nocardia* agents.

## Materials and methods

### Protein expression and purification

Full length of *Ns*dUTPase (WP_033087955.1) was expressed in *E. coli* BL21(DE3) cells and purified through a Ni-NTA affinity column (Sangon Biotech, Shanghai, China). The protein elution was further purified with a Superdex 200 Increase 10/300 column (Cytiva, Uppsala, Sweden) equilibrated with SEC buffer (20 mM Tris pH 8.0, 300 mM NaCl, 1 mM TCEP). Peak fractions containing the target proteins were pooled and concentrated.

### Crystallisation, data collection, and structure determination

Concentrated *Ns*dUTPase (10 mg/mL) protein was incubated with 2 mM dUTP and 1 mM MgCl_2_ at 20 °C for 30 min before setting up sitting-drop crystallisation trials at 20 °C. Diffractable crystals of *Ns*dUTPase were obtained in 25% (w/v) polyethylene glycol 3350, 0.1 M Tris–HCl pH 8.5 at 20 °C. Crystals grew to approximately 0.1 mm × 0.1 mm × 0.1 mm in approximately three weeks. Crystallisation solution supplemented with 25% (w/v) PEG3350 was used as a cryoprotectant, and crystals were mounted on a nylon loop (Hampton Research, Aliso Viejo, CA) and flash-cooled in liquid nitrogen for data collection. The X-ray diffraction data were collected at TPS 07A, National Synchrotron Radiation Research Center (Hsinchu, Taiwan), and indexed, integrated, and scaled with programs XDS^25^. The crystals apparently belonged to space group P4_1_32 with a single molecule in the asymmetric unit. The *Ns*dUTPase–dUMP complex structures were solved by the molecular replacement method using Phaser from the CCP4 program suite^26^, with the structure of *M. tuberculosis* dUTPase in complex with dUPNPP (PDB 1SIX) as the search model^27^. Refinement and manual model building were performed using REFMAC5 and COOT^28^^,^^29^, respectively. Further refinement was performed and confirmed using Phenix^30^. Final statistics for data collection and structure refinement are represented in Table 1. Protein structures were illustrated using the program PyMOL. The atomic coordinates have been deposited in the Protein Data Bank under accession code 8XHM.

### Molecular docking

All the molecular docking study was performed using Autodock vina^31^. The structure of receptor protein *Ns*dUTPase was released in this work, and was prepared by removing water atoms, adding hydrogen atoms, and repairing side chains. The protonation states of protein and ligand were predicted by ProPKA 3.0^32^. Then, the ligands were docked into the docking box which was determined by the dUTP binding pocket. The Lamarckian genetic algorithm was employed for the docking and 20 poses were exported for the further analysis. And the binding modes between protein and ligands were analysed in PyMol.

### Computational scaffold optimisation

The computational scaffold optimisation was performed using ACFIS 2.0 web server. Fragments from FDA-approved drugs were selected as fragment library^33^. The complex of *Ns*dUTPase and core fragment was used as input file. Multiple conformations of *Ns*dUTPase were explored. Fragments from FDA approved drugs were utilised as fragment library, and were added to core fragment in different protein conformations to generate protein–ligand complexes. The binding free energy between protein conformations and ligand were calculated using MM-PBSA approach, and the lowest value was regarded as the binding free energy. Top 20 ranked ligands with the lowest binding free energy were output for the further analysis. After calculation, the physicochemical properties, drug-like property and synthetic accessibility were also evaluated.

### Chemistry

Unless otherwise specified, all common solvents and reagents were obtained from commercial sources and used without purification. All the synthesised products were confirmed by ^1^H and ^13^C nuclear magnetic resonance (NMR).^1^H NMR and ^13^C NMR spectra were recorded on a Bruker Avance 400 and III HD 600 spectrometer (Billerica, MA) in CDCl_3_ or DMSO-*d*_6_, and chemical shifts were recorded in parts per million (ppm) with trimethylsilane (TMS) as the internal standard. Dissolve *o*-phenylenediamine and derivatives in DMF, stir until dissolved, and add corresponding aldehyde dropwise and stir. Next, add catalyst sodium metabisulfite program temperature to 150 °C, reflux reaction for 12 h. After completing the TLC monitoring reaction, ethyl acetate was added to the reaction solution, washed saturated salt water, and anhydrous sodium sulphate. The solvent was removed by rotary evaporation to obtain compound with a yield of 90.0%. Dissolve obtained compound, add potassium tert butanol, and react with propylene oxide at 140 °C for 12 h. After reaction is complete, add ethyl acetate and wash saturated salt water. The organic phase is dried with anhydrous sodium sulphate, and the solvent is removed by rotary evaporation. After column chromatography separation, the final product is obtained with a yield of 80.0%

#### (E)-1-Allyl-2-styryl-1H-benzo[d]imidazole (F0414)

The *o*-phenylenediamine (108 mg, 1 mmol) was reacted with cinnamaldehyde (264 mg, 2 mmol) with sodium metabisulfite as catalyst to obtain compound **3**. The compound **3** (220 mg, 1 mmol) reacted with 3-bromoprop-1-ene (240 mg, 2 mmol) to obtain the corresponding product compound **F0414**, yield of 81%.^1^H NMR (400 MHz, DMSO) *δ* 7.90 (d, *J* = 15.8 Hz, 1H), 7.86–7.78 (m, 2H), 7.71–7.61 (m, 1H), 7.59–7.51 (m, 1H), 7.51–7.43 (m, 3H), 7.43–7.35 (m, 1H), 7.31–7.18 (m, 2H), 6.06 (ddt, *J* = 17.1, 10.2, 5.1 Hz, 1H), 5.25–5.15 (m, 1H), 5.15 (dd, *J* = 4.9, 2.0 Hz, 2H), 4.99 (dt, *J* = 17.1, 1.6 Hz, 1H). ^13^C NMR (101 MHz, DMSO) *δ* 151.11, 143.24, 136.57, 136.33, 135.74, 134.19, 129.47, 129.32, 127.93, 127.63, 126.44, 122.73, 122.69, 119.05, 116.90, 114.61, 110.83, 45.19. HR-MS (EI) *m/z* calcd 260.3400; found, 261.3405 [M + H]^+^.

#### (E)-1-Allyl-2-styryl-1H-benzo[d]imidazole (4a)

In a similar procedure as described for compound **F0414**, yield of 74%. ^1^H NMR (400 MHz, CDCl_3_) *δ* 8.24 (d, *J* = 16.0 Hz, 1H), 7.85–7.77 (m, 1H), 7.58 (dd, *J* = 7.6, 1.7 Hz, 1H), 7.37–7.22 (m, 4H), 7.22 (d, *J* = 16.0 Hz, 1H), 7.04–6.90 (m, 2H), 6.02 (ddt, *J* = 17.1, 10.0, 4.9 Hz, 1H), 5.26 (dq, *J* = 10.3, 1.4 Hz, 1H), 5.14–5.03 (m, 1H), 4.88 (dt, *J* = 4.9, 1.8 Hz, 2H), 3.92 (s, 3H). ^13^C NMR (101 MHz, CDCl_3_) *δ* 158.04, 151.68, 143.31, 135.40, 133.17, 132.13, 130.12, 128.54, 125.05, 122.55, 122.47, 120.73, 119.38, 117.47, 114.24, 111.14, 109.36, 55.45, 45.58. HR-MS (EI) *m/z* calcd 326.3990; found, 327.3994 [M + H]^+^.

#### (E)-1-Allyl-2-styryl-5-(trifluoromethyl)-1H-benzo[d]imidazole (4b)

In a similar procedure as described for compound **F0414**, yield of 76%. ^1^H NMR (400 MHz, CDCl_3_) *δ* 8.04 (d, *J* = 15.7 Hz, 1H), 7.84 (d, *J* = 8.5 Hz, 1H), 7.66–7.47 (m, 4H), 7.47–7.31 (m, 3H), 7.03 (d, *J* = 15.9 Hz, 1H), 6.03 (ddt, *J* = 17.2, 10.4, 4.7 Hz, 1H), 5.30 (dt, *J* = 10.4, 1.8 Hz, 1H), 5.05 (dt, *J* = 17.1, 2.0 Hz, 1H), 4.92 (dt, *J* = 4.9, 1.9 Hz, 2H). ^13^C NMR (101 MHz, CDCl_3_) *δ* 152.35, 144.26, 137.92, 137.33, 134.47, 133.71, 133.53–120.65 (m), 117.66 (d, *J* = 171.7 Hz), 111.28, 106.08 (q, *J* = 4.3 Hz), 44.57. HR-MS (EI) *m/z* calcd 328.3382; found, 329.3391 [M + H]^+^.

#### (E)-1-Allyl-2-styryl-1H-benzo[d]imidazole (4c)

In a similar procedure as described for compound **F0414**, yield of 67%. ^1^H NMR (400 MHz, CDCl_3_) *δ* 7.91 (dd, *J* = 19.8, 15.8 Hz, 1H), 7.62–7.52 (m, 2H), 7.45–7.33 (m, 2H), 7.38–7.28 (m, 1H), 7.33–7.22 (m, 1H), 7.16 (d, *J* = 8.8 Hz, 1H), 7.03–6.85 (m, 2H), 5.99 (ddq, *J* = 17.1, 10.4, 4.6 Hz, 1H), 5.29–5.19 (m, 1H), 5.08–4.97 (m, 1H), 4.81 (ddt, *J* = 6.4, 4.6, 1.9 Hz, 2H), 3.86 (d, *J* = 4.6 Hz, 3H). ^13^C NMR (101 MHz, CDCl_3_) *δ* 156.74, 151.02, 143.95, 136.05, 132.09, 131.92, 129.01, 128.85, 127.24, 117.48, 113.21, 112.87, 111.94, 101.36, 55.79, 45.62. HR-MS (EI) *m/z* calcd 290.3660; found, 291.3665 [M + H]^+^.

#### (E)-1-Allyl-4-methyl-2-styryl-1H-benzo[d]imidazole (4d)

In a similar procedure as described for compound **F0414**, yield of 71%. ^1^H NMR (400 MHz, CDCl_3_) *δ* 7.95 (d, *J* = 15.9 Hz, 1H), 7.65–7.53 (m, 2H), 7.45–7.30 (m, 2H), 7.26 (s, 1H), 7.20–7.10 (m, 2H), 7.12–7.00 (m, 2H), 6.01 (ddt, *J* = 17.1, 10.4, 4.7 Hz, 1H), 5.35–5.18 (m, 1H), 5.17–4.95 (m, 1H), 4.86 (dt, *J* = 4.7, 1.9 Hz, 2H), 2.73 (s, 3H). ^13^C NMR (101 MHz, CDCl_3_) *δ* 150.26, 142.58, 137.10, 136.18, 135.14, 132.19, 129.53, 128.99, 128.88, 127.31, 123.12, 122.72, 117.35, 113.60, 107.07, 45.64, 16.96. HR-MS (EI) *m/z* calcd 274.3670; found, 275.3671 [M + H]^+^.

#### (E)-1-Allyl-2-styryl-1H-benzo[d]imidazole-5-carbonitrile (4e)

In a similar procedure as described for compound **F0414**, yield of 69%. ^1^H NMR (400 MHz, CDCl_3_) *δ* 8.30–7.88 (m, 1H), 7.82 (dd, *J* = 8.3, 0.6 Hz, 1H), 7.67–7.57 (m, 2H), 7.53 (ddd, *J* = 12.0, 8.4, 1.5 Hz, 1H), 7.46–7.32 (m, 3H), 7.02 (dd, *J* = 15.8, 6.8 Hz, 1H), 6.02 (dd, *J* = 17.1, 10.4 Hz, 1H), 5.32 (ddt, *J* = 10.5, 6.9, 1.8 Hz, 1H), 5.05 (ddt, *J* = 17.1, 9.8, 1.9 Hz, 1H), 4.91 (dt, *J* = 4.9, 1.9 Hz, 2H). ^13^C NMR (101 MHz, CDCl_3_) *δ* 154.29, 146.17, 142.80, 135.00, 131.22, 131.17, 129.83, 129.74, 129.01, 129.00, 127.60, 127.54, 120.15, 120.00, 119.98, 118.31, 118.16, 114.27, 112.00, 111.94, 105.18, 45.83. HR-MS (EI) *m/z* calcd 285.3500; found, 286.3504 [M + H]^+^.

#### (E)-1-Allyl-2-styryl-1H-benzo[d]imidazol-5-yl acetate (4f)

In a similar procedure as described for compound **F0414**, yield of 70%. ^1^H NMR (400 MHz, CDCl_3_) *δ* 8.44 (d, *J* = 8.4 Hz, 1H), 7.80–7.70 (m, 1H), 7.66–7.32 (m, 5H), 7.25–7.06 (m, 1H), 6.72 (d, *J* = 8.3 Hz, 1H), 4.66 (s, 1H), 3.88 (s, 2H), 1.27 (dd, *J* = 11.5, 1.6 Hz, 1H). ^13^C NMR (101 MHz, CDCl_3_) *δ* 167.46, 152.63, 139.34, 135.67, 134.97, 131.59, 131.52, 129.22, 128.93, 127.54, 127.47, 125.01, 121.52, 117.90, 112.29, 52.15, 45.69. HR-MS (EI) *m/z* calcd 318.3760; found, 319.3764 [M + H]^+^.

#### (E)-4-(2-(1-Allyl-1H-benzo[d]imidazol-2-yl)vinyl)-N,N-dimethylaniline (4g)

In a similar procedure as described for compound **F0414**, yield of 72%. ^1^H NMR (400 MHz, CDCl_3_) *δ* 7.90 (d, *J* = 15.7 Hz, 1H), 7.79–7.71 (m, 1H), 7.52–7.43 (m, 2H), 7.32–7.14 (m, 3H), 6.77 (dd, *J* = 15.7, 1.3 Hz, 1H), 6.72–6.61 (m, 2H), 6.05–5.89 (m, 1H), 5.25–5.16 (m, 1H), 5.07–4.96 (m, 1H), 4.79 (tt, *J* = 4.4, 2.0 Hz, 2H), 2.98 (d, *J* = 1.9 Hz, 6H). ^13^C NMR (101 MHz, CDCl_3_) *δ* 152.12, 151.01, 143.35, 137.85, 135.39, 132.17, 128.71, 124.09, 122.41, 122.06, 118.96, 117.36, 112.08, 109.19, 107.98, 45.43, 40.27. HR-MS (EI) *m/z* calcd 303.4090; found, 304.4096 [M + H]^+^.

#### (E)-(1-Allyl-2-styryl-1H-benzo[d]imidazol-5-yl)(phenyl)methanone (4h)

In a similar procedure as described for compound **F0414**, yield of 79%. ^1^H NMR (400 MHz, CDCl_3_) *δ* 8.20 (d, *J* = 1.5 Hz, 1H), 8.05 (dd, *J* = 23.5, 15.8 Hz, 1H), 7.97–7.71 (m, 4H), 7.65–7.54 (m, 3H), 7.54–7.48 (m, 2H), 7.48–7.41 (m, 2H), 7.41–7.25 (m, 3H), 7.05 (dd, *J* = 15.8, 10.0 Hz, 1H), 6.04 (dddd, *J* = 17.2, 10.4, 8.9, 4.2 Hz, 1H), 5.38–5.25 (m, 1H), 5.06 (dq, *J* = 17.1, 1.8 Hz, 1H), 4.93 (dt, *J* = 4.9, 1.9 Hz, 2H). ^13^C NMR (101 MHz, CDCl_3_) *δ* 196.56, 152.89, 142.33, 138.70, 135.70, 132.44, 130.07, 128.98, 128.95, 127.54, 124.88, 118.54, 117.94, 112.47, 112.13, 45.72. HR-MS (EI) *m/z* calcd 364.4480; found, 365.4488 [M + H]^+^.

#### (E)-(1-Allyl-2-(2-methoxystyryl)-1H-benzo[d]imidazol-5-yl)(phenyl)methanone (4i)

In a similar procedure as described for compound **F0414**, yield of 69%. ^1^H NMR (400 MHz, CDCl_3_) *δ* 8.41–8.01 (m, 2H), 7.87–7.70 (m, 3H), 7.54–7.38 (m, 4H), 7.31 (d, *J* = 8.5 Hz, 1H), 7.25 (ddd, *J* = 8.9, 7.5, 1.7 Hz, 1H), 7.20–7.05 (m, 1H), 6.97–6.71 (m, 2H), 5.95 (dddt, *J* = 17.9, 10.1, 7.9, 4.9 Hz, 1H), 5.23 (dd, *J* = 8.2, 2.2 Hz, 1H), 5.02 (dt, *J* = 17.1, 2.0 Hz, 1H), 4.84 (dt, *J* = 4.9, 1.8 Hz, 2H), 3.84 (s, 3H). ^13^C NMR (101 MHz, CDCl_3_) *δ* 195.71, 157.10, 152.63, 141.45, 137.44, 137.35, 133.36, 131.18, 130.88, 130.61, 129.44, 129.00, 127.66, 127.12, 123.68, 123.60, 122.05, 119.71, 116.86, 112.50, 110.12, 108.36, 54.42, 44.79. HR-MS (EI) *m/z* calcd 394.4740; found, 395.4748 [M + H]^+^.

#### 1-Allyl-2-(4-phenoxyphenyl)-1H-benzo[d]imidazole (8a)

The *o*-phenylenediamine **5** (108 mg, 1 mmol) was reacted with cinnamaldehyde or derivatives **6** (396 mg, 2 mmol) with sodium metabisulfite as catalyst to obtain compound **7**. Compound **7** (286 mg, 1 mmol) reacted with 3-bromoprop-1-ene (240 mg, 2 mmol) to obtain the corresponding product compound **8a**, yield of 72%. ^1^H NMR (400 MHz, CDCl_3_) *δ* 7.86–7.80 (m, 1H), 7.77–7.72 (m, 2H), 7.42–7.35 (m, 2H), 7.35–7.32 (m, 1H), 7.32–7.27 (m, 2H), 7.21–7.14 (m, 1H), 7.13–7.03 (m, 4H), 6.08 (dd, *J* = 17.2, 10.5 Hz, 1H), 5.32 (dq, *J* = 10.5, 1.6 Hz, 1H), 5.22–4.91 (m, 1H), 4.83 (dt, *J* = 4.1, 2.0 Hz, 2H). ^13^C NMR (101 MHz, CDCl_3_) *δ* 159.16, 156.27, 153.44, 143.04, 135.98, 132.40, 130.83, 129.98, 124.63, 124.10, 122.84, 122.59, 119.82, 119.69, 118.38, 117.49, 110.30, 47.19. HR-MS (EI) *m/z* calcd 326.3990; found, 327.3995 [M + H]^+^.

#### 2-([1,1′-Biphenyl]-4-yl)-1-allyl-1H-benzo[d]imidazole (8b)

In a similar procedure as described for compound **8a**, yield of 85%. ^1^H NMR (400 MHz, CDCl_3_) *δ* 7.95–7.84 (m, 1H), 7.90–7.78 (m, 2H), 7.68 (dd, *J* = 8.4, 1.9 Hz, 2H), 7.67–7.55 (m, 2H), 7.47–7.35 (m, 2H), 7.37–7.17 (m, 4H), 6.01 (dddd, *J* = 18.6, 10.2, 4.4, 2.2 Hz, 1H), 5.31–5.21 (m, 1H), 5.09–4.97 (m, 1H), 4.75 (dp, *J* = 5.9, 1.9 Hz, 2H). ^13^C NMR (101 MHz, CDCl_3_) *δ* 153.38, 142.88, 142.49, 139.97, 135.91, 132.28, 129.53, 128.85, 127.79, 127.25, 127.02, 122.90, 122.59, 119.74, 117.37, 110.34, 47.08. HR-MS (EI) *m/z* calcd 310.4000; found, 311.4012 [M + H]^+^.

#### (E)-1-Allyl-2-(1-phenylprop-1-en-2-yl)-1H-benzo[d]imidazole (8c)

In a similar procedure as described for compound **8a**, yield of 80%. ^1^H NMR (400 MHz, CDCl_3_) *δ* 7.86–7.76 (m, 1H), 7.46–7.37 (m, 4H), 7.37–7.21 (m, 4H), 6.91 (q, *J* = 1.5 Hz, 1H), 6.06 (ddt, *J* = 17.2, 10.5, 4.5 Hz, 1H), 5.31 (ddt, *J* = 10.5, 1.8, 1.0 Hz, 1H), 5.09 (dtd, *J* = 17.2, 2.0, 0.9 Hz, 1H), 4.89 (dt, *J* = 4.1, 1.9 Hz, 2H), 2.45 (d, *J* = 1.5 Hz, 3H). ^13^C NMR (101 MHz, CDCl_3_) *δ* 156.38, 142.79, 136.45, 135.84, 134.23, 132.52, 129.27, 128.45, 127.71, 127.65, 122.88, 122.50, 119.77, 117.47, 110.28, 47.37, 18.35. HR-MS (EI) *m/z* calcd 274.3670; found, 275.3678 [M + H]^+^.

#### 1-Allyl-2-(3-phenoxyphenyl)-1H-benzo[d]imidazole (8d)

In a similar procedure as described for compound **8a**, yield of 76%. ^1^H NMR (400 MHz, CDCl_3_) *δ* 8.84 (s, 2H), 8.12–8.00 (m, 2H), 7.97–7.86 (m, 1H), 7.59–7.42 (m, 4H), 7.42–7.22 (m, 4H), 7.21–7.02 (m, 3H), 5.97 (ddt, *J* = 17.2, 10.5, 4.4 Hz, 1H), 5.23 (ddt, *J* = 10.5, 1.9, 1.0 Hz, 1H), 5.01 (dtd, *J* = 17.2, 2.0, 0.8 Hz, 1H), 4.80 (dt, *J* = 4.1, 1.9 Hz, 1H). ^13^C NMR (101 MHz, CDCl_3_) *δ* 171.55, 158.02, 157.20, 152.95, 142.07, 135.62, 132.01, 130.13, 127.31, 125.48, 124.08, 124.01, 123.45, 123.15, 120.36, 119.00, 117.73, 110.51, 47.26. HR-MS (EI) *m/z* calcd 326.3990; found, 327.3997 [M + H]^+^.

#### 1-Allyl-2-(naphthalen-2-yl)-1H-benzo[d]imidazole (8e)

In a similar procedure as described for compound **8a**, yield of 81%. ^1^H NMR (400 MHz, CDCl_3_) *δ* 8.25 (d, *J* = 1.7 Hz, 1H), 8.01–7.87 (m, 2H), 7.91–7.81 (m, 3H), 7.58–7.46 (m, 2H), 7.46–7.21 (m, 3H), 6.15–6.01 (m, 1H), 5.38–5.29 (m, 1H), 5.16–5.06 (m, 1H), 4.83 (dq, *J* = 3.6, 1.7 Hz, 2H). ^13^C NMR (101 MHz, CDCl_3_) *δ* 153.88, 143.17, 136.11, 133.78, 133.00, 132.47, 129.15, 128.64, 128.57, 127.88, 127.30, 126.80, 123.06, 122.73, 119.97, 117.64, 110.45, 47.36. HR-MS (EI) *m/z* calcd 284.3620; found, 385.3630 [M + H]^+^.

#### 1-Allyl-2-(4-phenoxyphenyl)-5-(trifluoromethyl)-1H-benzo[d]imidazole (12a)

The synthesis was performed according to the following steps. The *o*-phenylenediamine and derivatives **9** (108 mg, 1 mmol) were reacted with 4-phenoxybenzaldehyde or derivatives **10** (396 mg, 2 mmol) with sodium metabisulfite as catalyst to obtain compound **11**. Compound **11** (286 mg, 1 mmol) reacted with brominated alkyl group (240 mg, 2 mmol) to obtain the corresponding product compound **12a**, yield of 71%. ^1^H NMR (400 MHz, CDCl_3_) *δ* 8.12–8.06 (m, 1H), 7.78–7.70 (m, 2H), 7.52 (dd, *J* = 8.5, 1.7 Hz, 1H), 7.39 (ddd, *J* = 8.5, 7.5, 2.2 Hz, 3H), 7.22–7.15 (m, 1H), 7.15–7.05 (m, 4H), 6.07 (ddt, *J* = 17.2, 10.5, 4.3 Hz, 1H), 5.34 (dd, *J* = 10.5, 2.0 Hz, 1H), 5.05 (dt, *J* = 17.2, 2.0 Hz, 1H), 4.85 (dt, *J* = 4.1, 2.0 Hz, 2H). ^13^C NMR (101 MHz, CDCl_3_) *δ* 159.72, 156.04, 155.42, 142.51, 137.97, 131.93, 130.91, 130.08, 124.36, 123.79, 123.58, 119.89, 118.38, 117.83, 110.79, 47.38. HR-MS (EI) *m/z* calcd 394.3972; found, 395.3981 [M + H]^+^.

#### 1-Allyl-5-methyl-2-(4-phenoxyphenyl)-1H-benzo[d]imidazole (12b)

In a similar procedure as described for compound **12a**, yield of 83%. ^1^H NMR (400 MHz, CDCl_3_) *δ* 7.71 (dd, *J* = 12.3, 8.7 Hz, 2H), 7.61 (s, 0H), 7.44–7.31 (m, 2H), 7.31–7.04 (m, 5H), 7.07 (s, 0H), 6.07 (ddt, *J* = 17.2, 9.4, 4.7 Hz, 1H), 5.38–5.26 (m, 1H), 5.08 (dq, *J* = 15.2, 2.3 Hz, 1H), 4.79 (dq, *J* = 6.5, 2.1 Hz, 2H), 2.50 (d, *J* = 2.1 Hz, 3H). ^13^C NMR (101 MHz, CDCl_3_) *δ* 159.06, 159.02, 156.31, 153.35, 153.00, 143.31, 141.11, 134.08, 132.87, 132.28, 129.98, 124.31, 124.18, 124.07, 119.66, 118.38, 117.38, 110.18, 47.08, 21.64. HR-MS (EI) *m/z* calcd 338.4540; found, 339.4547 [M + H]^+^.

#### (1-Allyl-2-(4-phenoxyphenyl)-1H-benzo[d]imidazol-5-yl)(phenyl)methanone (12c)

In a similar procedure as described for compound **12a**, yield of 79%. ^1^H NMR (400 MHz, CDCl_3_) *δ* 8.19–8.14 (m, 1H), 7.80 (dd, *J* = 8.5, 1.6 Hz, 1H), 7.77–7.69 (m, 2H), 7.69–7.62 (m, 2H), 7.52–7.43 (m, 1H), 7.42–7.24 (m, 5H), 7.12–7.04 (m, 1H), 7.04–6.95 (m, 4H), 6.00 (ddt, *J* = 17.2, 10.5, 4.3 Hz, 1H), 5.30–5.21 (m, 1H), 5.04–4.94 (m, 1H), 4.78 (dt, *J* = 4.2, 2.0 Hz, 2H). ^13^C NMR (101 MHz, CDCl_3_) *δ* 195.58, 158.50, 154.95, 154.23, 141.25, 137.99, 137.26, 131.24, 130.95, 130.92, 129.74, 128.97, 128.96, 127.12, 123.94, 123.21, 122.86, 122.30, 118.75, 117.25, 116.71, 109.28, 46.29. HR-MS (EI) *m/z* calcd 430.5070; found, 431.5076 [M + H]^+^.

#### 1-Allyl-5,6-dimethyl-2-(4-phenoxyphenyl)-1H-benzo[d]imidazole (12d)

In a similar procedure as described for compound **12a**, yield of 83%. ^1^H NMR (400 MHz, DMSO) *δ* 7.81–7.71 (m, 2H), 7.51–7.40 (m, 3H), 7.29–7.17 (m, 2H), 7.17–7.08 (m, 4H), 6.08 (ddt, *J* = 16.7, 10.3, 4.5 Hz, 1H), 5.17 (ddd, *J* = 23.5, 10.4, 1.6 Hz, 1H), 4.90–4.79 (m, 3H), 4.43 (dt, *J* = 5.1, 1.7 Hz, 1H), 2.33 (d, *J* = 6.6 Hz, 5H), 2.22 (s, 1H). ^13^C NMR (101 MHz, DMSO) *δ* 158.55, 156.25, 153.56, 141.68, 134.94, 133.24, 131.58, 130.71, 127.43, 118.53, 116.99, 111.28, 42.99, 20.63. HR-MS (EI) *m/z* calcd 354.4530; found, 355.4531 [M + H]^+^.

#### 2-(4-Phenoxyphenyl)-1-(prop-2-yn-1-yl)-5-(trifluoromethyl)-1H-benzo[d]imidazole (12e)

In a similar procedure as described for compound **12a**, yield of 78%. ^1^H NMR (400 MHz, CDCl_3_) *δ* 8.12–8.06 (m, 1H), 7.78–7.70 (m, 2H), 7.52 (dd, *J* = 8.5, 1.7 Hz, 1H), 7.39 (ddd, *J* = 8.5, 7.5, 2.2 Hz, 3H), 7.22–7.15 (m, 1H), 7.15–7.05 (m, 4H), 6.07 (ddt, *J* = 17.2, 10.5, 4.3 Hz, 1H), 5.34 (dd, *J* = 10.5, 2.0 Hz, 1H), 5.05 (dt, *J* = 17.2, 2.0 Hz, 1H), 4.85 (dt, *J* = 4.1, 2.0 Hz, 2H). ^13^C NMR (101 MHz, CDCl_3_) *δ* 160.04, 156.02, 155.35, 145.26, 135.04, 131.32, 130.20, 126.23, 125.54, 125.22, 124.54, 124.54, 123.31, 120.42, 120.07, 118.58, 107.86, 76.90, 74.86, 35.18. HR-MS (EI) *m/z* calcd 392.3812; found, 393.3816 [M + H]^+^.

#### 1-(Cyclopropylmethyl)-2-(4-phenoxyphenyl)-5-(trifluoromethyl)-1H-benzo[d]imidazole (12f)

In a similar procedure as described for compound **12a**, yield of 84%. ^1^H NMR (400 MHz, CDCl_3_) *δ* 8.08 (dt, *J* = 1.6, 0.8 Hz, 1H), 7.74–7.65 (m, 2H), 7.55 (t, *J* = 1.3 Hz, 2H), 7.46–7.33 (m, 2H), 7.24–7.06 (m, 5H), 4.18 (d, *J* = 6.6 Hz, 2H), 1.27–1.00 (m, 1H), 0.62–0.50 (m, 2H), 0.27–0.19 (m, 2H). ^13^C NMR (101 MHz, CDCl_3_) *δ* 159.46, 156.21, 155.43, 142.69, 137.95, 130.69 (d, *J* = 106.8 Hz), 128.14–121.86 (m), 119.21 (d, *J* = 138.3 Hz), 110.83, 49.38, 11.43, 4.56. HR-MS (EI) *m/z* calcd 408.1410; found, 408.1416 [M + H]^+^.

#### 1-Allyl-2-(4-(pyridin-3-yloxy)phenyl)-5-(trifluoromethyl)-1H-benzo[d]imidazole (12g)

In a similar procedure as described for compound **12a**, yield of 83%. ^1^H NMR (400 MHz, CDCl_3_) *δ* 8.52–8.42 (m, 2H), 8.10 (d, *J* = 1.7 Hz, 1H), 7.84–7.74 (m, 2H), 7.56 (dd, *J* = 8.5, 1.7 Hz, 1H), 7.48–7.36 (m, 2H), 7.35 (ddd, *J* = 8.4, 4.7, 0.8 Hz, 1H), 7.21–7.09 (m, 2H), 6.10 (ddt, *J* = 17.1, 10.5, 4.3 Hz, 1H), 5.37 (dt, *J* = 10.4, 1.9 Hz, 1H), 5.07 (dt, *J* = 17.2, 2.1 Hz, 1H), 4.92–4.81 (m, 2H). ^13^C NMR (101 MHz, CDCl_3_) *δ* 158.64, 155.01, 152.87, 145.35, 142.29 (d, *J* = 35.8 Hz), 137.89, 131.81, 131.13, 125.47 (d, *J* = 222.6 Hz), 124.86, 118.65, 110.74, 47.35. HR-MS (EI) *m/z* calcd 408.1410; found, 408.1418 [M + H]^+^.

#### 1-Allyl-2-(6-phenoxypyridin-3-yl)-5-(trifluoromethyl)-1H-benzo[d]imidazole (12h)

In a similar procedure as described for compound **12a**, yield of 77%. ^1^H NMR (400 MHz, CDCl_3_) *δ* 8.55 (d, *J* = 2.4 Hz, 1H), 8.17 (dd, *J* = 8.6, 2.5 Hz, 1H), 8.14–8.08 (m, 1H), 7.56 (dd, *J* = 8.6, 1.7 Hz, 1H), 7.51–7.39 (m, 3H), 7.32–7.15 (m, 3H), 7.09 (d, *J* = 8.6 Hz, 1H), 6.06 (ddt, *J* = 17.2, 10.5, 4.2 Hz, 1H), 5.34 (dt, *J* = 10.4, 1.9 Hz, 1H), 5.02 (dt, *J* = 17.1, 2.0 Hz, 1H), 4.87 (dt, *J* = 4.1, 2.0 Hz, 2H). ^13^C NMR (101 MHz, CDCl_3_) *δ* 164.93, 153.45, 152.73, 147.78, 142.54, 140.59, 137.89, 130.73 (d, *J* = 173.7 Hz), 125.40, 119.73 (d, *J* = 352.9 Hz), 111.75, 110.79, 47.29. HR-MS (EI) *m/z* calcd 395.3852; found, 396.3852 [M + H]^+^.

#### 1-Allyl-6-fluoro-5-methyl-2-(6-phenoxypyridin-3-yl)-1H-benzo[d]imidazole (12i)

In a similar procedure as described for compound **12a**, yield of 75%. ^1^H NMR (400 MHz, CDCl_3_) *δ* 7.75–7.50 (m, 3H), 7.42–7.29 (m, 2H), 7.21–7.01 (m, 5H), 6.97 (d, *J* = 9.4 Hz, 1H), 6.03 (ddt, *J* = 17.2, 10.5, 4.4 Hz, 1H), 5.31 (dtd, *J* = 10.5, 1.9, 0.8 Hz, 1H), 5.07 (dtd, *J* = 17.2, 2.0, 0.8 Hz, 1H), 4.74 (dt, *J* = 4.2, 2.0 Hz, 2H), 2.43 (s, 3H). ^13^C NMR (101 MHz, CDCl_3_) *δ* 159.06, 159.02, 156.31, 153.35, 153.00, 143.31, 141.11, 134.08, 132.87, 132.28, 129.98, 124.31, 124.18, 124.07, 119.66, 118.38, 117.38, 110.18, 47.08, 21.64. HR-MS (EI) *m/z* calcd 358.4239; found, 359.4214 [M + H]^+^.

### NsdUTPase activity assay

The *in vitro* catalytic activity of *Ns*dUTPase was detected by a PPi Light inorganic pyrophosphate assay kit following manufactory’s manual. All the reaction components were added to a 96-well white plate, including 2 µL 40 ng/mL *Ns*dUTPase, 36 µL reaction buffer (25 mM Tris–HCl, pH 8.0, 10 mM KCl, 1.25 mM MgCl_2_, 0.1 mg/mL BSA, 0.005% Triton X, and 20% glycerol), and 2 µL 10 µM dUTP. After 120 min of incubation at room temperature, the luminescence intensity of the reaction was measured by a microplate luminometer at 560 nm.

### *In vitro* anti-*Nocardia* activity

The MABA method was used to determine the anti-*Nocardia* activity of compounds according to reported method^34^ with modification. *N. seriolae* strain JCM3360, and *N. salmonicida* strain JCM4826 were gifted from Prof. Nien, Chung-Yi (National Central University, Taoyuan City, Taiwan), and cultured in M-196 medium (0.4% yeast extract; 1% malt extract; 0.4% dextrose) at 25 °C. Two strains were inoculated into 96-well plates at a concentration of ×10^6^ CFU/mL and cultured in the medium, then different concentrations of compounds were added. After two days of incubation at 25 °C, the mixture of 20 μL 10× alamar blue and 50 μL 5% Tween-80 were added to observe the colour change. Minimum inhibitory concentration (MIC) is defined as the minimum compound concentration of the well varying from blue to pink.

### Cytotoxicity of compound candidates

The cytotoxicity of compounds **4b** and **12b** was determined by CCK-8 assay. A549 and Marc cells were cultured for the test. Concentrations of compounds varied from 0.03 to 30 µM. Meanwhile, 1% DMSO was used as a negative control. After incubation for 24 h, WST-8 was added. The experimental method referred to the kit instructions. All the experiments were repeated three times.

### Statistical analysis

IC_50_ was determined using GraphPad Prism software 9 (GraphPad Software, San Diego, CA). All experiments were performed in triplicate, and values (±standard deviations [SD]) are shown. Significant differences were determined using Student’s *t*-test. *p < 0.05 (considered significant compared with control group); **p < 0.01 (considered highly significant); ***p < 0.001 (considered extremely significant).

### Molecular dynamics simulations

The MD simulations were performed using Amber 22^35^. The proteins and ligands were treated with ff14SB force field and *gaff* force field, respectively. The protein–ligand systems were energy minimised using 3000 steps steepest descent algorithm and 3000 steps conjugate gradient algorithm. After minimisation, the four systems were heated to 300 K during 500 ps in NVT ensemble. The pressure was then equilibrated to 1 atm during a 500 ps NPT simulation. Production simulations were performed for 50 ns for each complex while the temperature and pressure were maintained at 300 K and 1 atm, respectively.

### Binding free energy calculation

The binding free energy was calculated using molecular mechanics (MM) Poisson–Boltzmann surface area (MM-PBSA) approach in Amber22 using MMPBSA.py module as following formula^36^:

$$\Delta G_{\text{binding}}=\Delta H-T\Delta S=\Delta E_{\text{MM}}+\Delta G_{\text{sol}}-T\Delta S$$

$$\Delta E_{\text{MM}}=\Delta E_{\text{ele}}+\Delta E_{\text{vdw}}$$

$$\Delta G_{\text{sol}}=\Delta G_{\text{PB}}+\Delta G_{\text{SA}}$$

In which $\Delta G_{\text{binding}}$ was the binding free energy of ligand, ΔH and −TΔS were the enthalpy and the entropy, respectively. The changes in the gas phase MM energy $\Delta E_{\text{MM}}$ could be calculated though electrostatic energies $\Delta E_{\text{ele}}$, and the van der Waals energies $\Delta E_{\text{vdw}}$. While solvation free energy $\Delta G_{\text{sol}}$ is the sum of the electrostatic solvation energy $\Delta G_{\text{PB}}$ (polar contribution) and the nonpolar contribution $\Delta G_{\text{SA}}$ between the solute and the continuum solvent. The polar contribution is calculated using the PB model, while the nonpolar energy is usually estimated using the solvent-accessible surface area (SASA).

## Supplementary Material

Supplemental Material

## Data Availability

The authors confirm that the data supporting the findings of this study are available within the article and its supplementary materials.
