# Supplementary material for: Structure-guided discovery of novel dUTPase inhibitors with anti-Nocardia activity by computational design
Source: J Enzyme Inhib Med Chem. 2024 Oct 10;39(1):2411573. doi: 10.1080/14756366.2024.2411573 (PMC11486130; doi:10.1080/14756366.2024.2411573)
Supplement: Supplemental Material [file IENZ_A_2411573_SM7817.pdf]

Supplementary Data

Structure-guided Discovery of novel dUTPase Inhibitors with Anti-Nocardia Activity by Computational Design

Zhi-Zheng Wang [a,†], Jun Weng [a,b,†], Jing Qi [a], Xin-Xin Fu [a], Ban-Bin Xing [a], Yang Hu [a], Chun-Hsiang Huang [c], Qingyu Chen [a], Zigong Wei [a,d,*]

[a.] State Key Laboratory of Biocatalysis and Enzyme Engineering, National & Local Joint Engineering Research Center of High-throughput Drug Screening Technology, School of life sciences, Hubei University, Wuhan, Hubei, *P. R.* China

[b.] Key Laboratory of Molecular Biophysics of Ministry of Education, National Engineering Research Center for Nanomedicine, College of Life Science and Technology, Huazhong University of Science and Technology, Wuhan, Hubei, *P. R.* China

[c.] Protein Diffraction Group, Experimental Facility Division, National Synchrotron Radiation Research Center, Hsinchu 30076, Taiwan

[d.] Hubei Jiangxia Laboratory, Wuhan, Hubei, *P. R.* China

[†] These authors contributed equally to this work.

***Corresponding author:**

Zigong Wei, E-mail address: weizigong@163.com

Table S1. The binding free energy of substituent optimization (kcal/mol) with *Ns*dUTPase.

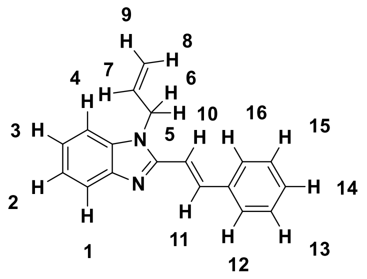

Δ*G*	Br	CF_3	CH_3	Cl	F	OCH_3
1	-0.34	0.37	0.56	0.29	0.85	1.93
2	-1.37	-3.86	-1.96	-1.42	-0.75	-2.61
3	-1.62	-0.16	-0.10	-0.77	0.49	0.48
4	-0.20	0.09	0.09	0.51	1.49	2.22
5	-1.55	2.27	0.49	-1.92	0.09	-1.26
6	-0.70	0.33	0.17	-0.27	0.44	-0.29
7	1	6.36	2.91	0.69	0.99	5.13
8	-1.34	-0.26	-2.11	-1.72	-0.56	0.98
9	-1.13	-0.16	0.01	-0.82	-0.28	-0.51
10	-1.34	-1.80	0.03	-0.93	0.35	-1.71
11	-0.41	0.63	-0.60	-0.81	1.42	1.33
12	-1.60	-0.57	-1.82	-1.52	0	-0.16
13	-1.21	-1.31	-0.82	-0.43	0.35	1.28
14	0.10	-1.96	0.61	0.11	-1.39	2.84
15	2.42	2.39	2.50	3.02	3.16	3.89
16	-1.10	-0.26	-0.22	0.03	0.92	0.70

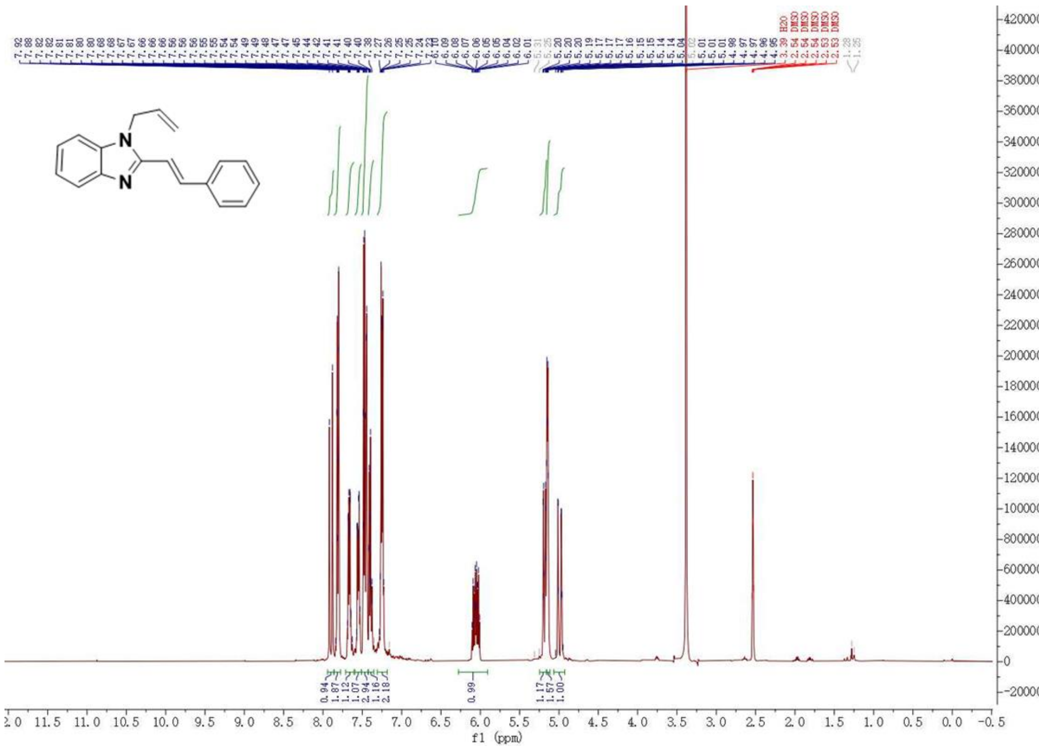

^{1}H NMR for compound F0414.

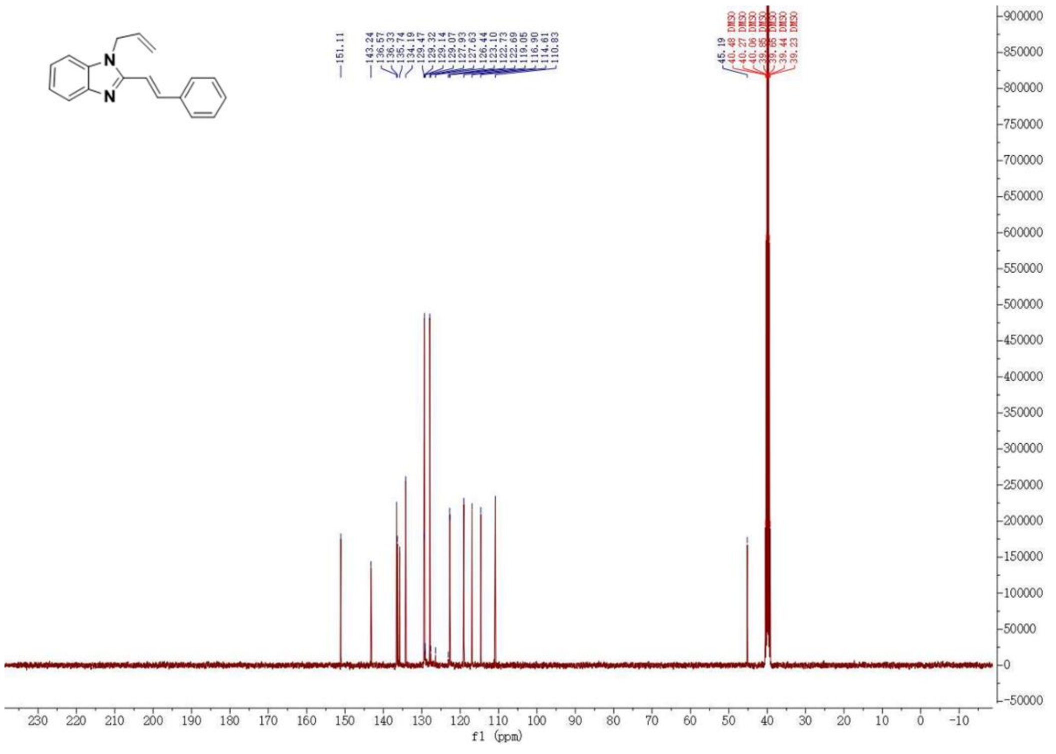

^{13}C NMR for compound F0414.

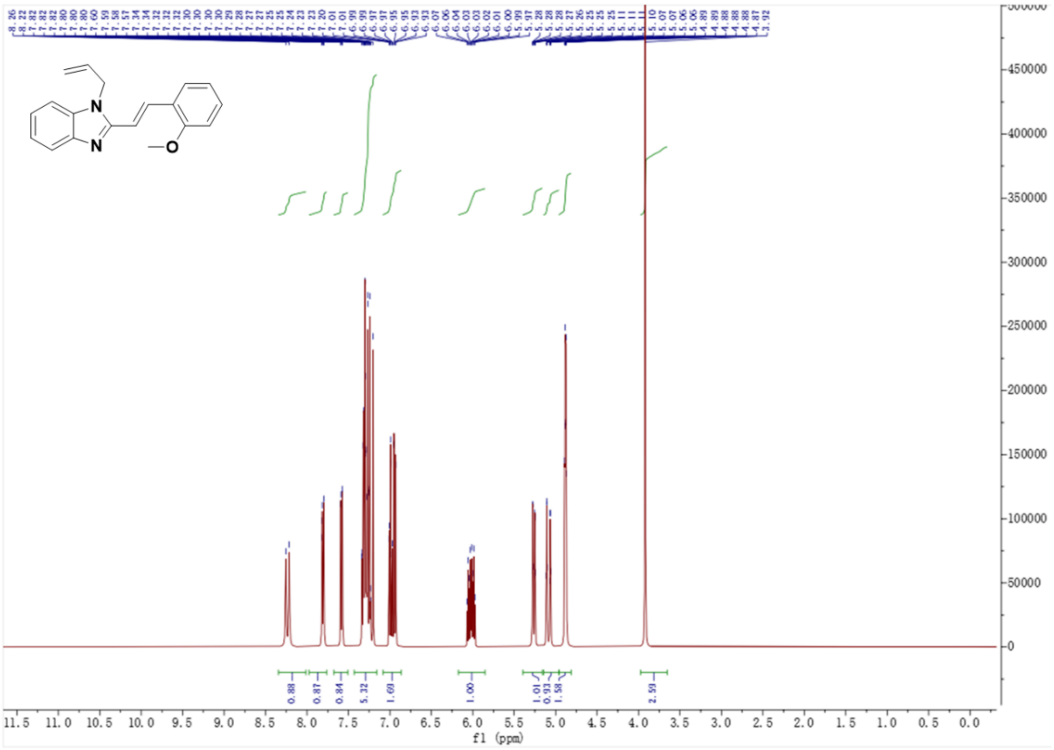

^{1}H NMR for compound **4a**.

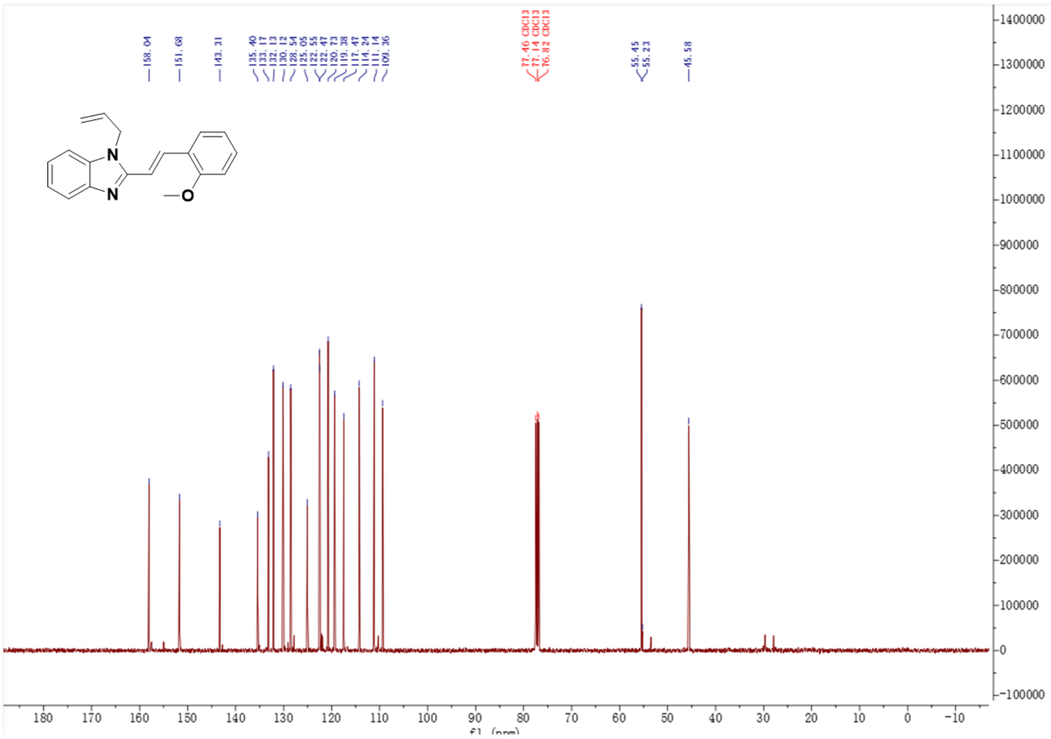

^{13}C NMR for compound **4a**.

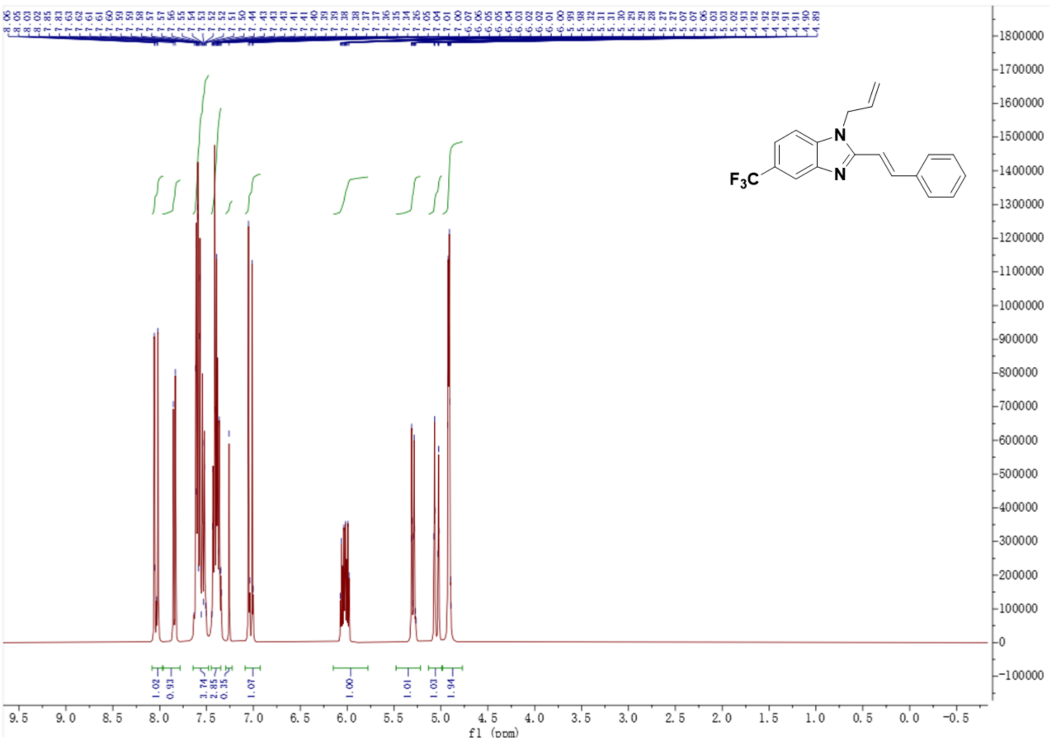

^{1}H NMR for compound **4b**.

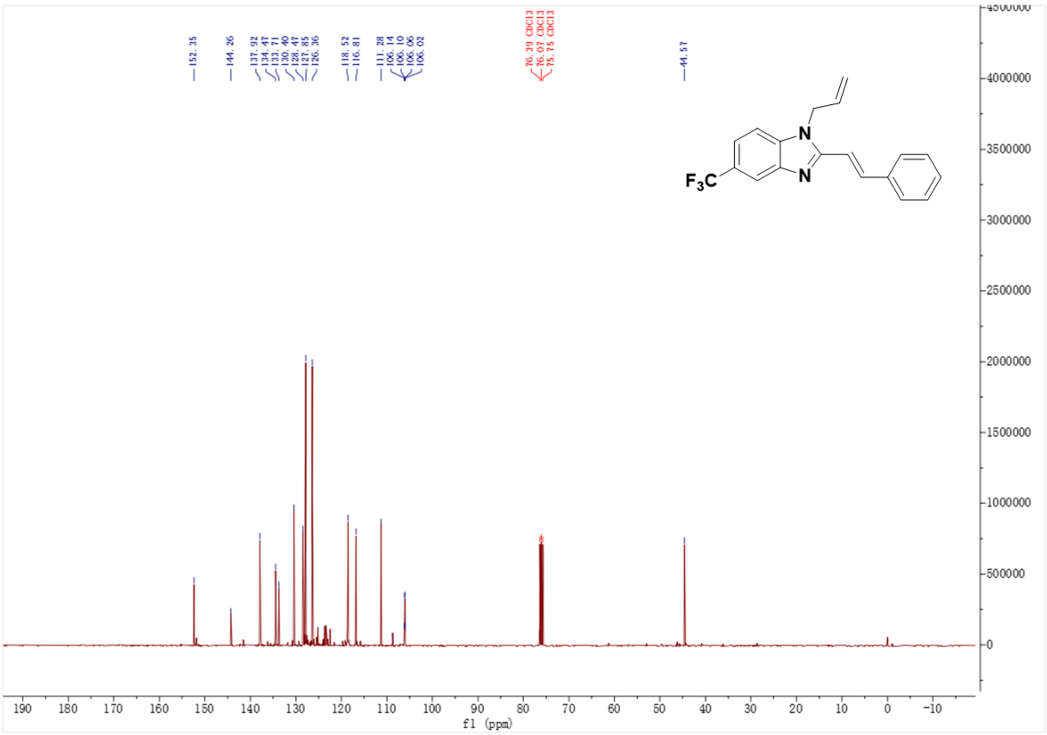

^{13}C NMR for compound **4b**.

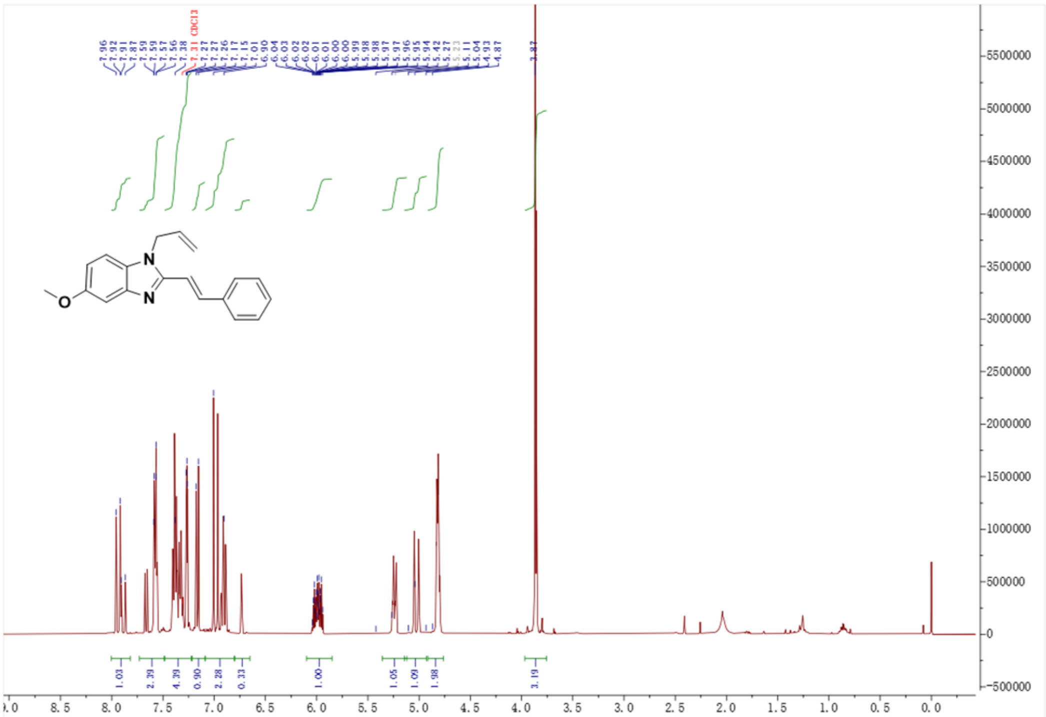

^{1}H NMR for compound **4c**.

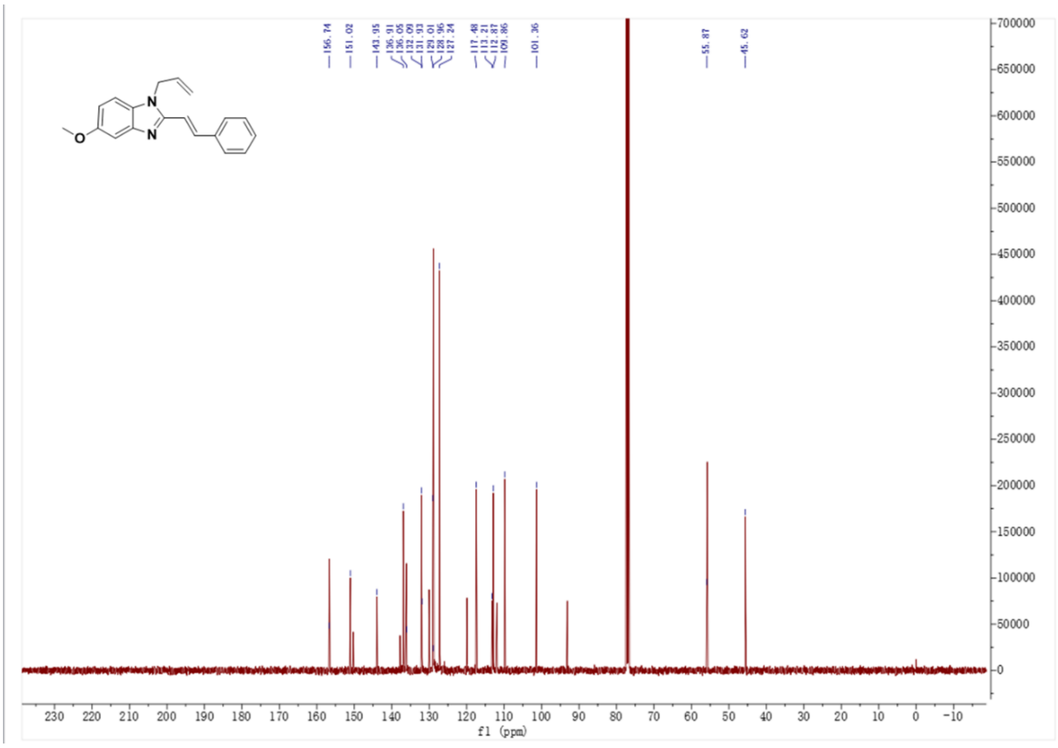

^{13}C NMR for compound **4c**.

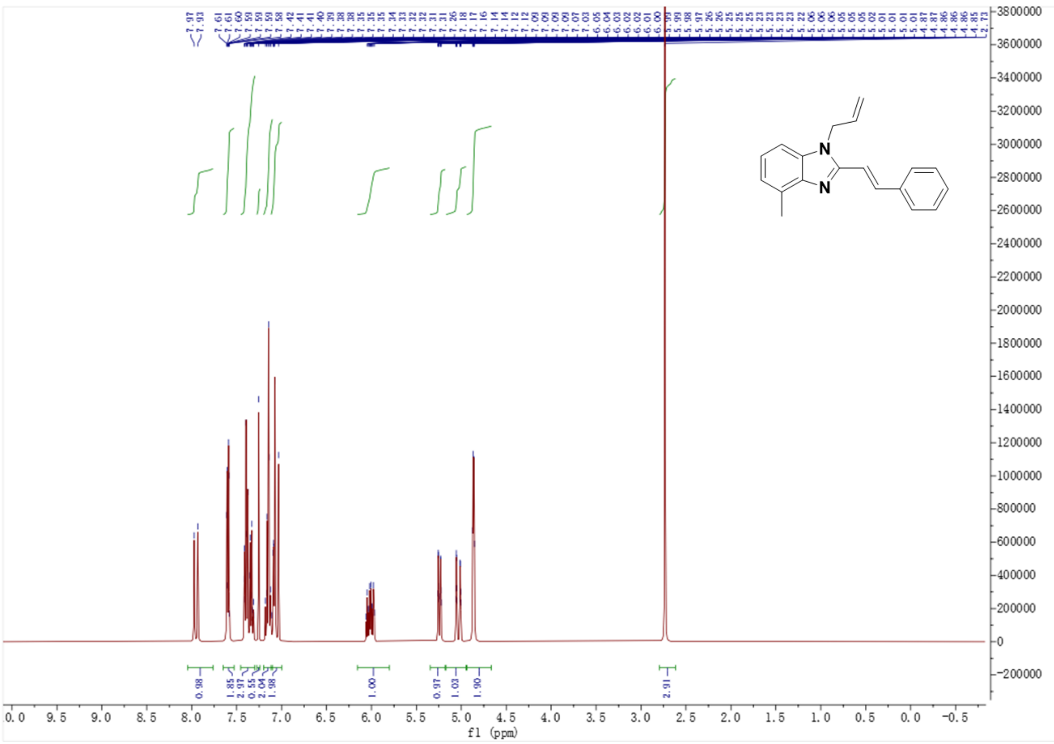

^{1}H NMR for compound **4d**.

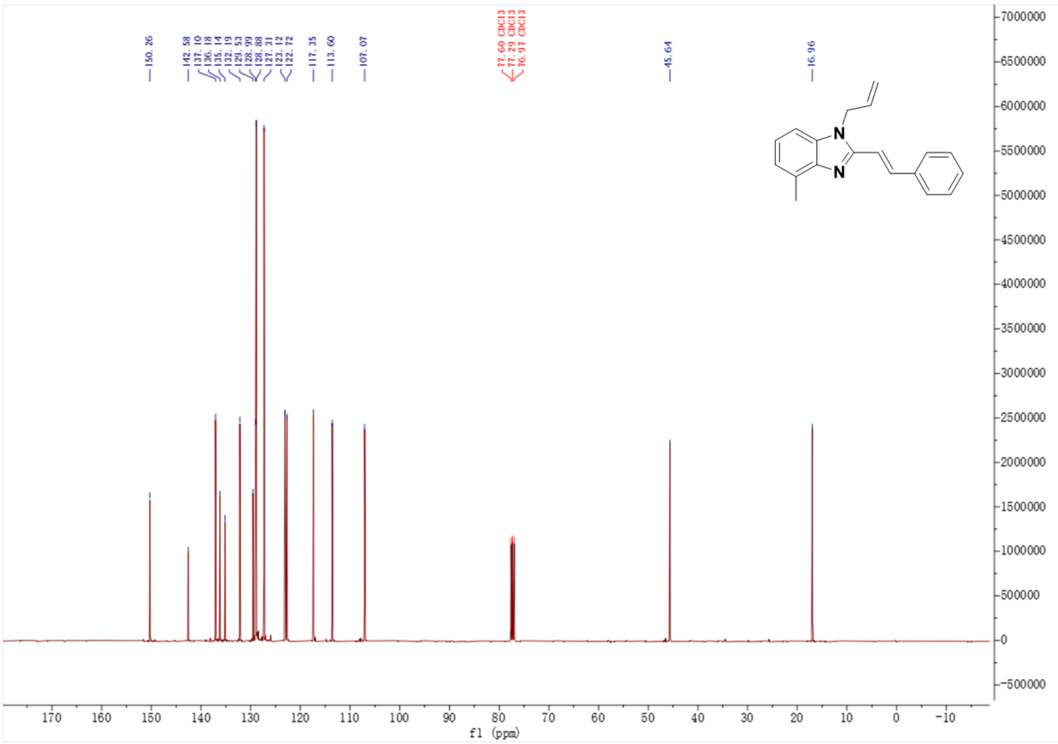

^{13}C NMR for compound **4d**.

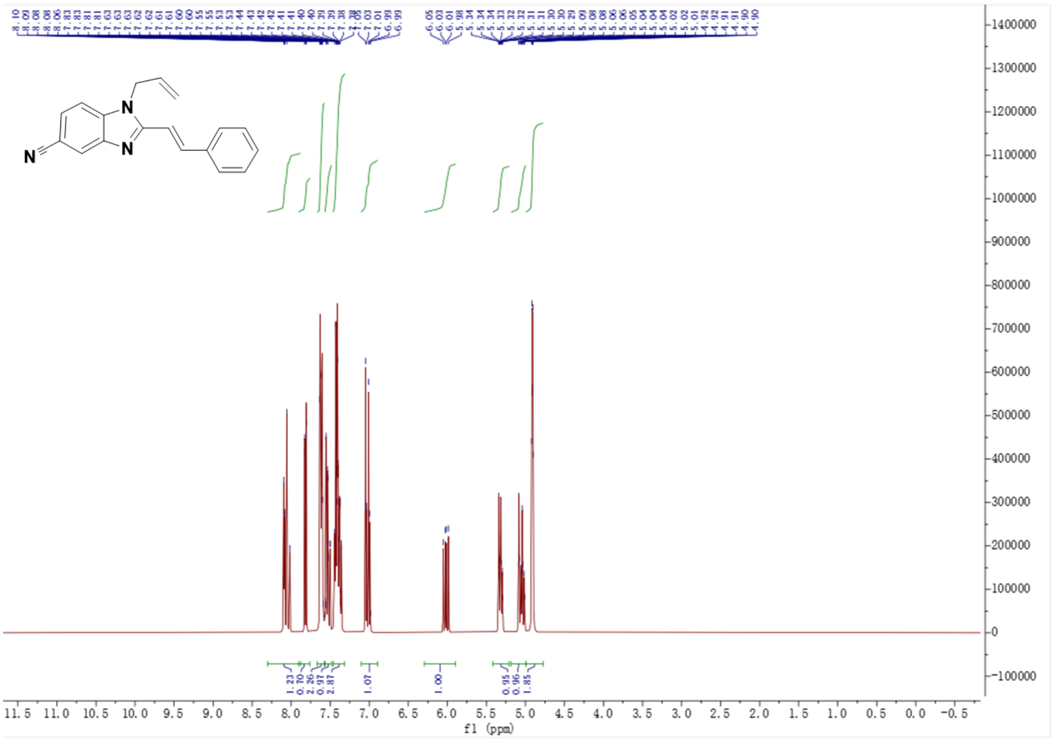

^{1}H NMR for compound **4e**.

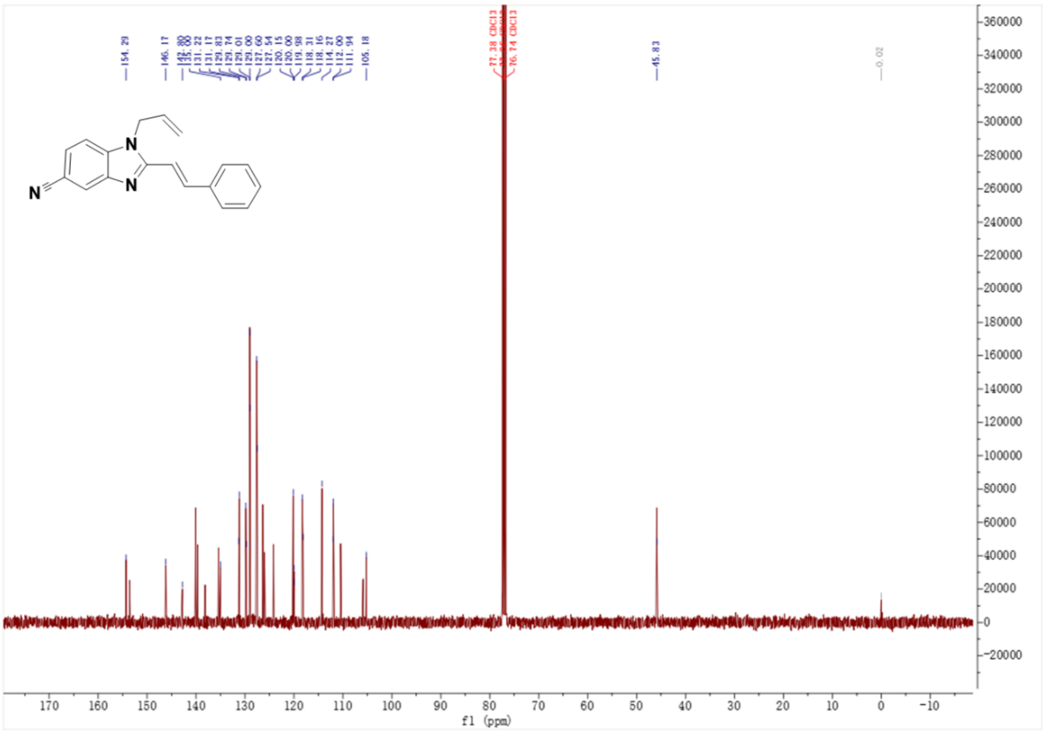

^{13}C NMR for compound **4e**.

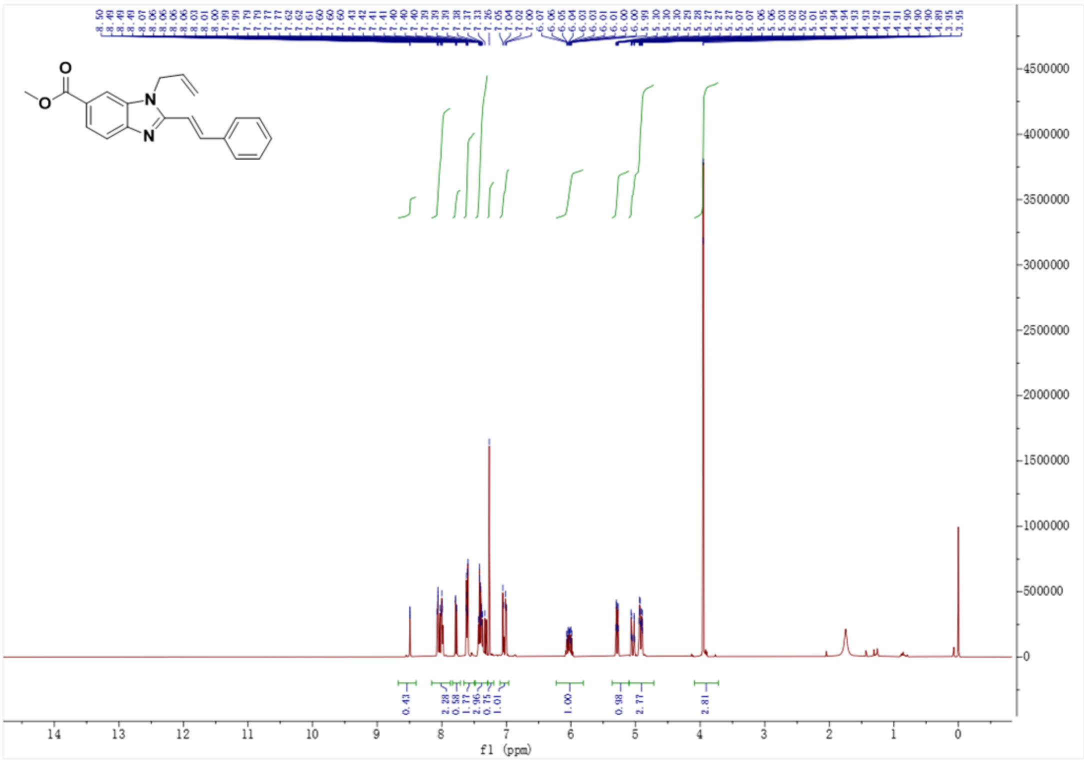

^{1}H NMR for compound **4f**.

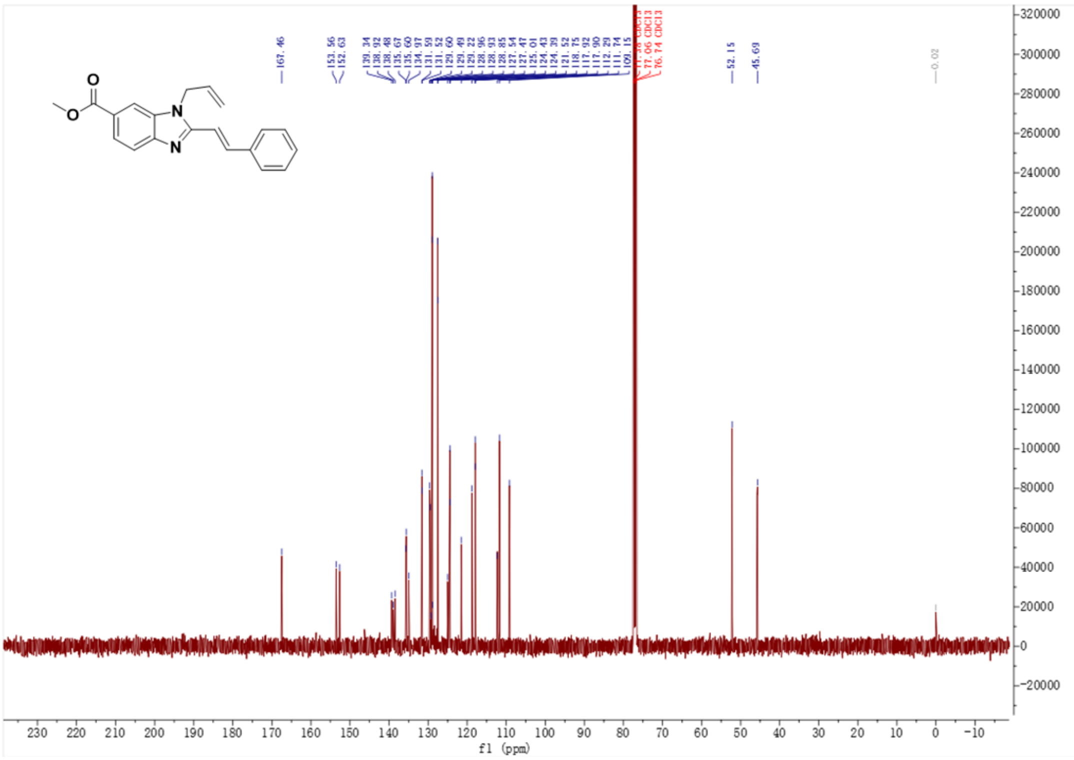

^{13}C NMR for compound **4f**.

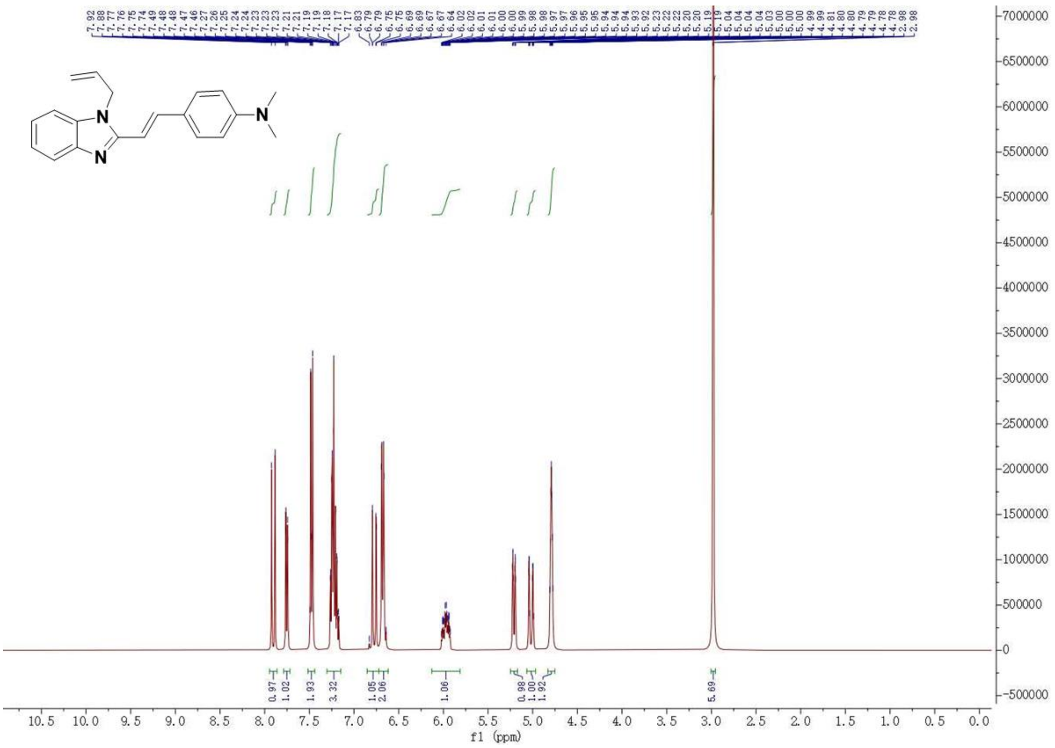

^{1}H NMR for compound **4g**.

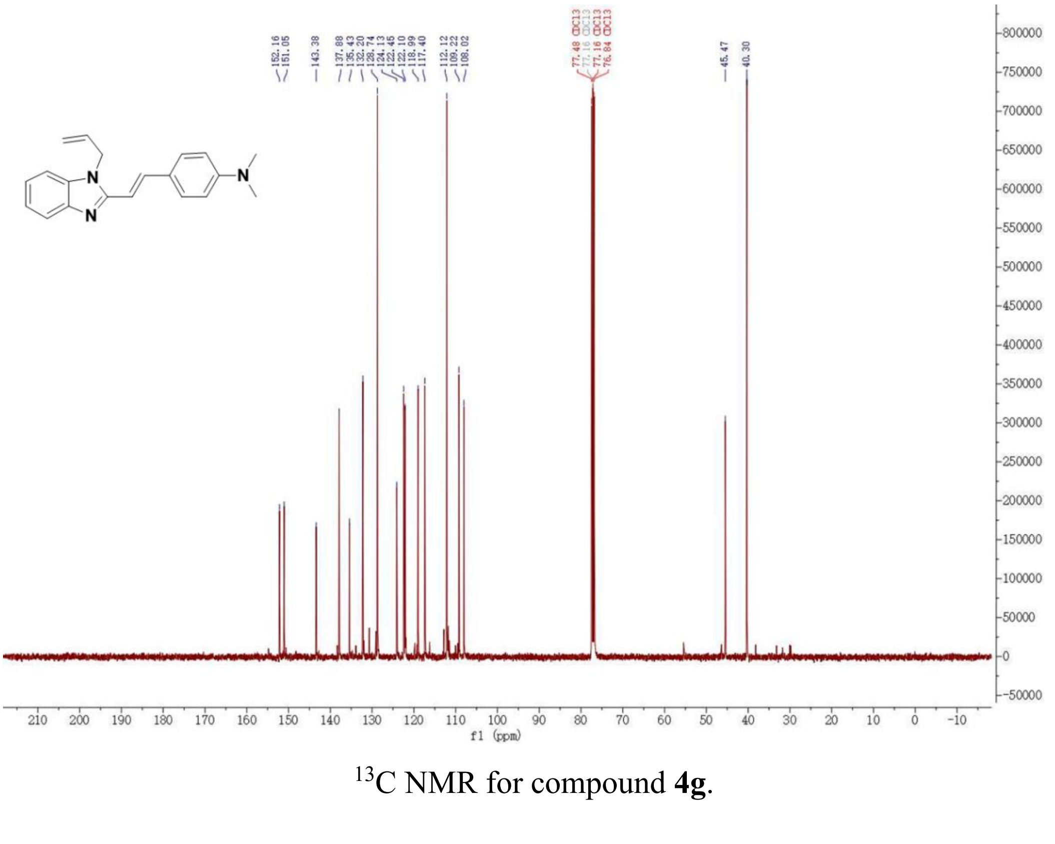

^{13}C NMR for compound **4g**.

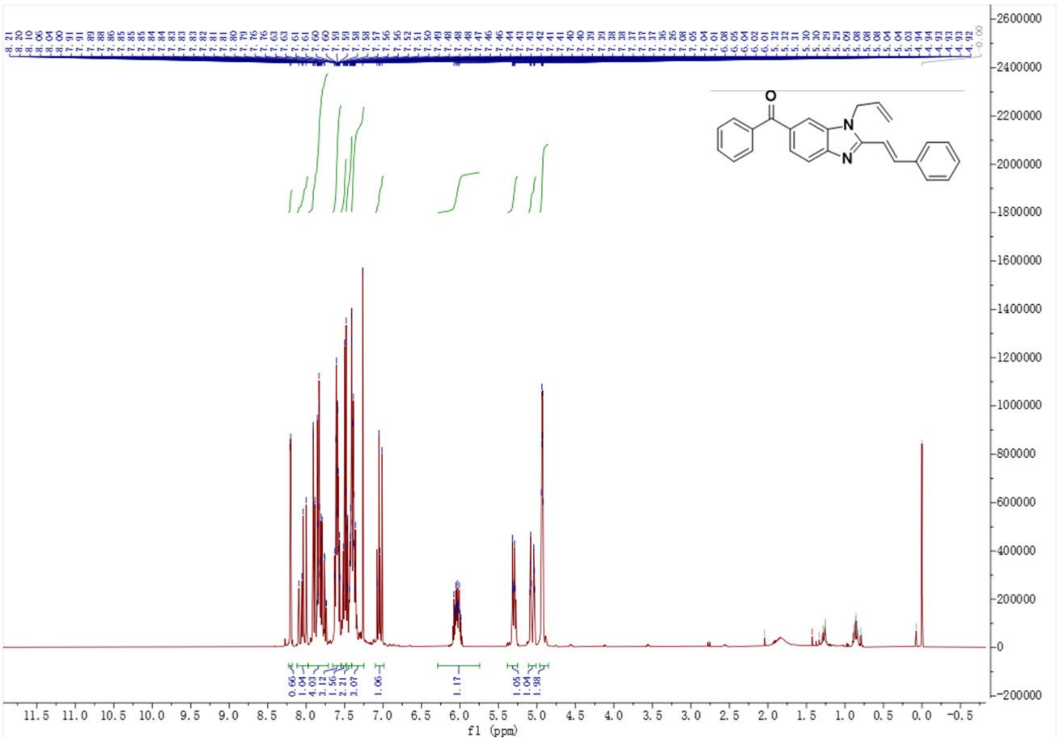

^{1}H NMR for compound **4h**.

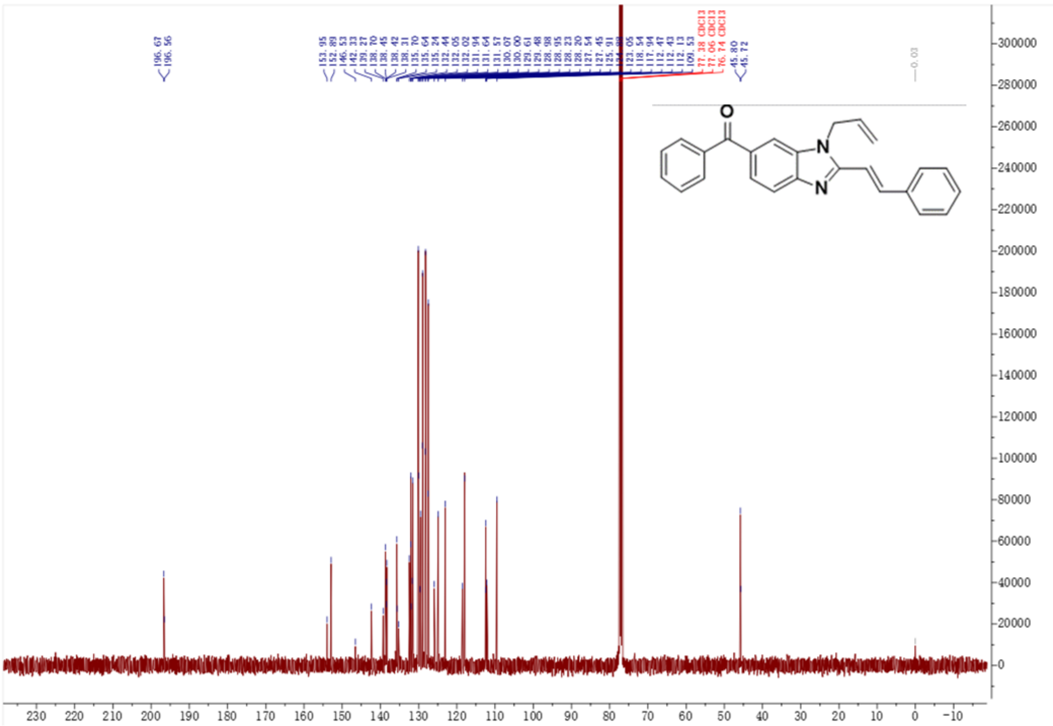

^{13}C NMR for compound **4h**.

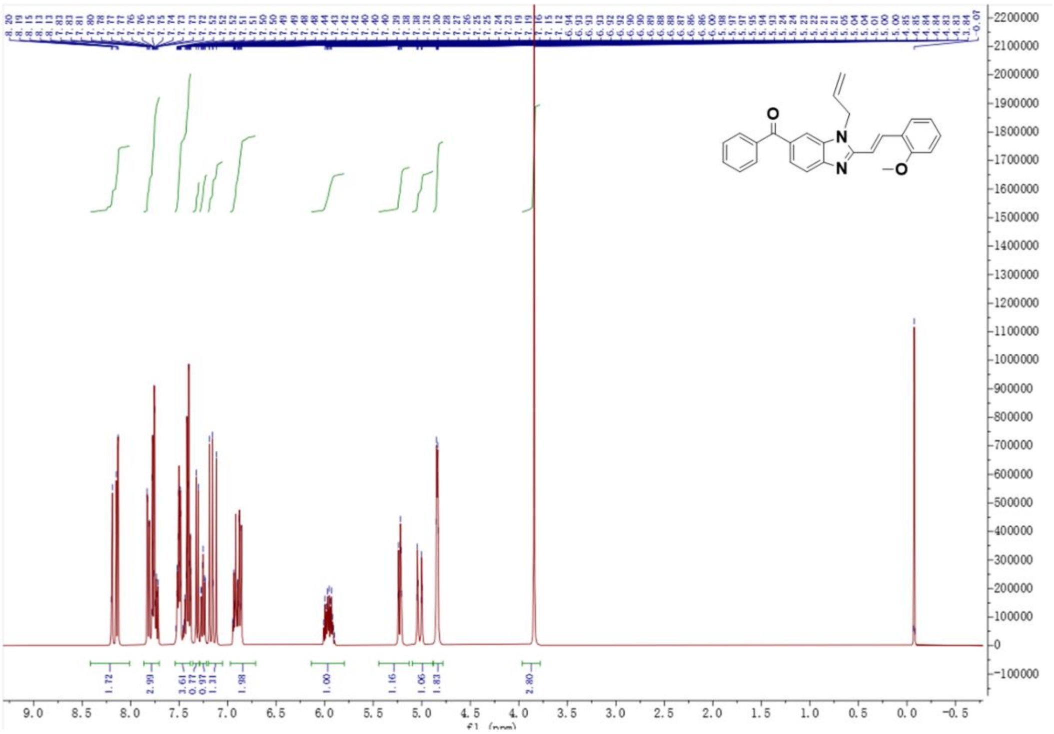

^{1}H NMR for compound **4i**.

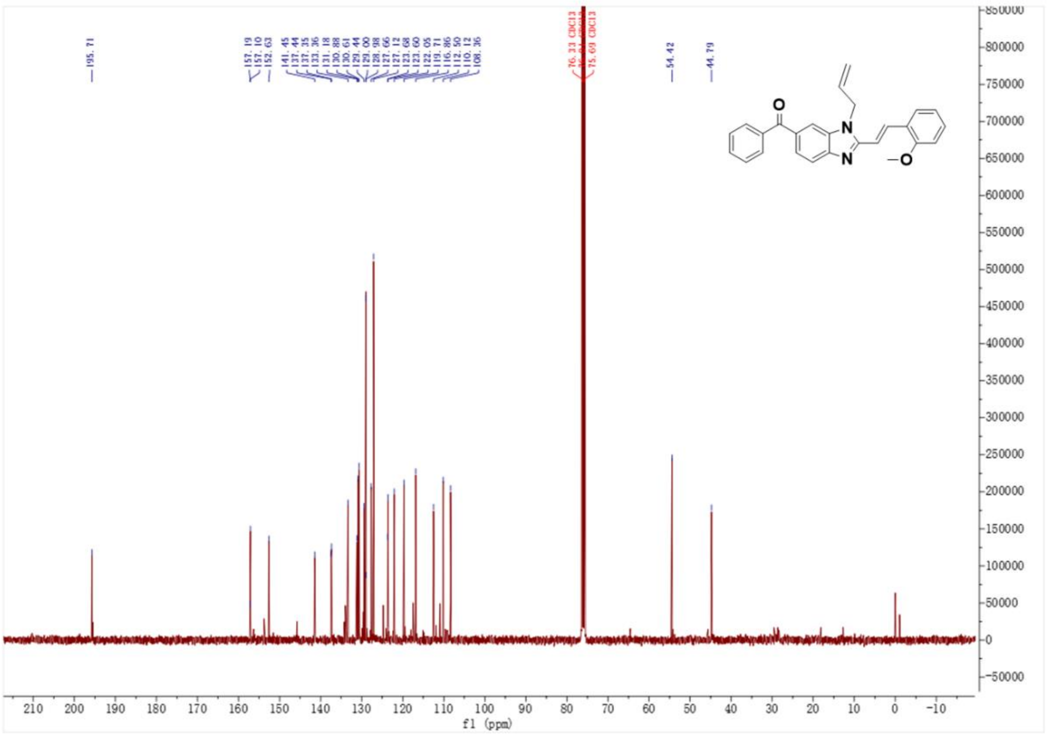

^{13}C NMR for compound **4i**.

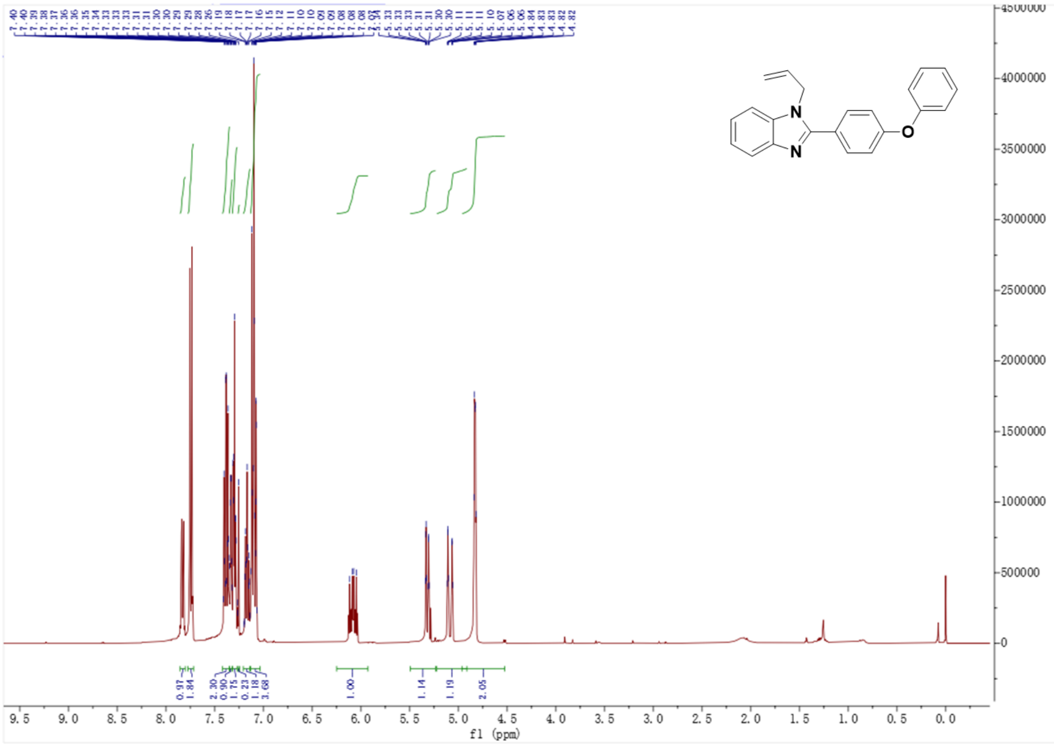

^{1}H NMR for compound **8a**.

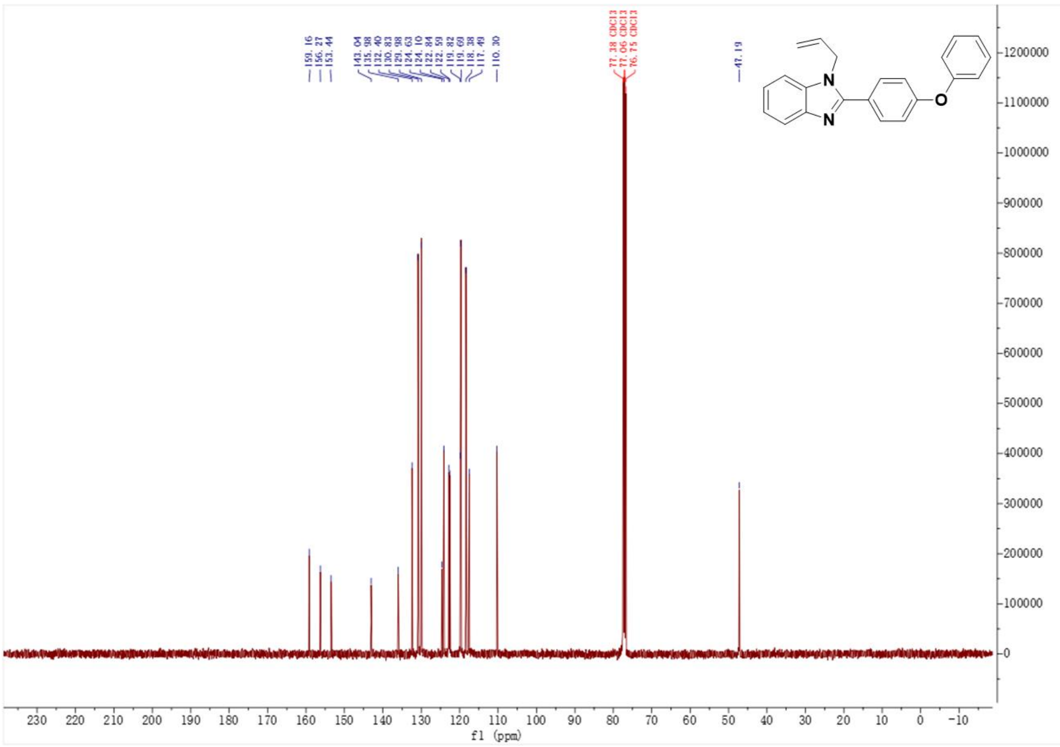

^{13}C NMR for compound **8a**.

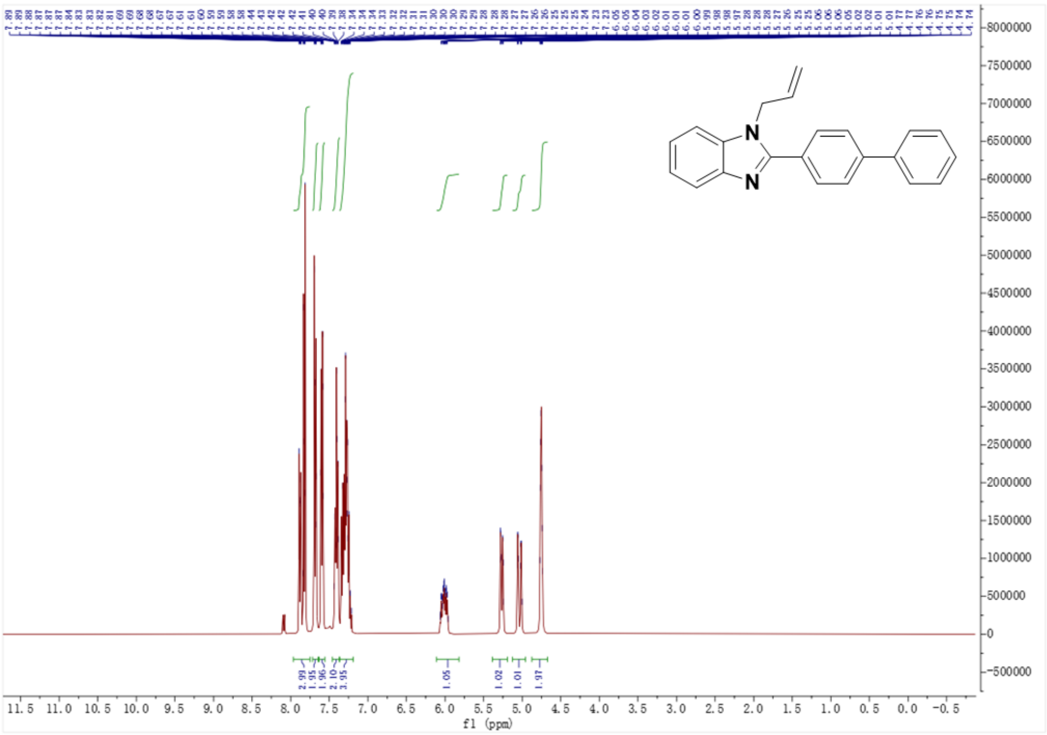

^{1}H NMR for compound **8b**.

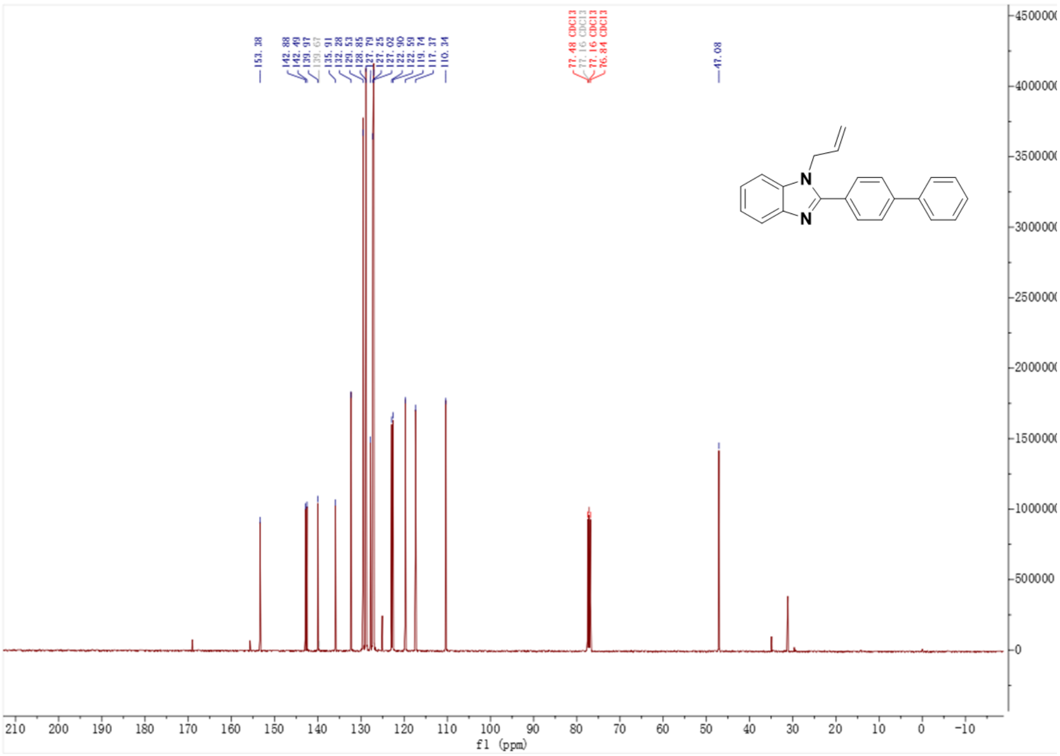

^{13}C NMR for compound **8b**.

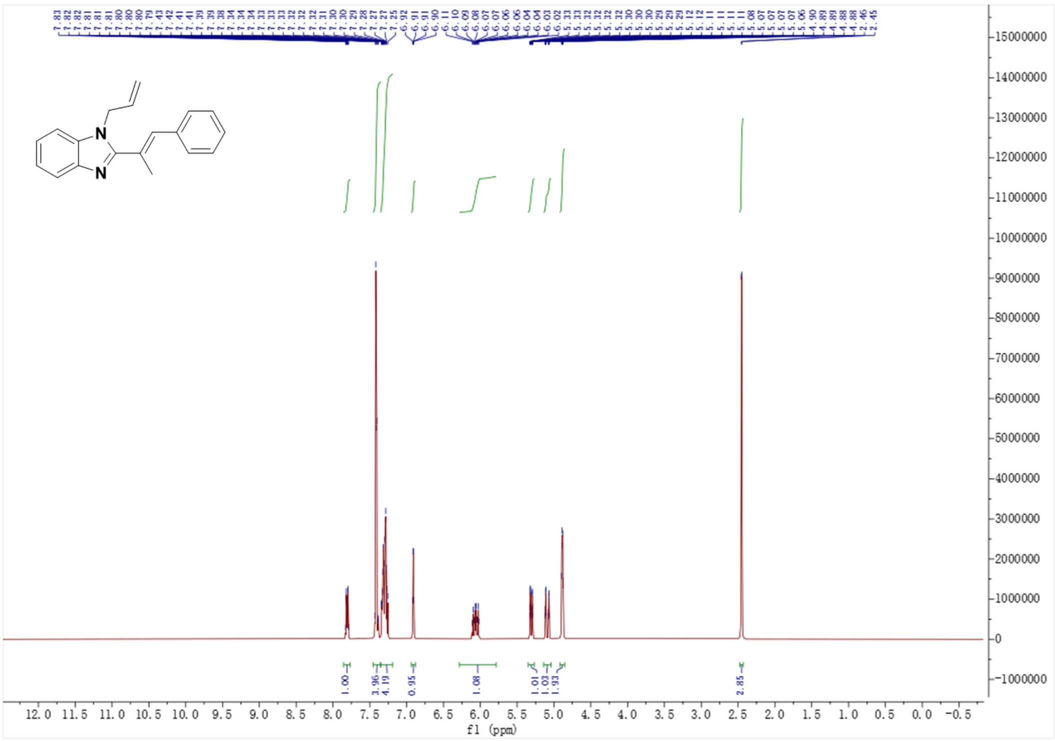

^{1}H NMR for compound **8c**.

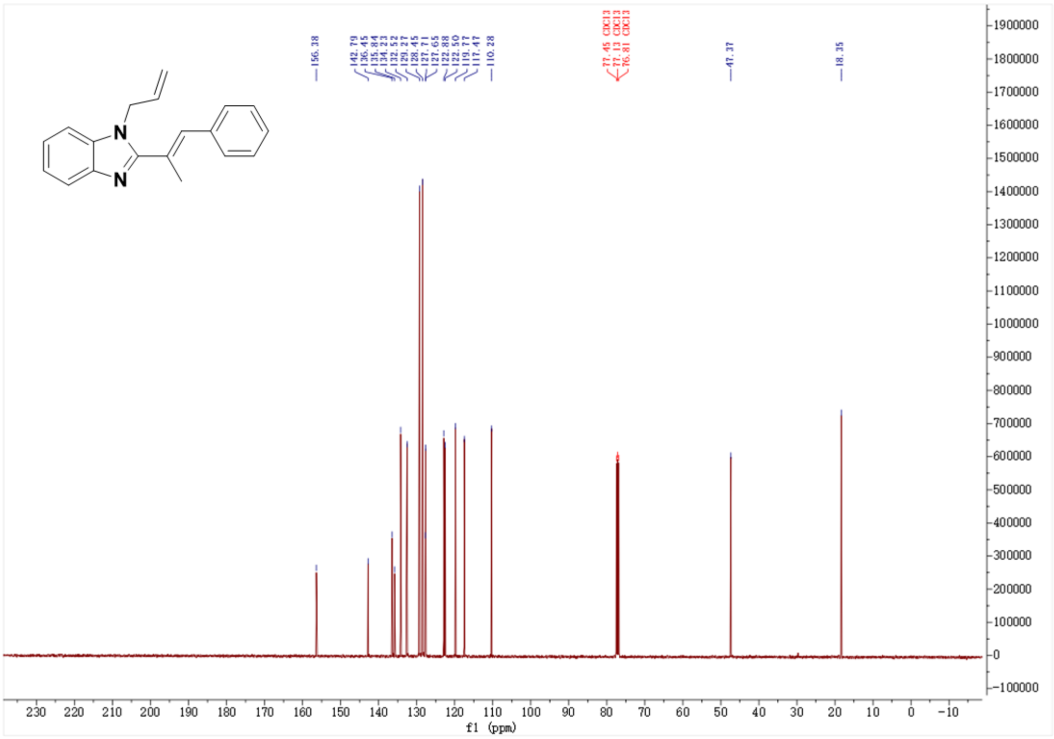

^{13}C NMR for compound **8c**.

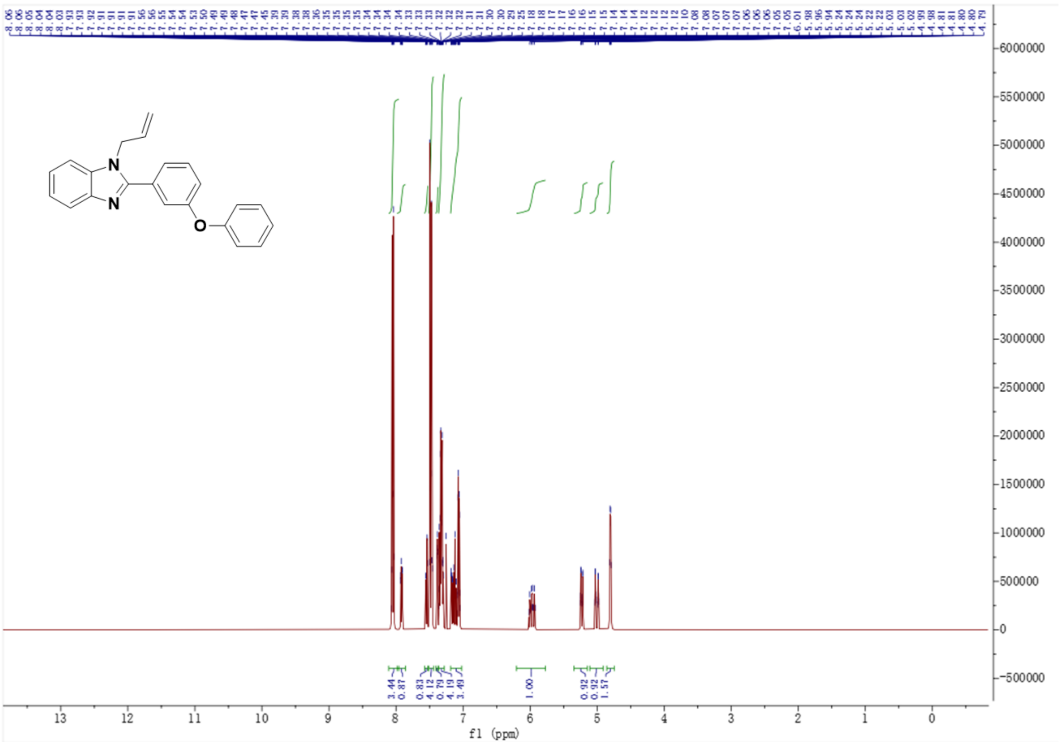

^{1}H NMR for compound **8d**.

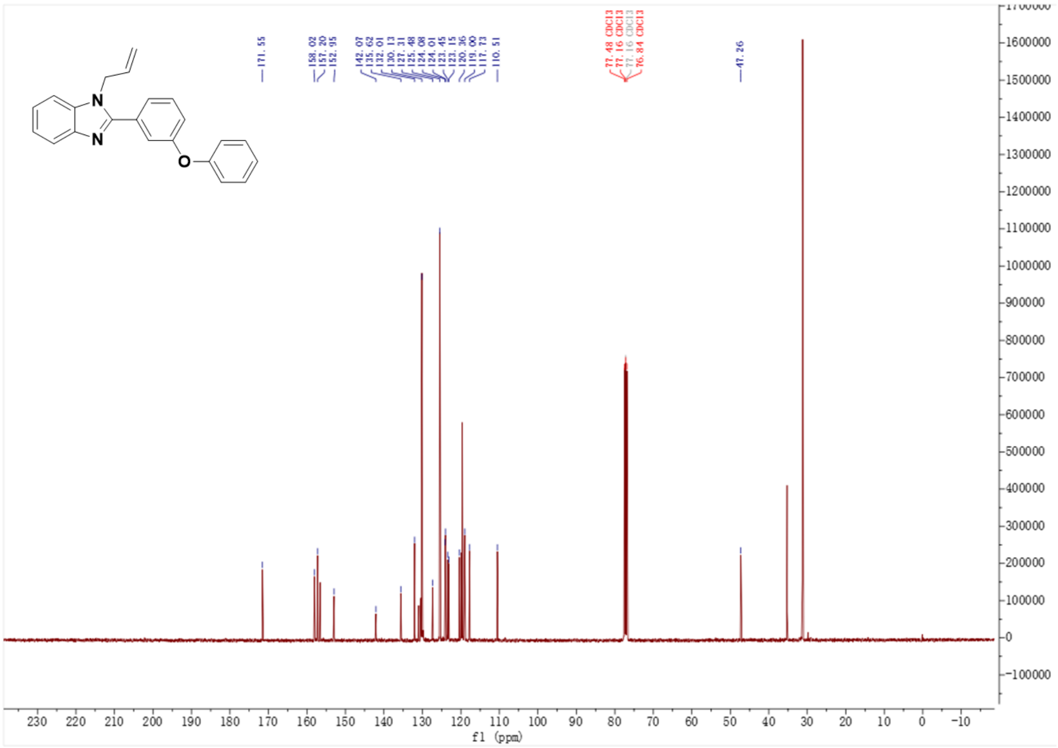

^{13}C NMR for compound **8d**.

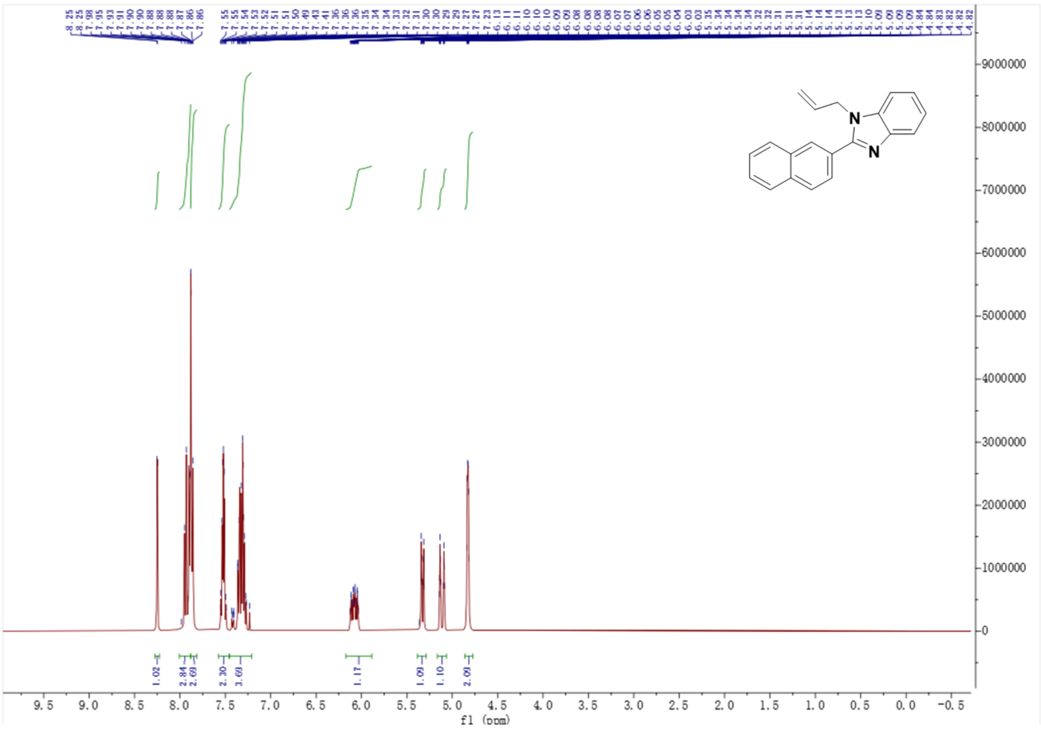

^{1}H NMR for compound **8e**.

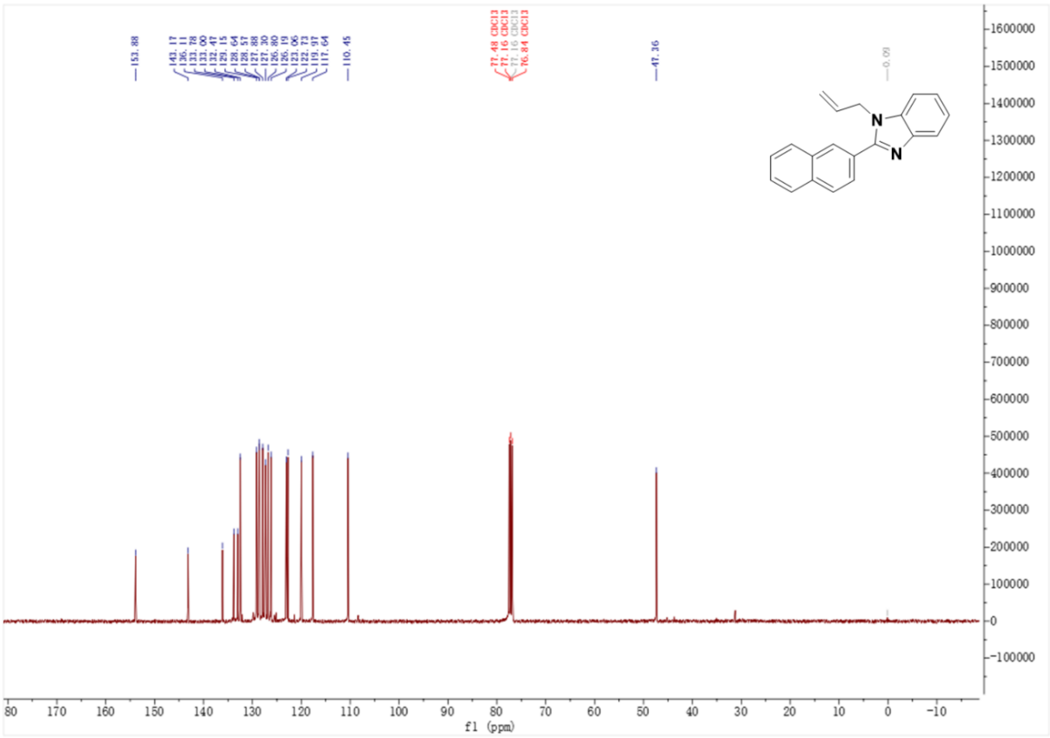

^{13}C NMR for compound **8e**.

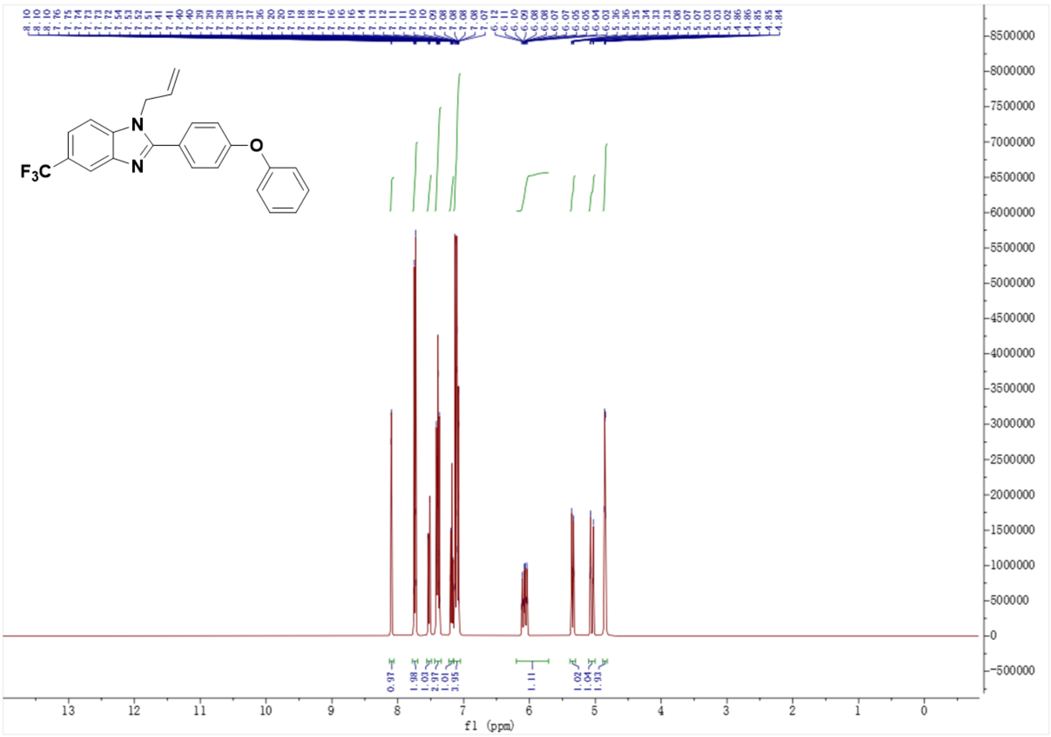

^{1}H NMR for compound **12a**.

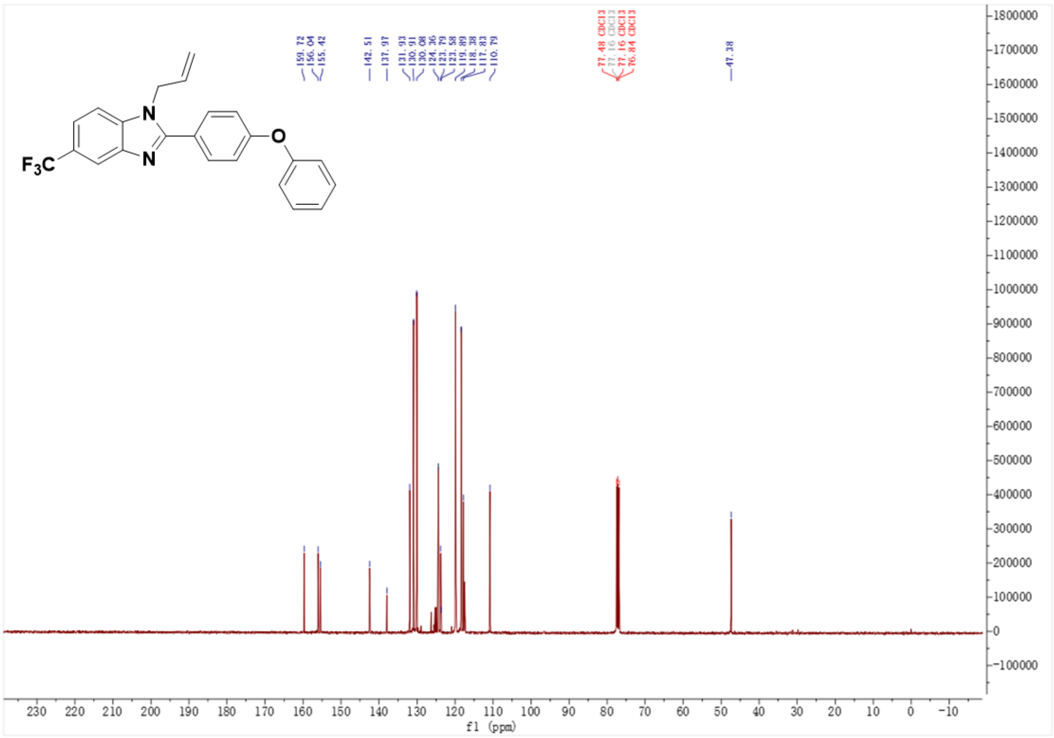

^{13}C NMR for compound **12a**.

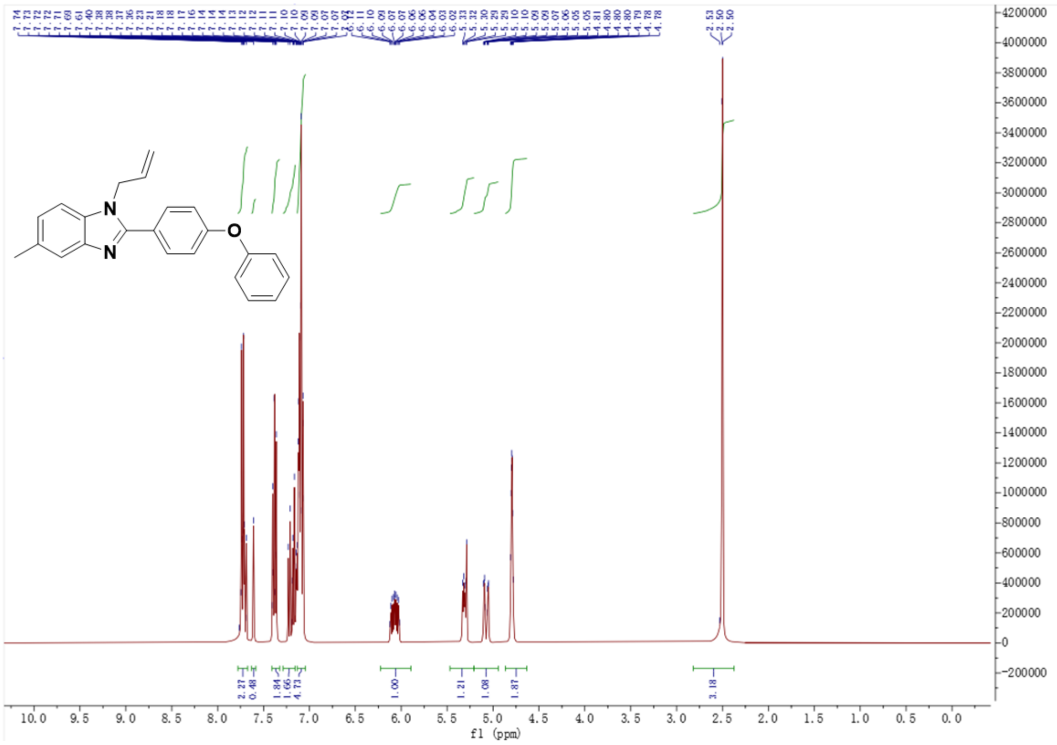

^{1}H NMR for compound **12b**.

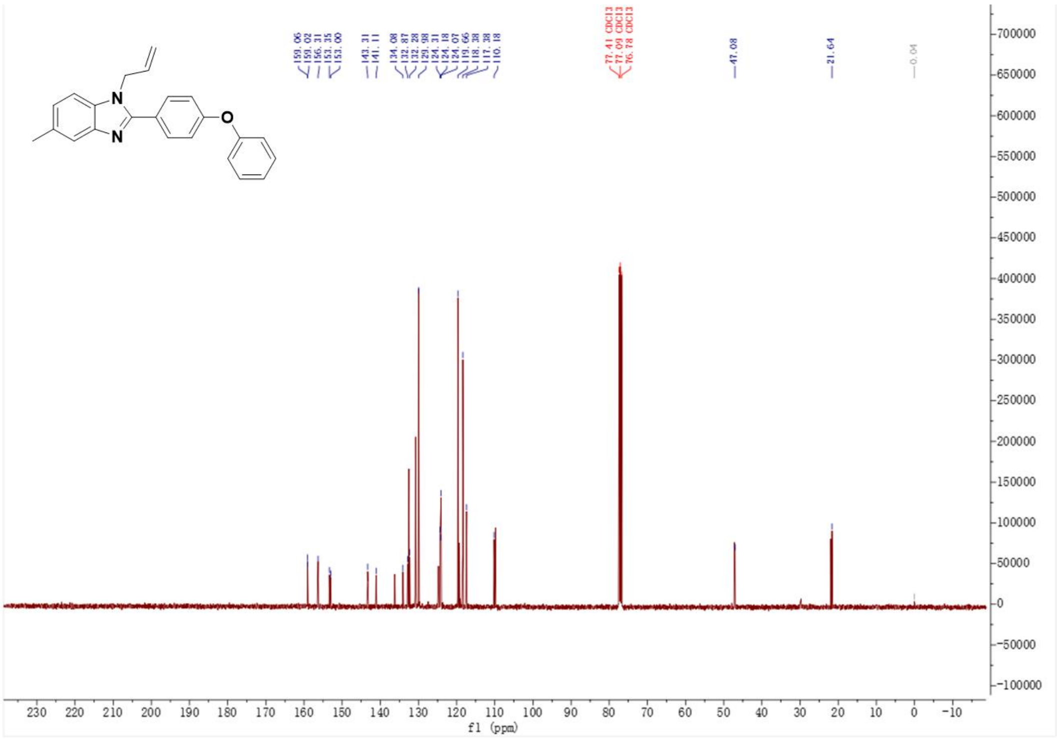

^{13}C NMR for compound **12b**.

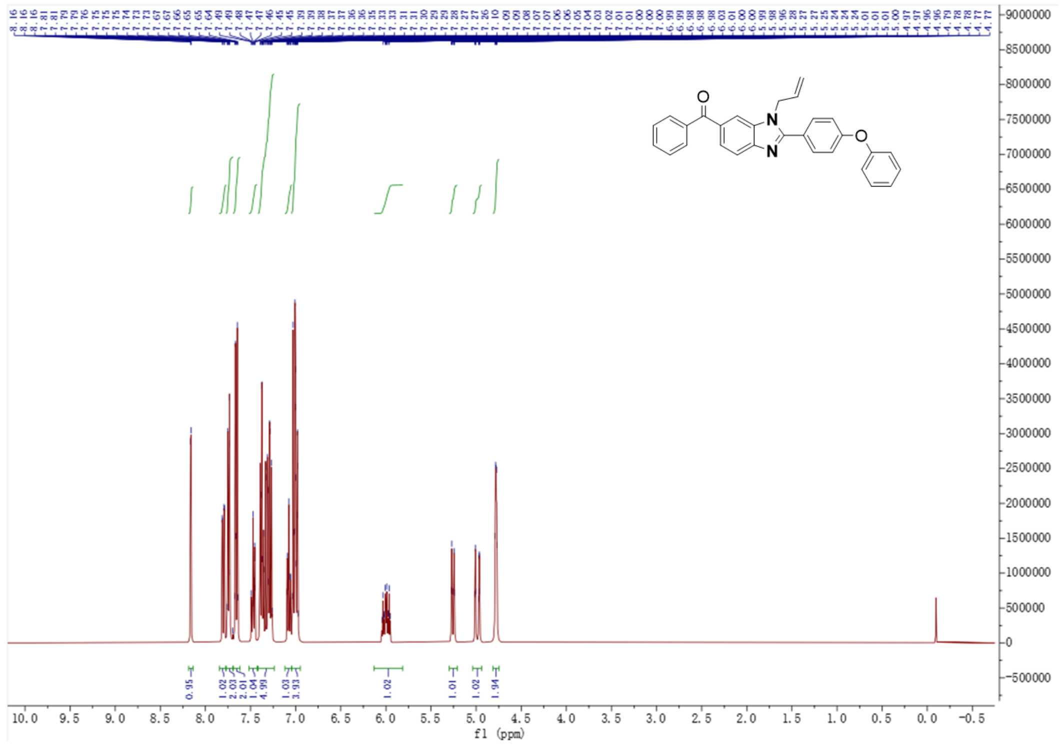

^{1}H NMR for compound **12c**.

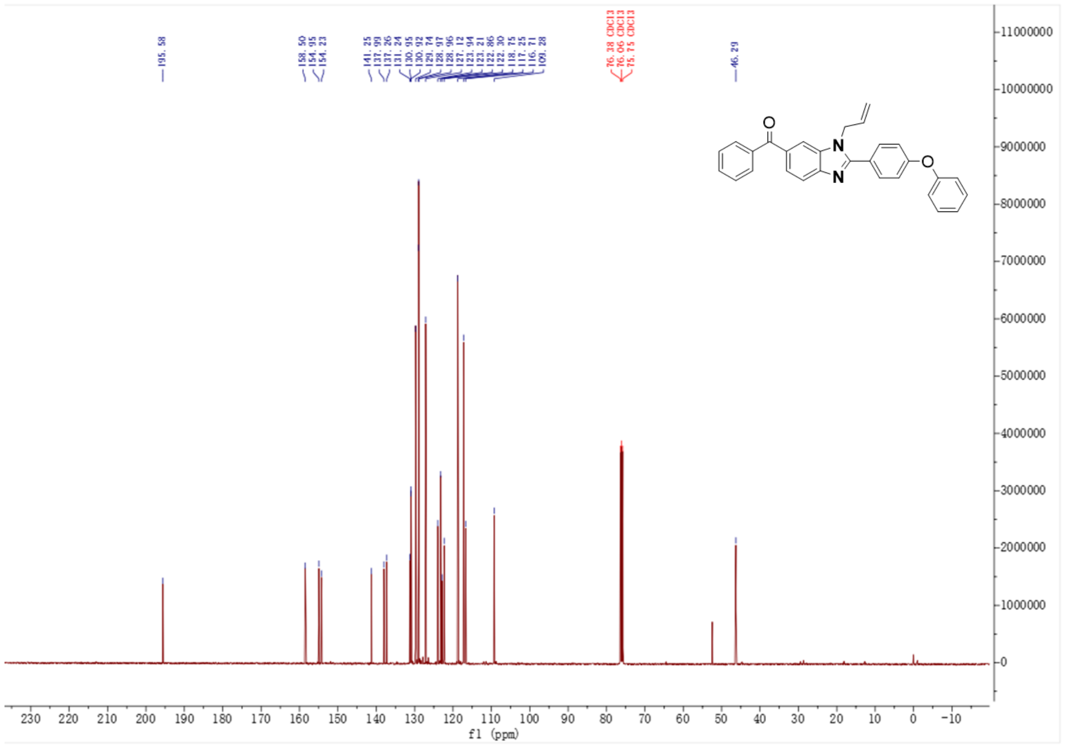

^{13}C NMR for compound **12c**.

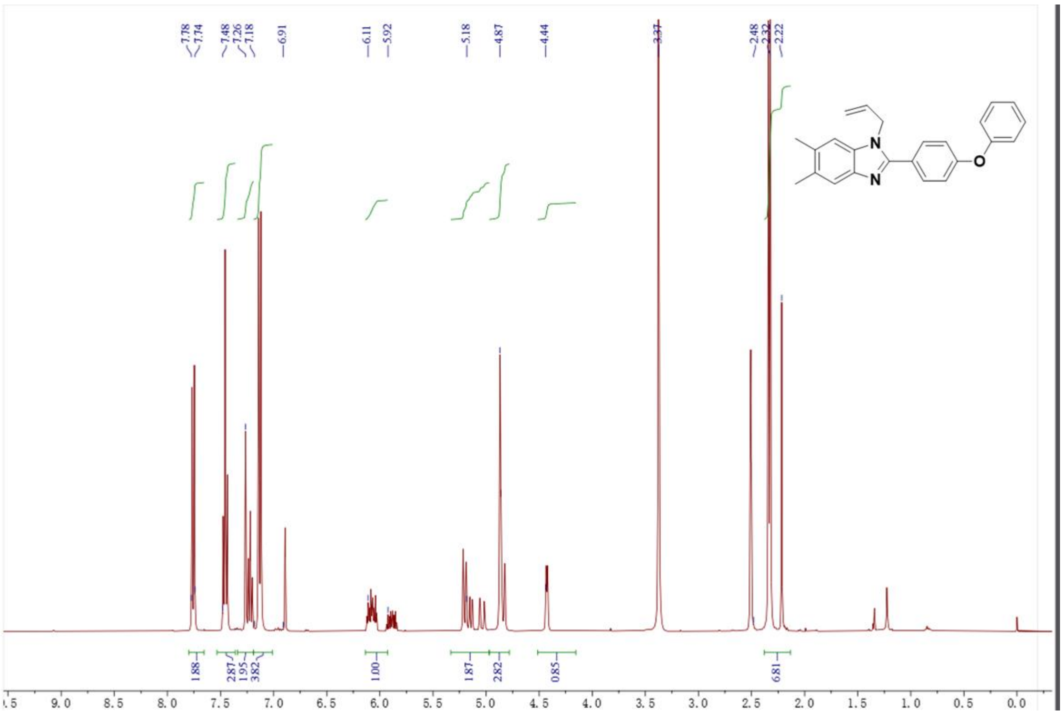

^{1}H NMR for compound **12d**.

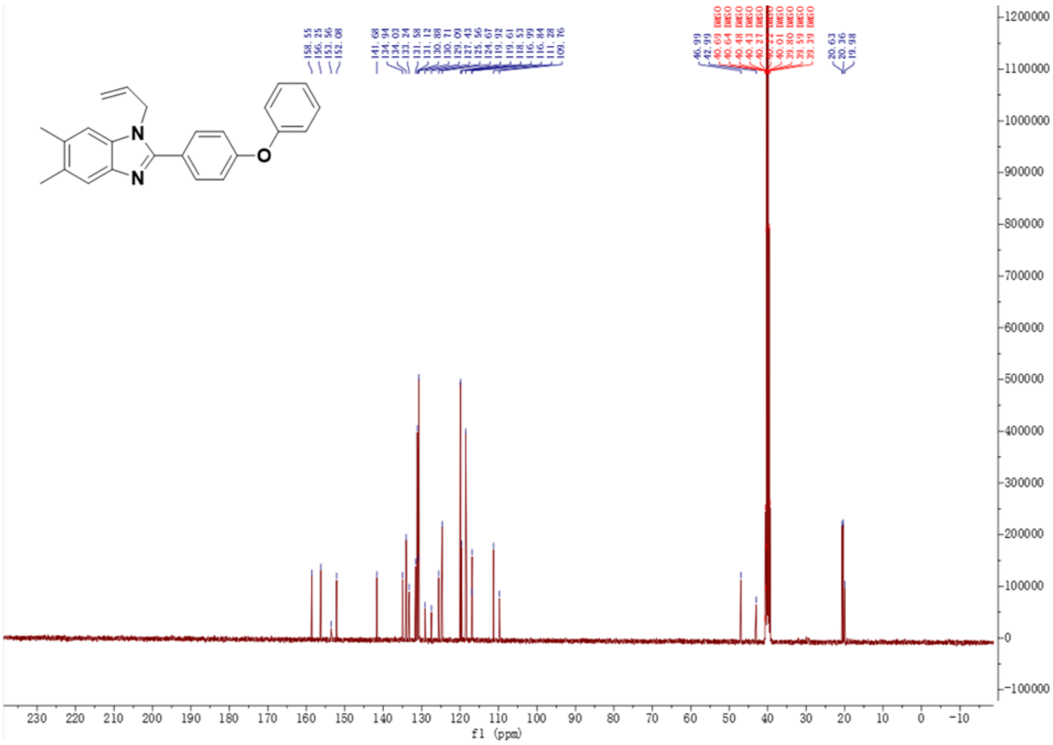

^{13}C NMR for compound **12d**.

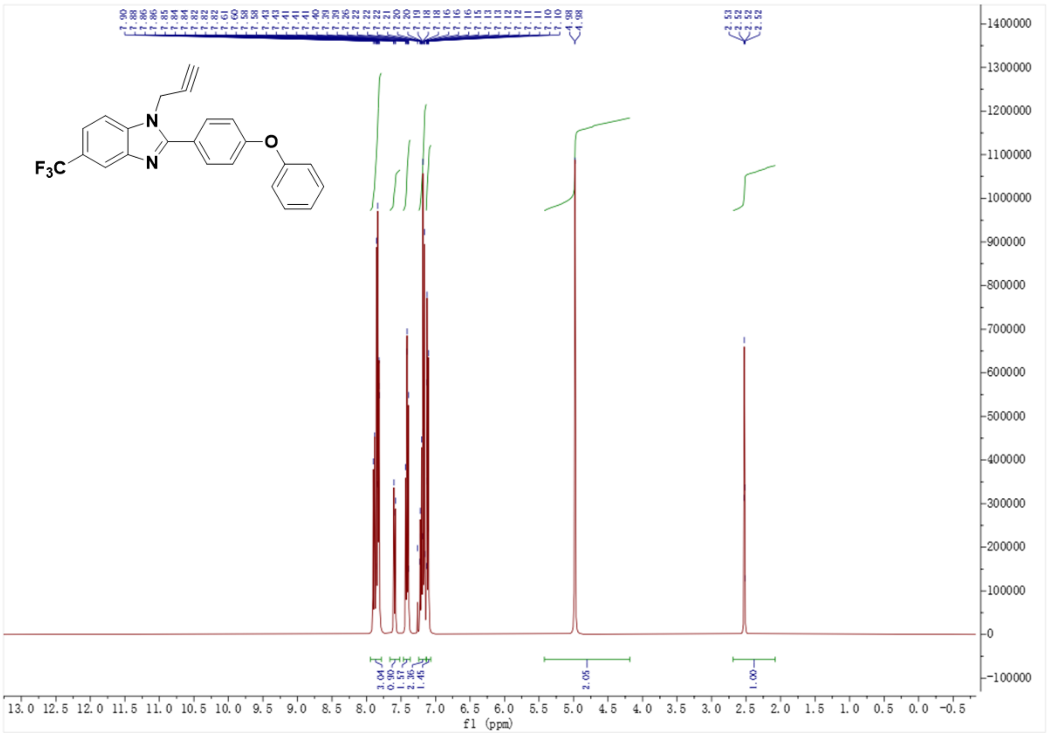

^{1}H NMR for compound **12e**.

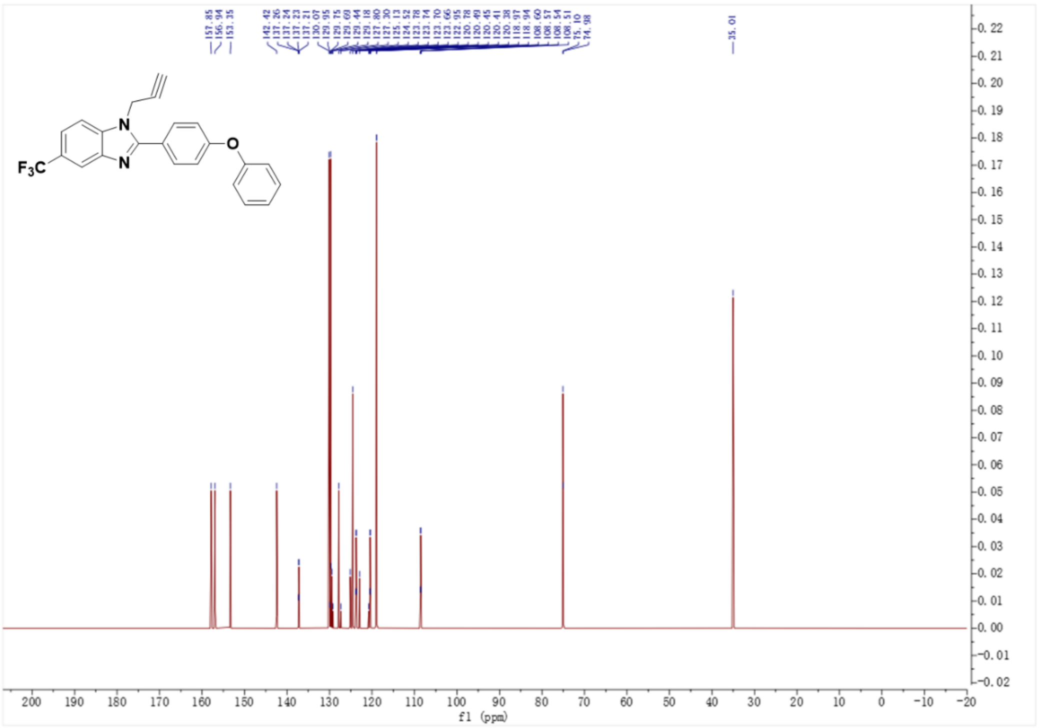

^{13}C NMR for compound **12e**.

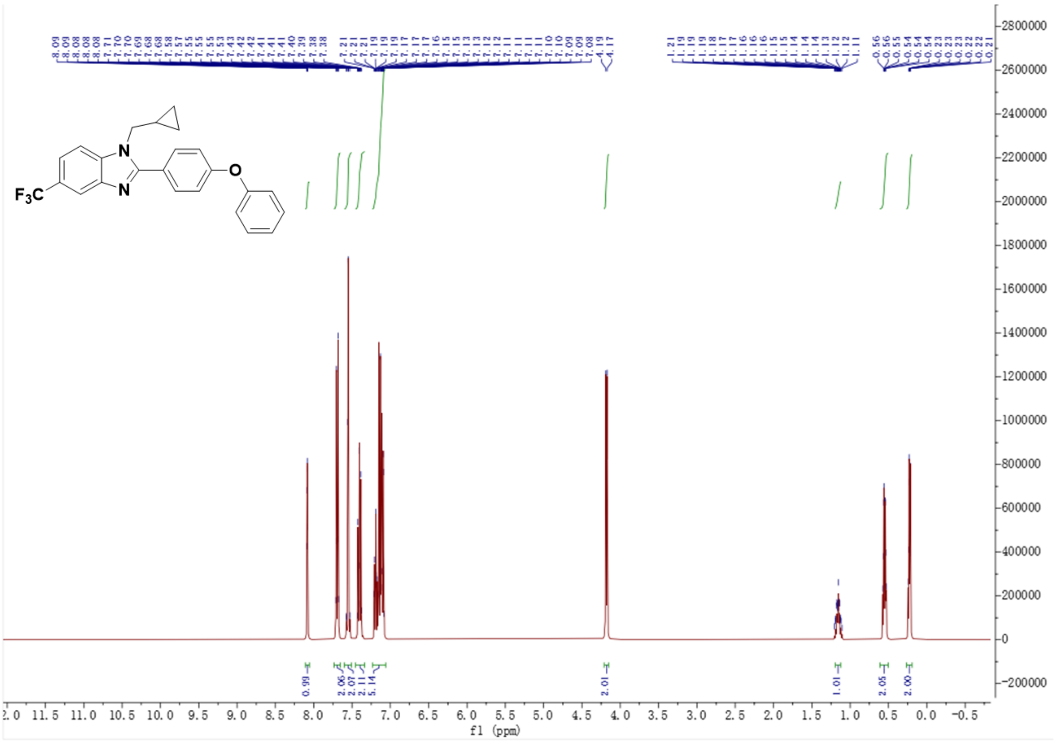

^{1}H NMR for compound **12f**.

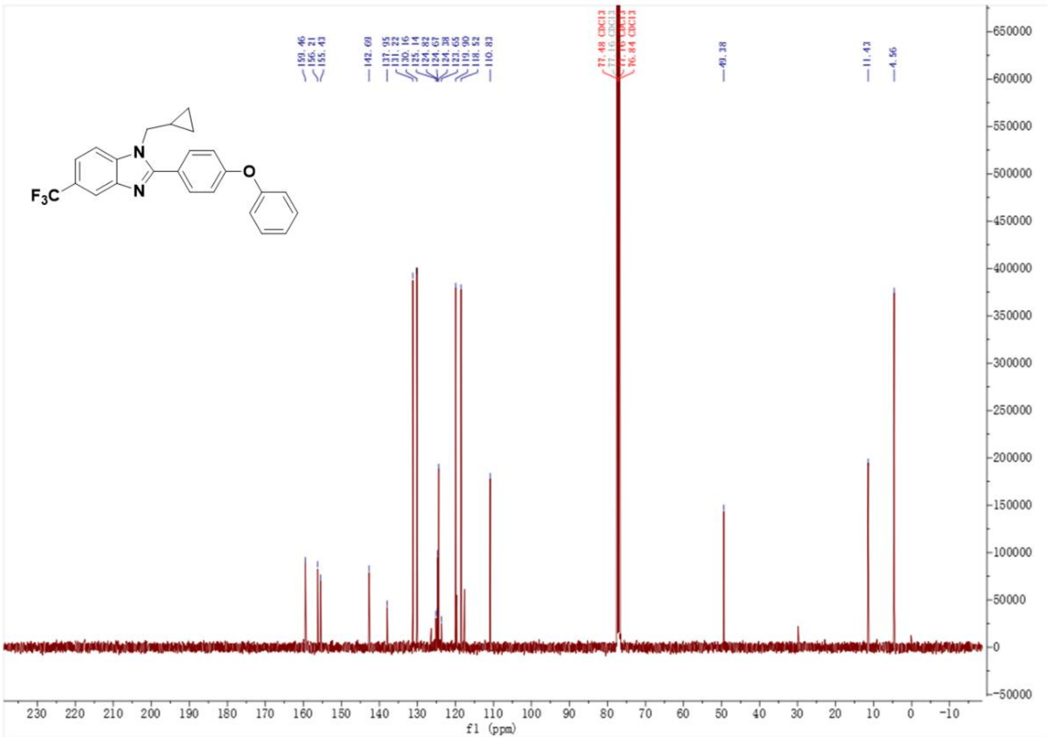

^{13}C NMR for compound **12f**.

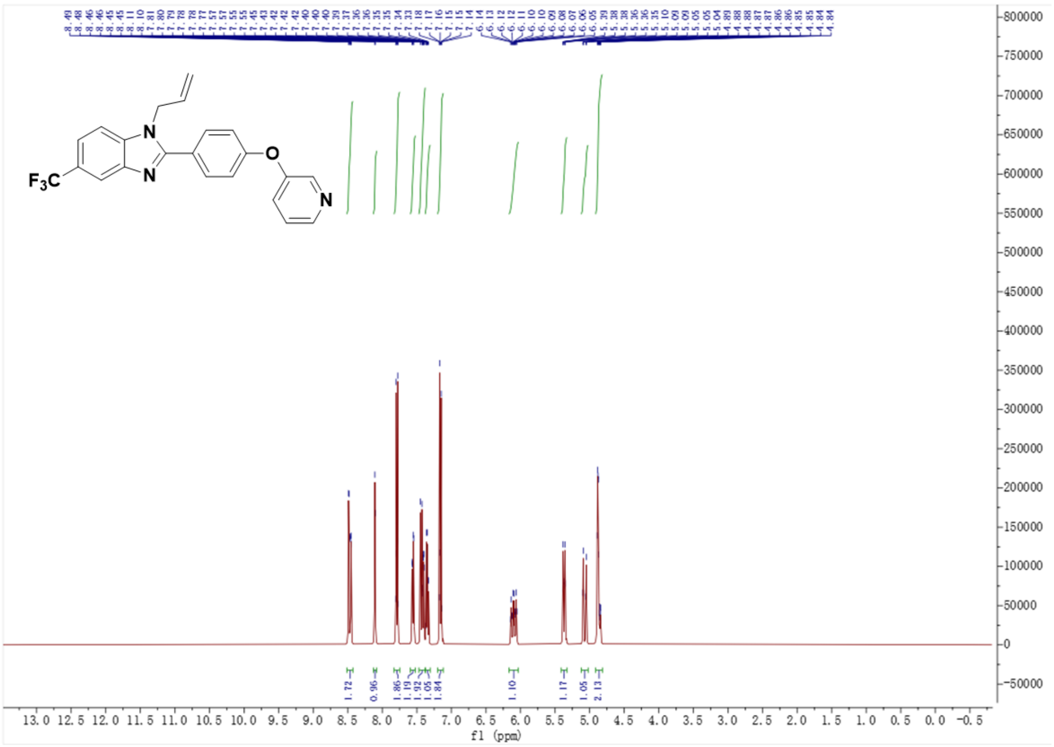

^{1}H NMR for compound **12g**.

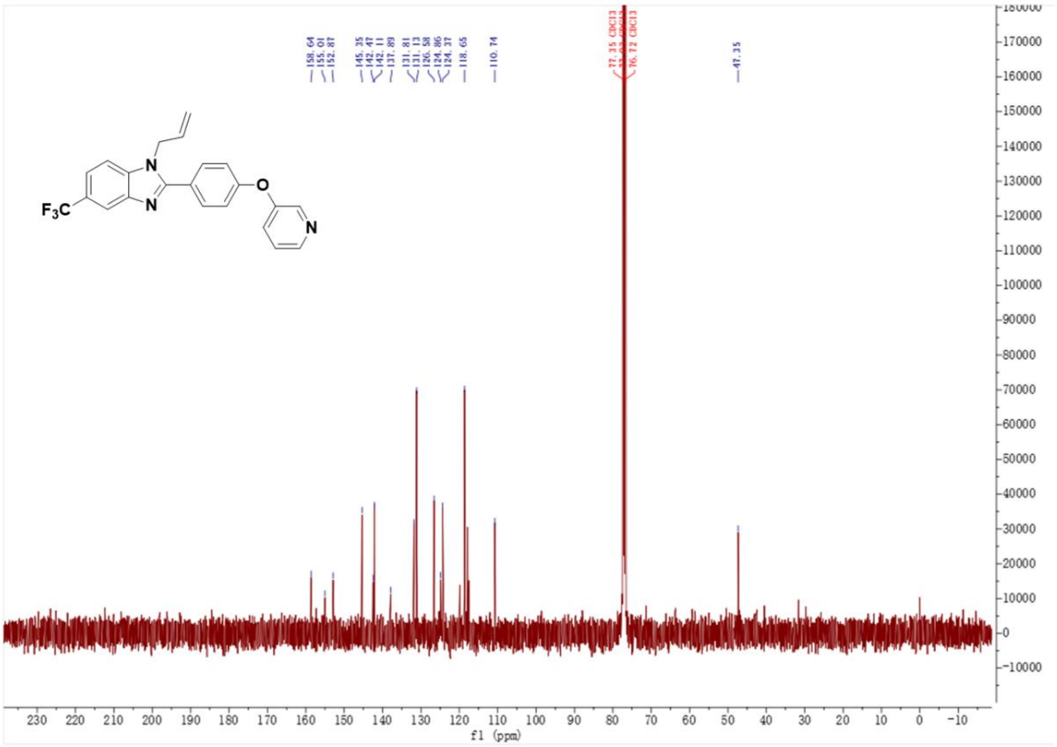

^{13}C NMR for compound **12g**.

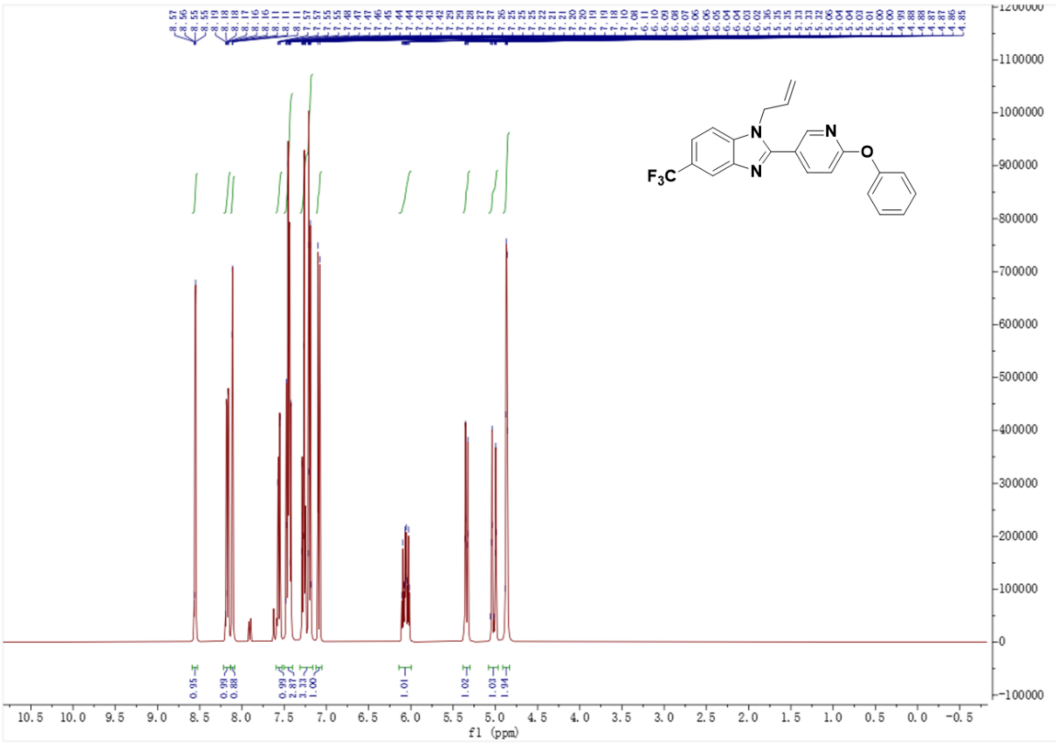

^{1}H NMR for compound **12h**.

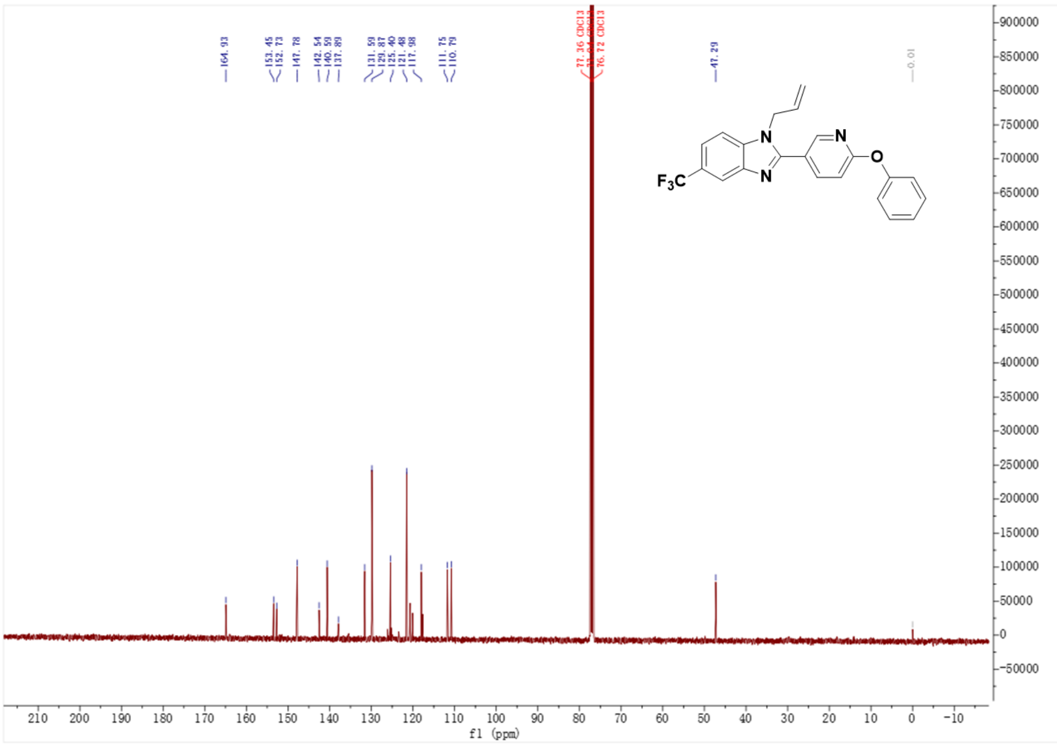

^{13}C NMR for compound **12h**.

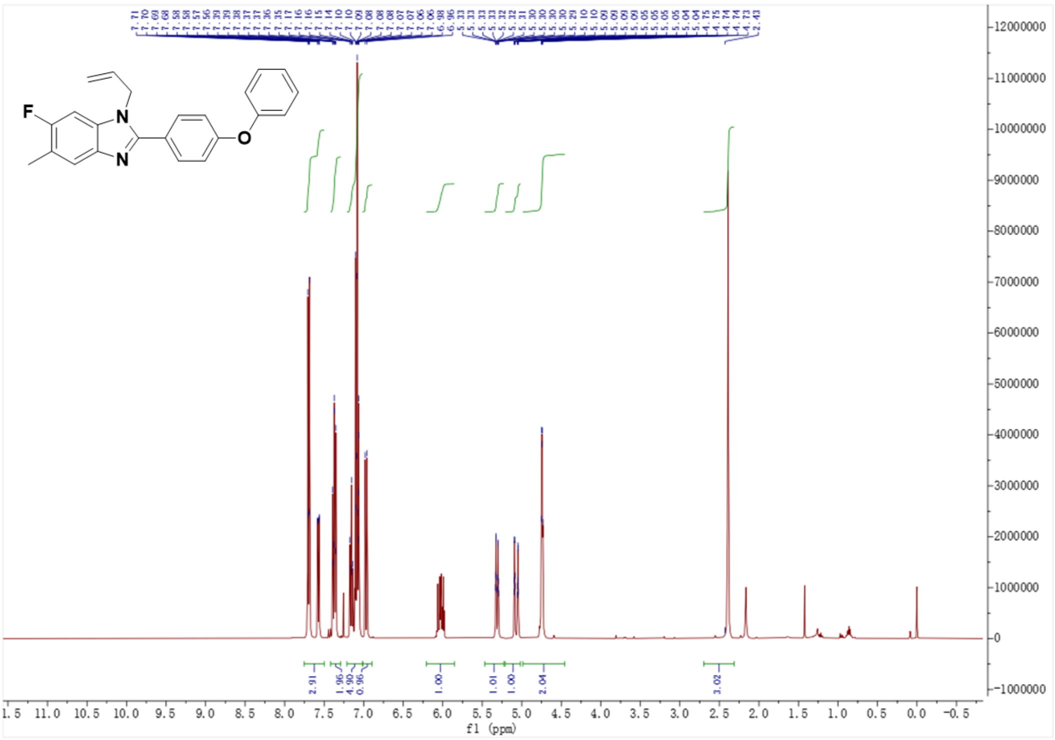

^{1}H NMR for compound **12i**.

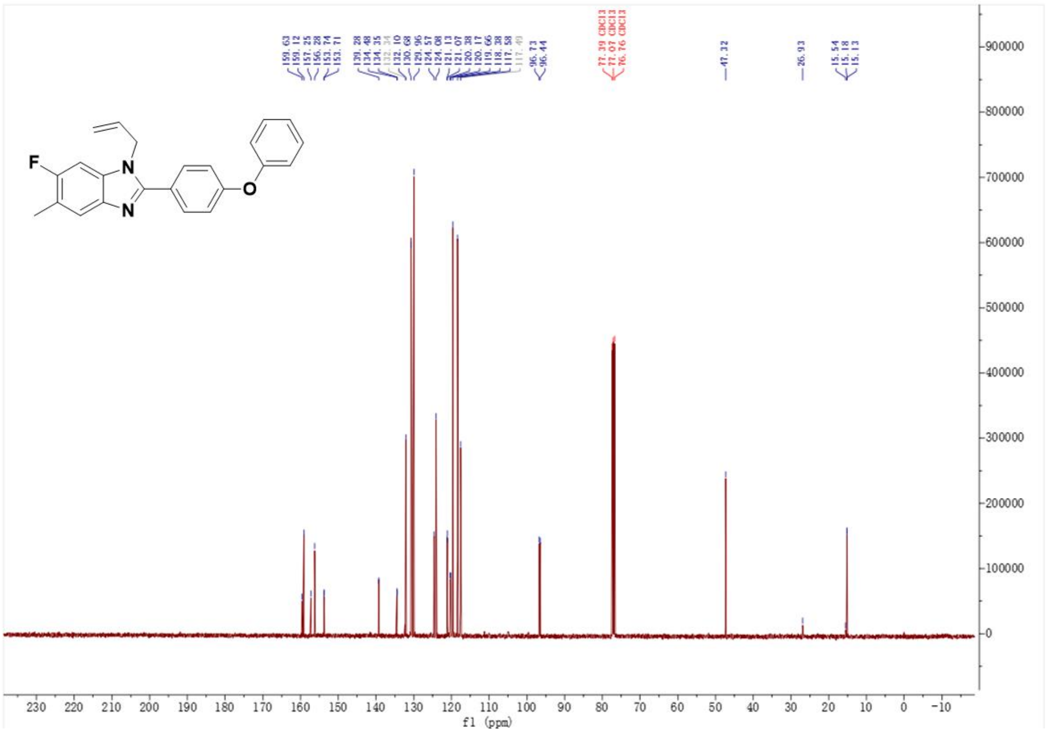

^{13}C NMR for compound **12i**.
